# Electrically Tunable Heliconical Smectic Superstructure in Polar Fluids

**DOI:** 10.1002/adma.74025

**Published:** 2026-07-09

**Authors:** Hiroya Nishikawa, Dennis Kwaria, Atsuko Nihonyanagi, Koki Sano, Hiroyuki Yoshida, Fumito Araoka

**Affiliations:** ^1^ RIKEN Center for Emergent Matter Science Wako Saitama Japan; ^2^ Department of Chemistry and Materials Faculty of Textile Science and Technology Shinshu University Ueda Nagano Japan; ^3^ School of Engineering Kwansei Gakuin University Sanda Hyogo Japan

**Keywords:** heliconical, helielectric, polar fluid, reflective color, SHG

## Abstract

The ferroelectric nematic (N_F_) phase and related polar liquid‐crystalline phases form a new class of strongly polarized yet fluid soft matter. Well‐recognized heliconical ferroelectric phases such as N_TBF_ and ${\mathrm{SmC}}_{P}^{H}$ exist, but their electro‐optic functionality in smectic systems has been largely unexplored. In this study, we report a newly designed single‐component achiral molecule exhibiting a hierarchical polar phase sequence: SmA_F–_N_TBF_–HEC–${\mathrm{SmC}}_{P}^{H}$. The key feature of the ${\mathrm{SmC}}_{P}^{H}$ phase is its ability to form a stable macroscopic orientation without any alignment layers and to enable continuous, reversible pitch modulation over a wide spectral range at ultralow electric fields, approximately one‐third of those required for conventional heliconical nematics. The system exhibits characteristic electro‐optic properties in the sub‐kilohertz range, differentiating it from heliconical materials controlled by dielectric–elastic balance at higher frequencies. In addition, the ${\mathrm{SmC}}_{P}^{H}$ phase's polar heliconical smectic structure facilitates enhanced second‐harmonic generation via favorable phase matching, thereby establishing a simple and robust platform for low‐voltage photonic applications.

## Graphical abstract

Strong dipole–dipole interactions give rise to an emergent polar smectic phase with spontaneous chiral symmetry breaking. This resulting polar heliconical smectic superstructure enables novel functionalities in polar fluids, such as color modulation driven by electric field strength and frequency, as well as second‐harmonic generation amplification within the unique polar heliconical architecture.

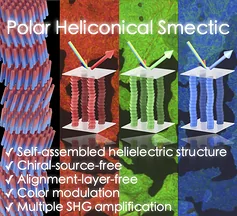

## 1 Introduction

**FIGURE 1:**
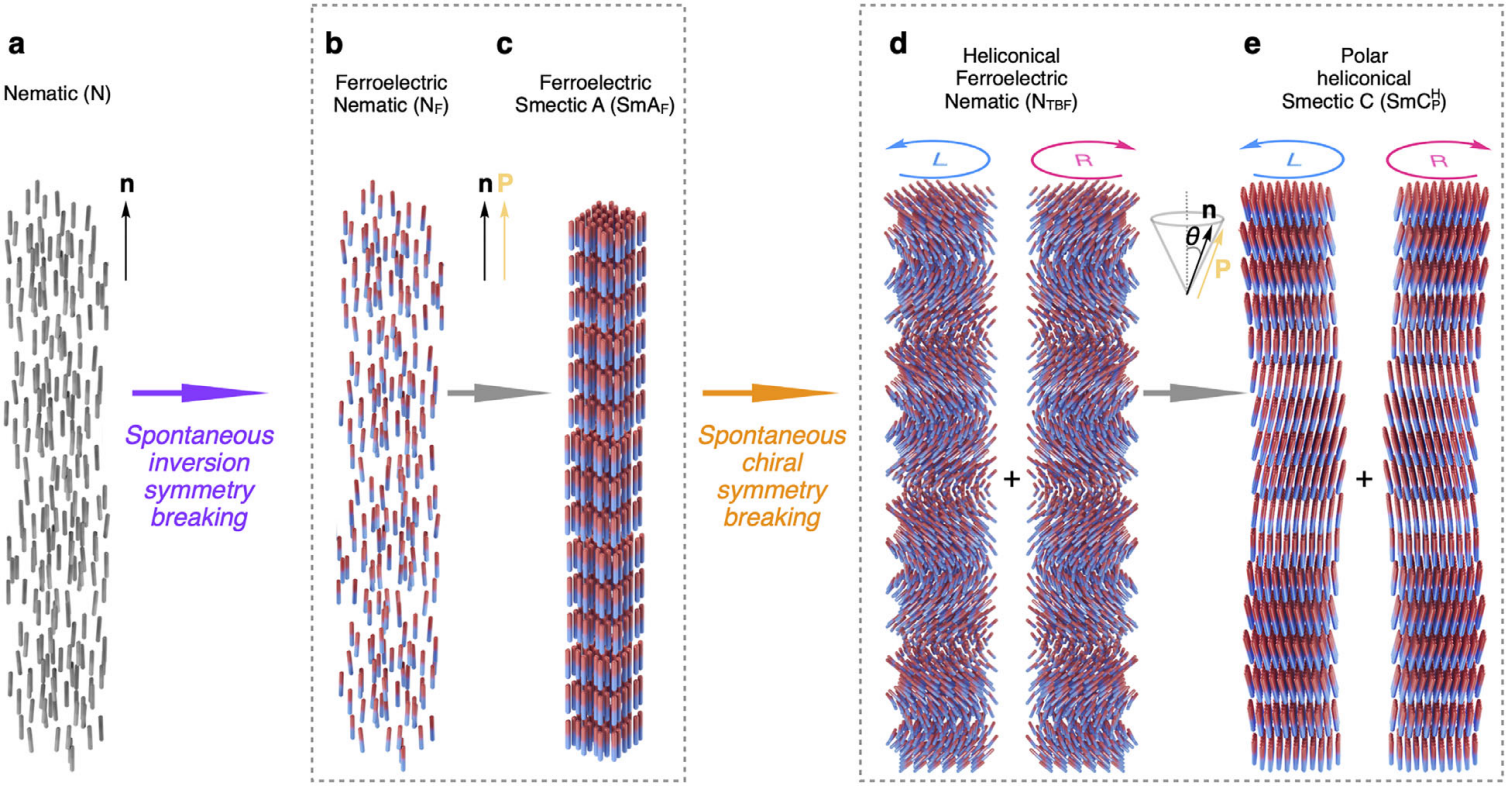
Schematic of the phase transition sequence for a polar material via hierarchical symmetry breaking (n: director and P: polarization vector). All phases except for the nematic (N) phase lack a head–tail orientation equivalence, so polar order occurs (i.e., n ≠ −n). The N_F_ phase emerges via spontaneous inversion symmetry breaking from the isotropic or N phase. The same pathway offers a transition to the SmA_F_ phase with additional layer positional ordering. Subsequent spontaneous chiral symmetry breaking generates unique helielectric phases (i.e., N_TBF_ and ${\mathrm{SmC}}_{P}^{H}$) in which n and P are tilted at an angle 0 < ***θ*** < ***π***/2 with respect to the helical axis. In both phases, left (*L*)‐ and right (*R*)‐handed helices are formed with equal probability.

Since its discovery in 2017 [1, 2, 3], the ferroelectric nematic (N_F_) liquid crystal (LC) has been established as a distinct state of fluid matter, where ferroelectric order coexists with high fluidity. In the N_F_ phase, the head–tail symmetry of the nematic director (**n** = −**n**) present in a conventional uniaxial nematic (N, Figure 1a) phase is broken, resulting in a polar structure characterized by aligned director and polarization vectors (n≠−n **, n**∥**P**), where **P** denotes the polarization vector (Figure 1b) [3, 4, 5]. This symmetry breaking gives rise to exceptionally high polarization densities and strong nonlinear optical responses while preserving fluidity, thereby underpinning a growing family of polar ferroelectric and helielectric LC phases [4, 5].

Following the discovery of the N_F_ phase, various related polar LC phases have been identified, introducing hierarchical symmetry breaking into polar fluids through the successive emergence of additional chirality and/or smectic positional order (Figure 1a–e). When smectic layering develops from the N_F_ phase, a ferroelectric smectic A (SmA_F_) phase appears (Figure 1c), representing a polar analog of the conventional orthogonal smectic A (SmA) phase with **n**∥**P** [6, 7, 8]. Further structural complexity arises when molecular tilt develops within the smectic layers, leading to the ferroelectric smectic C (SmC_F_) phase with **n**∥**P** [8, 9, 10].

In parallel, the introduction of chirality gives rise to helicoidal superstructures. The chiral nematic (N*) phase is a well‐established, nonpolar LC state formed by induced chirality from intrinsically chiral mesogens or chiral dopants [11]. In contrast, the chiral N_F_ (N_F_*) phase represents the polar counterpart of N*, where polar helicoidal order arises from the coupling between polar order and chirality. Similar to the N* phase, this phase can be realized either by designing intrinsically chiral polar molecules [12, 13] or by introducing chiral dopants [14, 15] into the N_F_ phase. In the N_F_* phase, inversion symmetry is broken, and the polar structure is inherently chiral.

In 2024, two independent studies demonstrated that achiral molecular systems can spontaneously break chiral symmetry, giving rise to heliconical ferroelectric phases in the absence of molecular chirality. One manifestation of this phenomenon is the heliconical ferroelectric nematic phase (N_TBF_) [7, 16], which represents a striking example of spontaneous chiral symmetry breaking within the N_F_ phase. This concept was further extended to smectic systems with the discovery of the polar heliconical smectic C phase (${\mathrm{SmC}}_{P}^{H}$), which combines long‐range smectic layering, molecular tilt, and heliconical polar order [17, 18]. Together with the SmA_F_ and SmC_F_ phases reported in 2022 and 2024, these discoveries establish a hierarchical symmetry‐breaking framework in polar fluids. Along this progression, an intermediate polar heliconical phase—referred to as the helielectric conical (HEC) mesophase—has also been reported [19]. This phase is defined as a polar state with a heliconical structure exhibiting SmC‐like local lamellar ordering (Note S1).

From an application perspective, helical polar phases with pitches in the visible light wavelength range have attracted growing attention as photonic materials. For the N_F_* and N_TBF_ phases, electric‐field‐induced [20] and photo‐induced [21] color modulation, as well as laser oscillation [22, 23], have been demonstrated. In contrast, previous studies on the ${\mathrm{SmC}}_{P}^{H}$ phase have primarily focused on electric‐field‐induced reorientation of the helical axis [18], whereas systematic investigations of electric‐field‐driven modulation of the heliconical pitch itself remain scarce. Consequently, continuous pitch tunability, reflective color modulation, and nonlinear optical functionality in the ${\mathrm{SmC}}_{P}^{H}$ phase remain largely unexplored.

**FIGURE 2:**
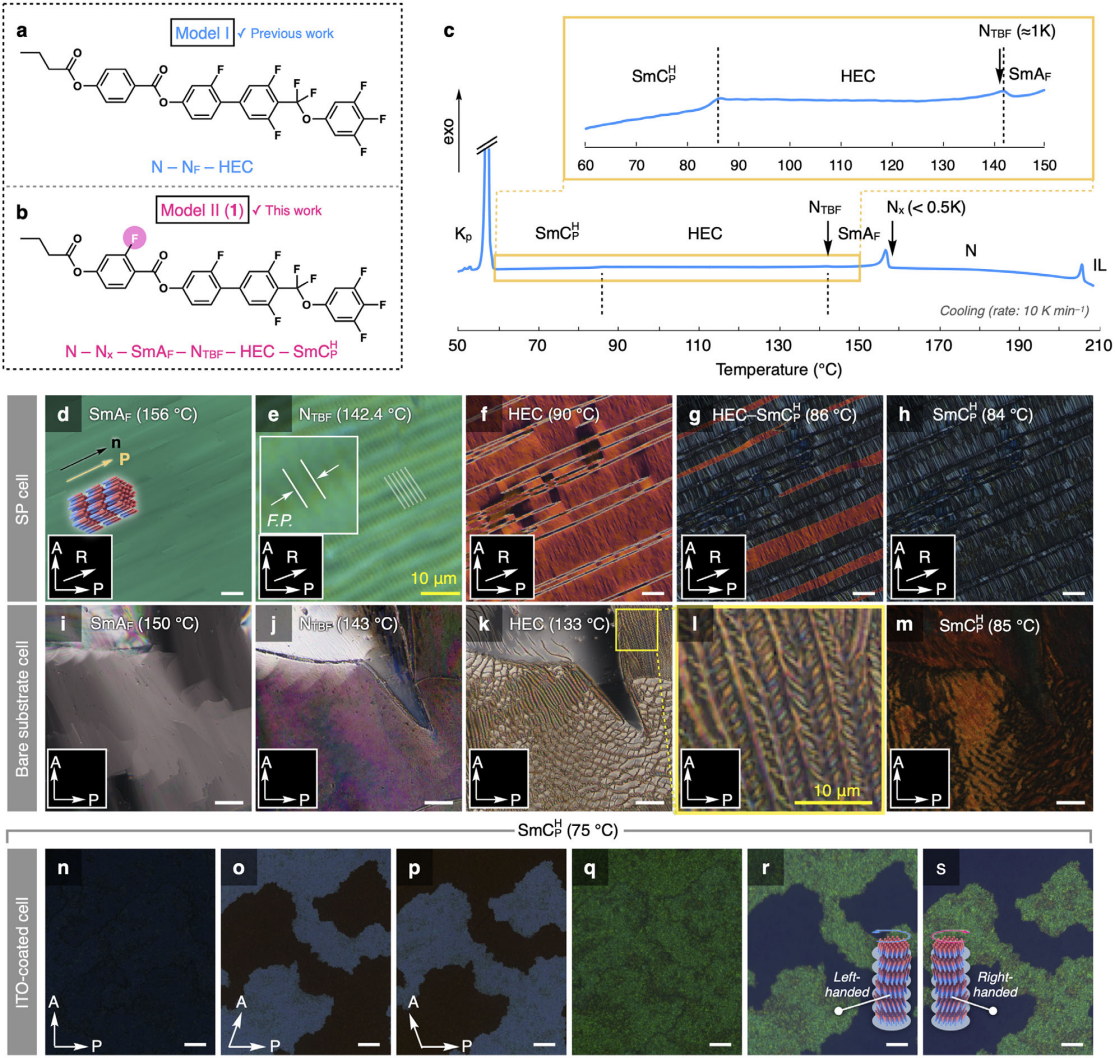
Phase transition behavior for **Compound 1**. Chemical structures used in the (a) previous study (Model I) and (b) present study (Model II or **Compound 1**). (c) DSC curves during cooling (scan rate: 10 K min^−1^). POM images taken under cross polarizers in SP (d–h), bare substrate (i–m), and ITO‐coated glass (n–s) cells at various temperatures. Thickness: SP cell: 5.0 µm; bare substrate cell: 25 µm; ITO cell: 4.6 µm. Reflection‐mode POM images of the ${\mathrm{SmC}}_{P}^{H}$ phase (*T* = 75°C) (q) without a circular polarizer and with (r) left‐handed and (s) right‐handed circular polarizers. The inset in the panel (e) shows the enlarged photograph, in which the distance between dark lines corresponds to the full pitch (F.P.) of N_TBF_ phase. The inset in panel (d) shows a schematic illustration of the SmA_F_ phase. In the SP cell, smectic blocks lay on the substrate, and molecules were aligned along the rubbing. The insets in panels (r) and (s) show schematic illustrations of left‐ and right‐handed polar heliconical structures. Scale bars: 100 µm (except the panels (e) and (l)).

Therefore, the central motivation of this work is to address the following question: can polar heliconical phases serve as electrically tunable photonic media beyond currently reported electro‐optic responses? To this end, we build on a previously reported polar molecule (Model I, Figure 2a) exhibiting the HEC phase [19] and design a new single‐component achiral molecule (**Compound 1**, Figure 2b; see Figures S1–S4, for characterization spectra) through targeted molecular modification, involving lateral fluorination. The resulting material exhibits a rich phase sequence upon cooling (N–N_X_–SmA_F_–N_TBF_–HEC–${\mathrm{SmC}}_{P}^{H}$), reflecting hierarchical symmetry breaking. Notably, the ${\mathrm{SmC}}_{P}^{H}$ phase in **Compound 1** forms a stable macroscopic orientation under an applied electric field(*E*‐field) without alignment layers. More importantly, it enables continuous *E*‐field‐driven heliconical pitch modulation at ultralow voltages and in the subkilohertz frequency regime, accompanied by vivid reflective color control and enhanced second‐harmonic generation (SHG). To the best of our knowledge, such continuous and reversible pitch modulation with visible reflective color tuning has not been reported for previously known ${\mathrm{SmC}}_{P}^{H}$ materials. These combined features distinguish the present material from previously reported heliconical systems and reveal a dynamically mediated route to electrically tunable structural color in heliconical smectic systems.

## 2 Results and Discussion

### 2.1 Phase Transition Behavior

The differential scanning calorimetry (DSC) curve (Figures 2c and S5; Table S1) and polarized optical microscopy (POM) images of **Compound 1** (Figures 2d–s and S6) were used to characterize its phase transition behavior. For POM studies, we used syn‐parallel rubbing polyimide (SP) glass cells (Figure 2d–h), bare substrate cells (Figure 2i–m), and indium tin oxide (ITO)‐coated glass cells (Figure 2n–s). In the SP cell, cooling from the nematic (N) phase caused many defects (banded texture) to appear along the rubbing direction (Figure S6b), which then developed into uniform block‐like domains aligned in the rubbing direction (Figure 2d). These features are typical in the N_F_ phase transiting into the SmA_F_ phase. However, this phase (hereinafter referred to as the N_X_ phase) exhibited a high apparent relative dielectric permittivity ($\varepsilon_{\mathrm{app}}^{\prime }$ = 5.2 × 10^3^) (vide infra) despite showing an antiferroelectric‐like electrical response (Figure S16), suggesting antiferroelectric, ferrielectric, or relaxor‐like [24] behavior. Due to its extremely narrow temperature range, no further classification is performed. Further cooling revealed a stripe texture along the rubbed direction, with additional finer stripes appearing perpendicular to the initial stripes (Figure 2e). Within this temperature range, colored reflection or scattering was visible in the LC cell. This was more obvious in a bare substrate cell (Figure 2j). The full pitch of the fine stripes corresponds to that of the heliconical structure, which was estimated to be 1.3 µm (Figure S7). Typical POM textures reported for the N_TBF_ phase were observed in the present study [7, 16]. In the bare‐substrate cell, the appearance of a vivid selective‐reflection color is consistent with a heliconical structure whose helical axis tends to align nearly normal to the substrates, thereby enhancing selective reflection. In the SP cell, stripe textures aligned along the rubbing direction are observed, consistent with a heliconical structure with the helical axis lying in‐plane. These characteristic textures are in good agreement with our experimental observations and support the assignment to the N_TBF_ phase. Note that this assignment is further supported by the combined evidence from x‐ray diffraction (XRD), broadband dielectric spectroscopy (BDS), polarization reversal current (PRC) and SHG measurements presented in the subsequent sections. It is noteworthy to mention that Strachan et al. discussed the possible competition of the SmA_F_ and N_TBF_ phases [25], and that no N_TBF_–SmA_F_ phase transition within a single component molecule has yet been reported to date. The N_X_–SmA_F_–N_TBF_ phase transition in **Compound 1** is obviously one of the rare examples of the reentrant phenomenon in polar phases [26], where the restoration of nematic order follows the collapse of smectic layer order, although the mechanism of this is not yet clear. The N_TBF_ phase existed only within a narrow temperature range of approximately 1 K, after which inhomogeneous textures of the HEC phase appeared (Figures 2e,f and S8a–c). Such a textural change was also observed in thicker/thinner bare substrates(Figures 2j–l and S8d–f) and thinner ITO cells (Figure S8g–i). Interestingly, in the bare substrate cell, a coil‐like spiral filament texture was observed in the same temperature region (Figure 2k,l). The similar spiraling filaments were also observed in the HEC phase in our previous study [19]. In the SP cell, the faint reflective color was red‐shifted upon cooling, and eventually the texture transited into a darker state at 84°C (Figures 2g,h and S6f–l). A texture with low birefringence was also observed at 85°C in the bare substrate cell (Figure 2m). The ITO‐coated cell within this temperature range displayed sub‐millimeter‐sized island domains in which the contrast between adjacent domains was reversed by decrossing the polarizers in the opposite directions, indicating optical activity (Figure 2n–p). Furthermore, reflection microscopy confirmed reflective colors (Figure 2q–s) of left‐ or right‐handed circularly polarized selective reflection. Given the generation of enantiomeric domains and the observation of selective reflective color, the low‐temperature phase appearing below the HEC phase was considered to be the ${\mathrm{SmC}}_{P}^{H}$ phase. So far, the control over both the reflective color and the orientation of the helical axis in the ${\mathrm{SmC}}_{P}^{H}$ phase is yet difficult [17, 18]. In contrast, the ${\mathrm{SmC}}_{P}^{H}$ phase in **Compound 1** enables both *E*‐field‐induced reorientation of the helical axis and color modulation via the helical pitch variation, as will be demonstrated below.

### 2.2 Structural Characterization

**FIGURE 3:**
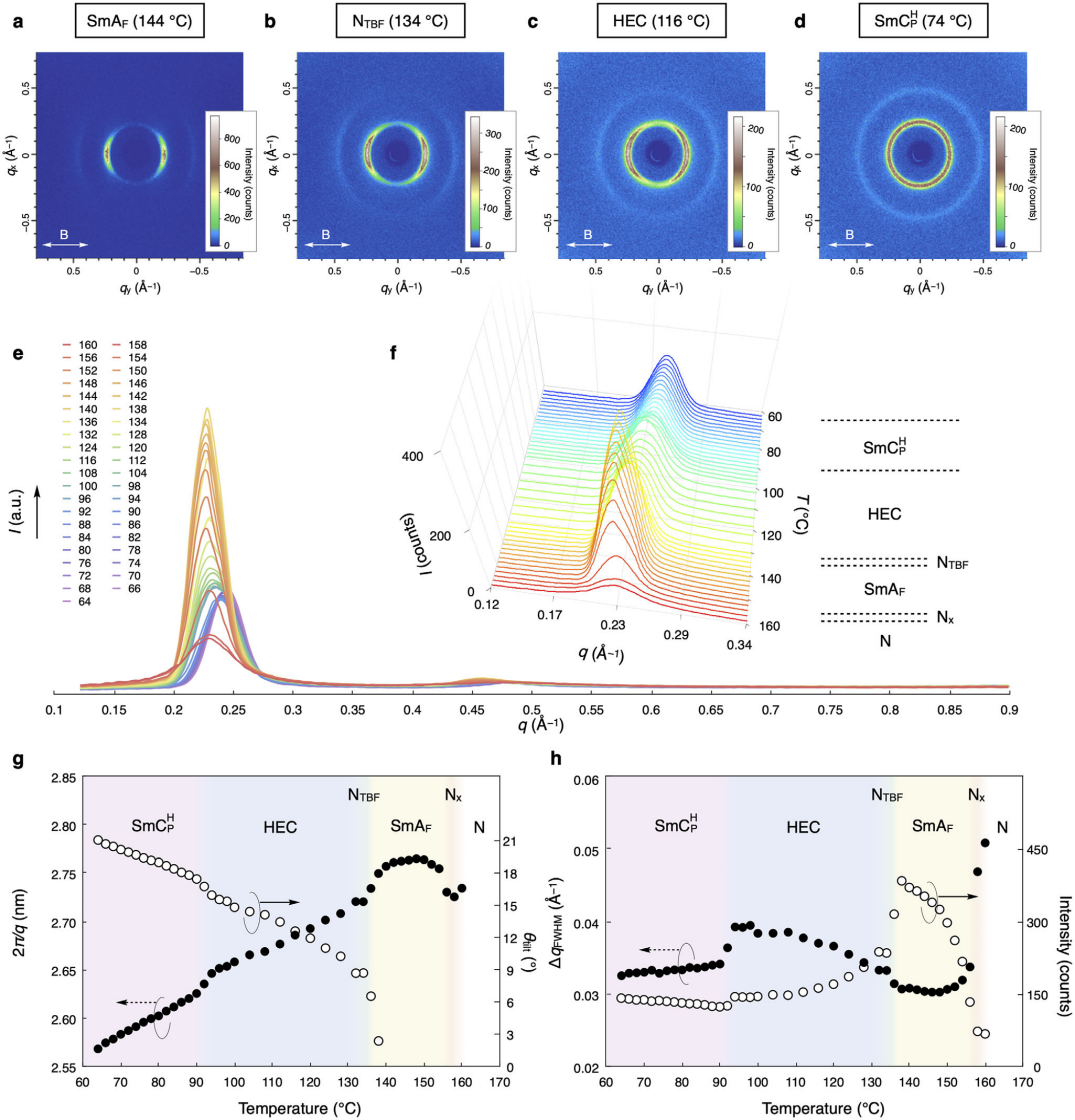
XRD profiles for **Compound 1**. 2D WAXS patterns obtained under a magnetic field (∼1 T, B∥n) of the (a) SmA_F_, (b) N_TBF_, (c) HEC, and (d) ${\mathrm{SmC}}_{P}^{H}$ phases. (e) 1D and (f) 3D x‐ray diffractograms generated by box‐scan analysis of the 2D WAXS profile. (g) 2*π*/*q* and tilt angle (*θ*_tilt_) vs temperature. (h) ∆*q*_FWHM_ and intensity vs temperature.

Two‐dimensional (2D) wide‐angle XRD patterns were obtained for structural characterization of **Compound 1** at various temperatures (Figure 3a–d; Figures S9 and S10). The corresponding 1D XRD plots were generated by the box scan analysis (Figure S11) along the equator parallel to the applied magnetic field **B** (**n**∥**B**) of the 2D XRD pattern (Figures 3e,f and S12). The following parameters were then obtained as functions of temperature: *d*‐spacing (*d* = 2*π*/*q*) (Figure 3g), tilt angles (*θ*_tilt_) (Figure 3g), full width at half maximum (FWHM) of the scattering vector, *q* (∆*q*_FWHM_) (Figure 3h), and the peak intensity (Figure 3h). The diffraction pattern of the N phase can be attributed to short‐range positional ordering of molecules along **B** (Figure S10a). Although the peak intensities and positions were almost the same in the N and N_X_ phases (Figure S10b), ∆*q*_FWHM_ was smaller in the N_X_ phase than in the N phase. After the transition to the SmA_F_ phase, the diffraction peak became sharper, and ∆*q*_FWHM_ was greatly reduced, suggesting an increase in the correlation length. In the SmA_F_ phase, *d* became almost consistent with the molecular length (*L*) due to the formation of the non‐tilted lamellar structure. After transition to the N_TBF_ and HEC phases, the diffraction pattern became a diffuse arc. In such a heliconical structure, the local director is tilted by a certain angle with respect to the helical axis and continuously rotates around it. As a result, even when the average orientation of the helical axis is aligned, the local orientations are distributed over a conical surface in real space. This angular distribution leads to diffuse arc‐shaped scattering in reciprocal space rather than a sharp diffraction spot. Within the HEC phase temperature range, *d* gradually decreased upon cooling, meaning that the molecules in the lamellar were tilted with respect to the layer normal. The molecular tilting started at the lower‐temperature side of the SmA_F_ phase, with the tilt angle measured as 2° < θ_tilt_ < 7° where $\theta_{\mathrm{tilt}}=\arccos\left(\frac{d}{L}\right)$. Peak intensity, *d* and ∆*q*_FWHM_ jump at the HEC–${\mathrm{SmC}}_{P}^{H}$ phase transition, suggesting the first‐order type. In the ${\mathrm{SmC}}_{P}^{H}$ phase, the molecular orientation with respect to the magnetic field deviated substantially. The ${\mathrm{SmC}}_{P}^{H}$ phase exhibited further gradual reduction in *d*, leading to a maximum tilt angle θ_tilt_ = 21°, despite an almost constant ∆*q*_FWHM_. On the other hand, in the HEC phase, ∆*q*_FWHM_ increased and then reached a plateau. This suggests that, although both the HEC and ${\mathrm{SmC}}_{P}^{H}$ phases possess polar heliconical ordering of the tilted smectics, the coherence of the structure in the HEC phase was reduced upon cooling, implying the generation of small fragments or clusters of the smectic ordering.

### 2.3 Polar Behavior

Next, the polar behavior of various phases was evaluated using BDS, PRC, and SHG measurements.

**FIGURE 4:**
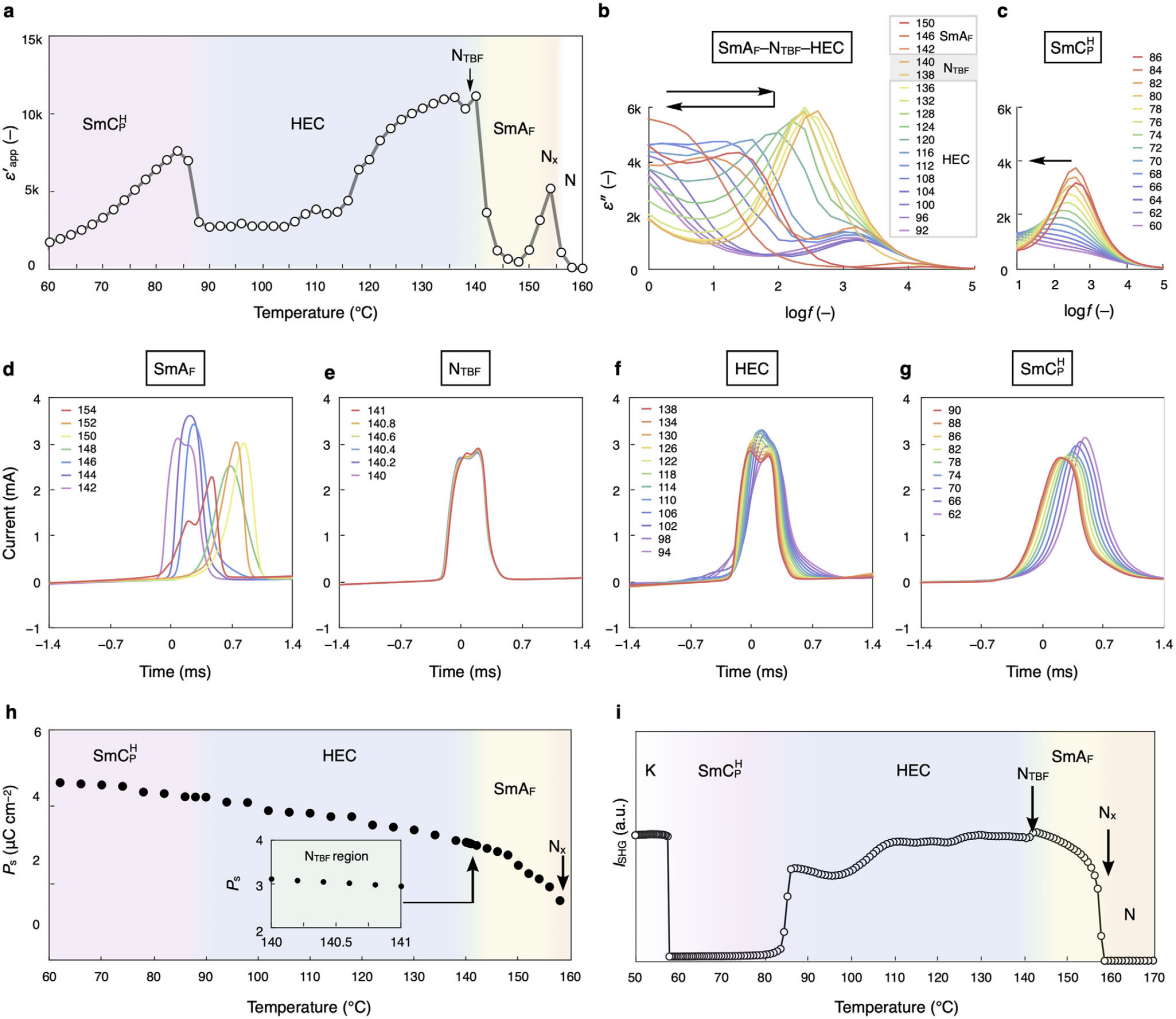
Polar behavior of **Compound 1**. (a) Apparent relative dielectric permittivity ($\varepsilon_{app}^{\prime }$) versus temperature. Dielectric loss (ε′′) vs frequency for the (b) SmA_F_–HEC phase transition and (c) in the ${\mathrm{SmC}}_{P}^{H}$ phase. Black arrows indicate direction of peak shift. PRC profiles of the (d) SmA_F_, (e) N_TBF_, (f) HEC, and (g) ${\mathrm{SmC}}_{P}^{H}$ phases. Temperature‐dependent (h) spontaneous polarization *P*_s_, and (i) SHG.

As shown in Figure 4a–c; Figures S13 and S14, the BDS results revealed that $\varepsilon_{\mathrm{app}}^{\prime }$ [27, 28] increased to 5.2 × 10^3^ at the N–N_X_ phase transition point, decreased sharply to a minimum of 444 in the SmA_F_ phase, and recovered to $\varepsilon_{\mathrm{app}}^{\prime }$ of 11 × 10^3^ in the N_TBF_ phase. This sequence of permittivity changes is likely attributed to the suppression of polarization fluctuations [8] and the restoration of a strong collective relaxation mode [16, 29] in the SmA_F_ and N_TBF_ phases, respectively (Figure 4b). In the HEC phase, $\varepsilon_{app}^{\prime }$ remained 10 × 10^3^ in the high‐temperature region but gradually decreased toward the low‐temperature region, reaching a plateau at approximately 2.7 × 10^3^. Upon the phase transition from the HEC phase to the ${\mathrm{SmC}}_{P}^{H}$ phase, the dielectric permittivity recovered to 7.6 × 10^3^ and then gradually decreased to 1.7 × 10^3^. As shown in Figure 4b, the N_F_ and N_TBF_ phases exhibited a dominant low‐frequency dielectric relaxation mode, which can be attributed to the collective polar fluctuations [5, 16, 28]. In the HEC phase, this low‐frequency mode persisted, while additional relaxation featured emerge at higher frequencies upon cooling, indicating the presence of multiple relaxation processes. This behavior was agreement with the previous results for the HEC phase [19]. As confirmed by XRD measurements, the ${\mathrm{SmC}}_{P}^{H}$ phase exhibited a tilted smectic ordering, which was consistent with a Goldstone mode associated with the azimuthal rotation on the tilt cone [30] (Figure 4c). In contrast, the HEC phase showed smectic‐like short‐range correlations, which may be interpreted as cybotactic‐like local ordering, with no evidence for long‐range smectic order [19].

PRC measurements in the SmA_F_, N_TBF_, HEC, and ${\mathrm{SmC}}_{P}^{H}$ phases (Figure 4d–g; Figures S15 and S16) were carefully conducted upon checking the phase transitions by POM. Notably, the phase transition temperatures inevitably differed between BDS and PRC measurements because of the different cell conditions, such as thickness, used in each experiment. The temperature dependence of spontaneous polarization (*P*_s_) was determined using the PRC results (Figure 4h). The N_X_ phase exhibited *P*_s_ = 1.6 µC cm^−2^. In the SmA_F_ phase, *P*_s_ depended on temperature, ranging between 1.9 and 3.0 µC cm^−2^, whereas the N_TBF_ phase exhibited an almost constant *P*_s_ = 3.0 µC cm^−2^. Two distinct current peaks were observed in the entire HEC region—one at the crossing point of the *E*‐field at zero and the other slightly after that. The *E*‐field at low frequency (20 Hz) clearly split the current signal into two peaks, suggesting that the first and second peaks correspond to relaxation and spontaneous polarization reversal, respectively (Figure S17). Thus, the peak splitting does not imply multiple polarization states. In the ${\mathrm{SmC}}_{P}^{H}$ phase, the relaxation contribution is suppressed due to the development of smectic ordering, resulting in a single current peak and a continuous increase of *P*_s_. Within the ${\mathrm{SmC}}_{P}^{H}$ region, *P*_s_ increases gradually with decreasing temperature, reaching values of 4.6 µC cm^−2^ within the investigated temperature range; this large *P*_s_ reflects a well‐developed smectic ordering in the ${\mathrm{SmC}}_{P}^{H}$ phase. In addition, we confirmed that the ${\mathrm{SmC}}_{P}^{H}$ phase exhibited high cycle stability over 1000 switching cycles (Figure S18).

Finally, temperature‐dependent SHG measurements were performed (Figure 4i). Strong SHG signals were detected in all polar phases except the ${\mathrm{SmC}}_{P}^{H}$ region, where SHG signal was strongly suppressed but remained finite. In the ${\mathrm{SmC}}_{P}^{H}$ region, the sample area in the glass cell became cloudy, likely due to the scattering mode, in which the helical axis randomly orients, such as cholesteric LCs. Consequently, the incident beam was scattered, potentially preventing accurate SHG measurements. More details on SHG are provided in Section 2.5.

### 2.4 Color Modulation of Heliconical Smectic Superstructure

**FIGURE 5:**
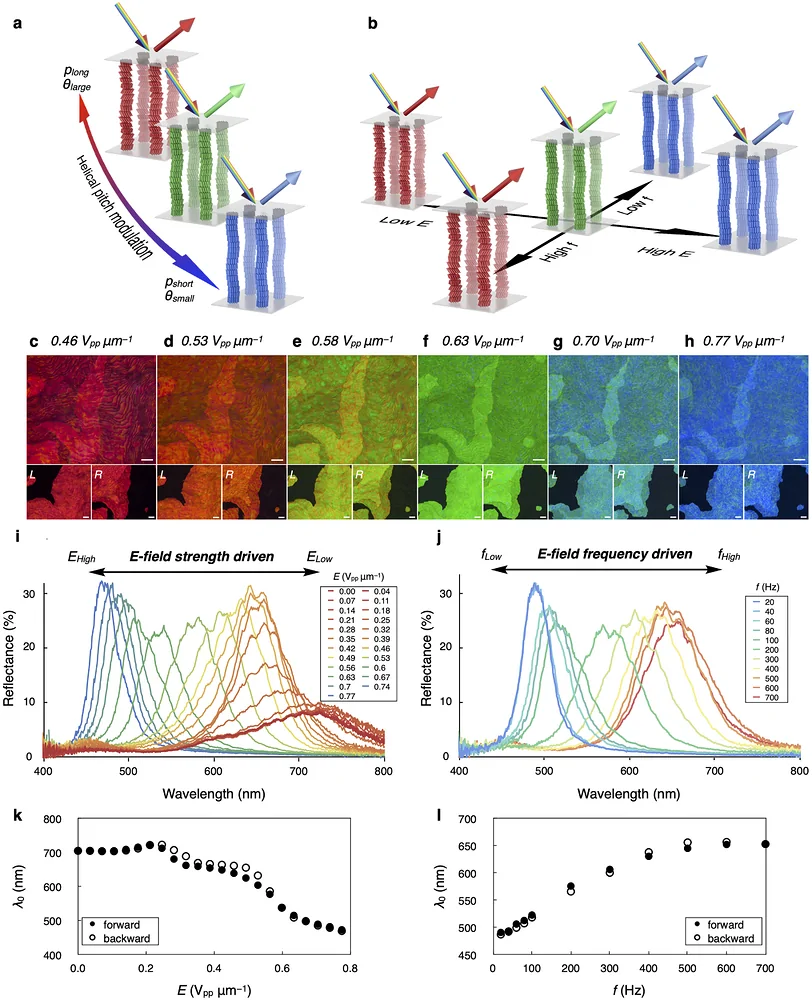
Color modulation in the ${\mathrm{SmC}}_{P}^{H}$ phase of **Compound 1** (*T* = 75°C). Schematic illustrations of (a) modulation of the reflected color associated with the helical pitch and (b) color modulation driven by the *E*‐field strength and frequency. (c–h) Evolution of reflected color in reflection‐mode POM (RPOM) images taken after the pretreatment process. The bottom images were taken with left‐ and right‐circular polarizers (symbol: L and R). Scale bar: 200 µm. Evolution of reflection spectra by modulation of the (i) *E*‐field strength and (j) frequency. Central wavelength (*λ*_0_) vs (k) *E*‐field strength and (l) *E*‐field frequency.

The ${\mathrm{SmC}}_{P}^{H}$ phase initially exhibited a strongly turbid state, yet it demonstrated the faint selective reflection color due to scattering. This turbidity may have been caused by the random orientation of the helical axes. Similarly to the heliconical phases, including the N_TBF_ phase, the molecules in the ${\mathrm{SmC}}_{P}^{H}$ phase were tilted at an angle 0 < θ < π/2 with respect to the helical axis (= layer normal) (Figure 1d,e). Since in the previous heliconical phases, applying the *E*‐field along the helical axis modulates the helical pitch (*p*) and reflective color according to the continuous change in *θ*, without reorienting the helical axis (Figure 5a). Thus, it is natural to expect the field tunable modulation of the reflected color through the pitch modulation also in the present ${\mathrm{SmC}}_{P}^{H}$ phase.

Here, an out‐of‐plane *E*‐field was applied to the cell to examine the influence of the *E*‐field on the ${\mathrm{SmC}}_{P}^{H}$ structure. The application of either DC or rectangular AC *E*‐field (100 Hz) perpendicular to the electrodes deformed the initial multi domains. When the *E*‐field was applied, the helical axis gradually tilted upward along the direction of *E*‐field, concurrently with the progressive formation of enantiomeric domains. (Figure S19). And then, by gently reducing the *E*‐field strength, large domains grew and eventually exhibited vivid red reflection at 0 V. We refer this process to as the pretreatment process. Although the texture still showed slight nonuniformity and light scattering even after this process, a vivid selective reflection originating from Bragg reflection of the heliconical structure was observed, accompanied by a minimal birefringent background. This state is attributed to a homeotropically aligned ${\mathrm{SmC}}_{P}^{H}$, in which the heliconical axis and the layer normal were parallel to the applied *E*‐field. After this pretreatment, when an AC field was applied, the reflected color shifted from red to orange, yellow, green, and blue as gradually increasing the AC *E*‐field and vice versa as decreasing the AC *E*‐field (Figure 5c–h). When these domains were observed through a left‐ or right‐handed circular polarizer (LCP or RCP), a clear and reversible contrast of bright and dark domains was observed, confirming the chirality in each domain (Figures 5c–h and S20). Interestingly, the boundary regions between the domains with opposite chirality showed almost identical reflections both for LCP and RCP, while the crossed‐Nicol images exhibited extremely low transmittance (Figure S21). This operates on the same principle of hyper‐reflective cholesteric LCs [31, 32], which indicates that the left‐ and right‐handed structures were superimposed in this region. Note that such an overlapped region was not observed in thinner cells (4.5 µm) (Figure S22). In the case of the DC *E*‐field, the reflected color changed only slightly from red to reddish orange even under a sufficiently high *E*‐field. The difference between DC and AC driving may partially arise from interfacial charge redistribution under static DC bias (Note S2; Figures S23 and S24), which can reduce the effective internal *E*‐field and thereby limit the achievable pitch modulation.

Further, we investigated the spectroscopic properties and their response to the rectangular AC *E*‐field at a frequency of 100 Hz (Figure 5i and Video S1). First, the transmission spectrum showed a reflection band in the visible light to the near infrared spectral range (Figure S25). The optical response is determined by the half pitch of the polar heliconical structure, as it is governed by the modulation of the refractive index. As a result, the observed reflection band corresponds to the half‐pitch Bragg reflection. When the *E*‐field was increased, the helical pitch decreased according to the decrease of the tilt angle, which caused blueshift of the reflection band. This electrically tunable reflection is reversible with almost no hysteresis between the forward and backward processes (Figure 5i,k and S26a; Video S2). The required *E*‐field to induce pitch modulation—and thereby tune the reflected color among red, green, and blue—was within the range of 0.46–0.77 V_pp_ µm^−1^ (0.23–0.39 V_rms_ µm^−1^), which was compared with conventional heliconical nematic‐type systems [7, 16, 33, 34, 35, 36, 37, 38, 39] as summarized in Figure S27. The present heliconical smectic system (${\mathrm{SmC}}_{P}^{H}$) achieves comparable pitch tuning under substantially lower *E*‐fields, with the exception of the system reported in Ref. [7]. Specifically, the *E*‐field required to reach the red region is 2.1–6.2 times lower than that reported for heliconical nematic‐type systems, while reductions of 1.9–4.3 times and 1.9–4.1 times are observed for the green and blue regions, respectively. Furthermore, we examined the shift of the reflection band upon varying AC frequency while keeping the strength constant. When the frequency was increased from 20 to 400 Hz at *E* = 0.63 V_pp_ µm^−1^, the selective reflection band redshifted by approximately 150 nm. Then, the shift became saturated at higher frequencies (Figure 5j,l; Video S3). Conversely, decreasing the frequency resulted in blueshift without hysteresis (Figure S26b). Note that the shift showed a linear dependence on the frequency between 20 and 400 Hz in both ascending and descending processes. Thus, the tunable color modulation can be achieved even by changing the frequency (Figure S28; Video S4). In such a way, we demonstrated the straightforward modulation of the helical pitch based on both the strength and frequency of the *E*‐field, which enabled color modulation across the visible light spectral region (Figure 5b). Although the above pitch modulation was optically stable on a timescale longer than the period of the applied AC field, certain fluctuations in color and brightness were observed in the texture under POM. The pattern of the fluctuations propagated radially, or sometimes spirally, or in more complex manners, synchronously with the applied AC *E*‐field (Video S5 and S6), so that we consider that this is attributed to the director rotation around the smectic cone. This dynamic behavior looks to be highly intriguing as a new nonequilibrium phenomenon in the polar fluid system.

The optical responses in the present system are more complex than those in conventional heliconical nematic systems, where the competition between elastic and electric‐field‐induced torques results in a linear dependence of the reflection wavelength on the reciprocal *E*‐field strength [34]. As shown in Figure S29, a similar λ∝1/*E* scaling is observed in the low‐field regime (I). However, the intermediate‐field regime (II) exhibits a nonlinear response, while the high‐field regime (III) again shows a linear dependence. Such complex behavior suggests the interplay of multiple competing factors, including torques arising from dielectric and polar couplings [40], the elastic restoring force, and hydrodynamic effects. Although a complete description of the mechanism remains challenging, this continuous pitch change is highly reproducible, thereby ensuring reliable control of the pitch modulation.

The frequency dependence further highlights the fundamentally different driving mechanism in the present system. At a fixed field strength, decreasing the frequency induces blue shift, whereas increasing the frequency leads to red shift, with saturation occurring above approximately 400 Hz (Figure 5j,l). This behavior cannot be explained by simple ionic screening. In general, ionic charge accumulation at the electrodes reduces the effective internal field more strongly at low frequency, which would be expected to suppress pitch modulation and induce red shift. Therefore, the observed blue shift at low frequencies rules out a simple ionic‐screening origin for the AC‐frequency dependence. In contrast, under static DC bias, interfacial charge redistribution can gradually develop and partially screen the effective internal field acting on the ${\mathrm{SmC}}_{P}^{H}$ heliconical structure. Such static screening can limit further pitch modulation under DC driving, whereas AC driving sustains dynamic polarization modulation without forming a persistent static screening state. Therefore, the possible charge‐screening effect under DC bias does not contradict the above discussion regarding the AC‐frequency dependence.

### 2.5 SHG Enhancement on a Heliconical Smectic Superstructure Scaffold

**FIGURE 6:**
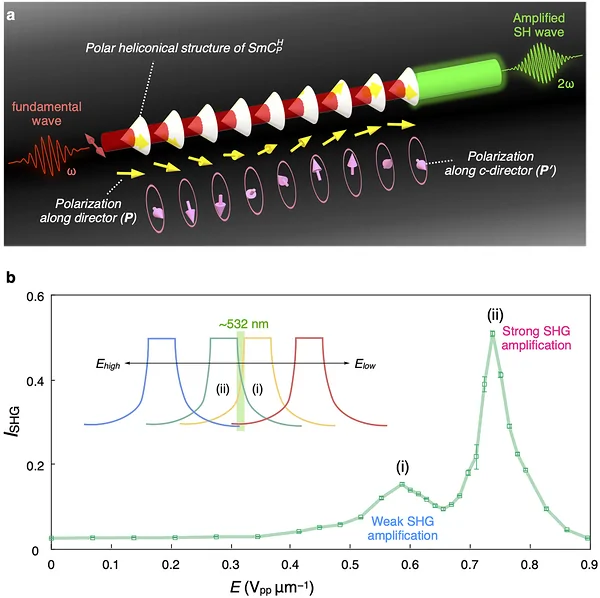
SHG amplification in the ${\mathrm{SmC}}_{P}^{H}$ phase: (a) schematic illustration and (b) SHG intensity vs *E*‐field. The upper inset shows the ideal reflection band modulated by the *E*‐field. The wavelength of the emitted SH light at ≈532 nm matches the band edge on the (i) short‐wavelength or (ii) long‐wavelength side.

In the conventional nonlinear optics, the efficiency of SHG is enhanced by phase matching between the fundamental and harmonic waves [41]. In ferroelectric crystals, quasi‐phase matching is enabled by patterning an alternating polarization structure, leading to obtain efficient SHG [34]. In recent years, SHG enhancement based on the nonclassical phase matching has been successively reported in the N_F_^*^ phase, where the phase matching condition based on photonic bandgap resonance is effectively satisfied, yielding significant SHG amplification [12, 42]. Even in the ${\mathrm{SmC}}_{P}^{H}$ phase, the helicoidal polarization structure with the C_∞_ symmetry may provide the similar optical condition, leading to the nonclassical phase matching to enhance the SHG efficiency (Figure 6a).

We propagated a polarized fundamental beam (Nd:YAG, 1064 nm) along the helical axis normal to the substrate to investigate the SHG response under the helical pitch modulation induced by the *E*‐field. As expected, the SHG signal was enhanced at *E* = 0.59 V_pp_ µm^−1^, and more interestingly, significantly amplified at *E* = 0.74 V_pp_ µm^−1^ (Figure 6b). By comparing the reflectance spectra at these voltages, we found that the short‐wavelength skirt of the former and the long‐wavelength skirt of the latter overlap around the SHG wavelength (532 nm). This suggests that the SHG enhancement originates from phase matching [43] coupled with the short‐ and long‐wavelength band‐edge modes at *E* = 0.74 (band (ii) in Figure 6b) and 0.59 V_pp_ µm^−1^ (band (i) in Figure 6b), respectively. It is well known that, for light propagation in the helical structure of the conventional N* phase, the long‐ and short‐wavelength band edges correspond to the extraordinary and ordinary propagation modes, respectively. In the ${\mathrm{SmC}}_{P}^{H}$ case, the nonlinear polarization in the *x*–*y* plane is nearly parallel to the *c*‐director and can therefore couple with the extraordinary mode, leading to a more pronounced SHG enhancement (Figure 6a). Even then, enhancement also occurs weakly at the short‐wavelength band edge, corresponding to the ordinary mode, due to the contribution from the slight off‐axis component of the nonlinear polarization. Such band‐edge enhancement was also predicted by optical simulation [44]. This nonclassical phase‐matching behavior in the ${\mathrm{SmC}}_{P}^{H}$ phase provides distinct and tunable SHG enhancement that can be achieved using a low *E*‐field applied along the helical axis, unlike in the N_F_^*^ phase [12, 42]. These findings offer a design guideline for tunable and efficient nonlinear optical elements.

### 2.6 Comparison between the Conventional Heliconical Nematic and the Present Polar Heliconical Smectic Systems

In the final section, we contrast heliconical nematic systems with the polar heliconical smectic system (${\mathrm{SmC}}_{P}^{H}$) proposed in this work. The key properties of these systems are summarized from multiple perspectives in Figure S30. Importantly, this comparison shifts the focus from phase classification to material‐specific functionality, emphasizing that the key advance lies not in the phase designation itself but in the emergence of a distinct electro‐optic response unique to this material.

The most distinctive feature of the present material is its ability to achieve continuous and reversible pitch modulation over a wide spectral range under an ultra‐low electric field (approximately one‐third of that required for heliconical nematics [39]). Second, this functionality is realized in a single‐component system and exhibits excellent reproducibility, in contrast to known heliconical nematic systems that rely on multicomponent mixtures or dopants. Third, the absence of alignment layers suggests that the polar smectic nature of the phase may contribute to stabilizing the macroscopic orientation. In particular, achieving uniform homeotropic alignment remains challenging in the N_TBF_ phase. In addition, the polar nature of the system enables access to even‐order nonlinear optical processes. Notably, the helielectric structure provides favorable phase‐matching conditions, resulting in enhanced second‐harmonic generation, as demonstrated above. Combined with its single‐component nature, high optical quality, and straightforward control of the helical axis, this system offers a simple and robust platform for photonic applications, including cavity‐less lasing and up‐conversion devices [45].

## 3 Conclusion

In this study, we performed a detailed analysis of the SmA_F_–N_TBF_–HEC–${\mathrm{SmC}}_{P}^{H}$ phase transitions in a novel polar molecule (**Compound 1**), demonstrating hierarchical symmetry breaking. **Compound 1** was designed through lateral fluorination of a previously reported molecule (Model I), which exhibited the N_F_–HEC phase transition sequence. The molecular parameters obtained by DFT calculations for Model I and **Compound 1** are summarized in Table S2, showing that this single‐fluorine substitution modifies the molecular dipole moment and its orientation relative to the long molecular axis. However, the effect of fluorination on polar LC phases should not be interpreted solely in terms of changes in polar properties, because fluorination may also influence local electrostatic interactions, conformations, and molecular packing and ordering [46, 47, 48]. Thus, fluorination is considered as one of the sensitive factors responsible for the alteration of the phase sequence (SmA_F_–N_TBF_–HEC–${\mathrm{SmC}}_{P}^{H}$ in the present case), rather than as the sole or direct origin of the phase behavior. Future theoretical frameworks may further rationalize this structure–phase relationship [46, 47, 48, 49].

During the phase transition from HEC to ${\mathrm{SmC}}_{P}^{H}$, a distinct first‐order phase transition was observed, accompanied by characteristic changes in POM textures, as well as discontinuous behaviors in BDS, PRC, and SHG, all of which clearly demonstrate the structural transition. Furthermore, *E*‐field‐driven pitch modulation in the ${\mathrm{SmC}}_{P}^{H}$ phase reveals behavior consistent with a polarization‐coupled dynamic mode operating in the subkilohertz regime. Whereas inverse scaling of the reflection wavelength with *E*‐field is observed in the low‐ and high‐voltage regimes, the intermediate regime exhibits deviations from simple scaling, together with reduced reflectance, indicating nonlinear polarization coupling and increased spatial heterogeneity. The combined observation of scaling behavior, nonlinear deviation, and a characteristic frequency cutoff of 400 Hz distinguishes this system from previously reported heliconical materials governed by a frequency‐dependent dielectric–elastic balance at high frequencies [38, 50]. These findings suggest that dynamic polarization governs the electro‐optic functionality of the ${\mathrm{SmC}}_{P}^{H}$ phase.

Taken together, this work not only advances the understanding of hierarchical polar order in soft matter but also demonstrates a material‐specific, low‐frequency and ultralow‐voltage route to electrically tunable structural color in smectic heliconical systems, offering new design principles for polar soft‐matter photonics.

## Author Contributions

**H. N**. and **F. A**. conceived the project and designed the experiments. **H. N**. designed molecules and performed DFT calculation. **A. N**. synthesized and characterized all compounds. **H. N**. carried out DSC, POM, BDS, PRC, and UV–vis‐NIR spectra studies. **H. Y**. constructed reflection spectra measurement system and recorded spectra. **D. K**. performed SHG measurements. **K. S**. and **H. N**. took XRD measurements. **H. N**. and **F. A**. analyzed data and discussed the results. **H. N**. and **F. A**. wrote the manuscript and all authors approved the final manuscript.

## Funding

JSPS KAKENHI (JP22K14594; H. N., JP23K26573; H. Y., JP21H01801, JP23K17341; F. A.), RIKEN Special Postdoctoral Researchers (SPDR) fellowship (H. N.), RIKEN Incentive Research Projects (FY2024: H. N.), JST CREST (JPMJCR23O1; F. A.), and JST SICORP EIG CONCERT‐Japan (JPMJSC22C3; F. A.).

## Conflicts of Interest

The authors declare no conflicts of interest.

## Supporting information




**Supporting File 1**: adma74025‐sup‐0001‐SuppMat.pdf.

**Supporting File 2**: adma74025‐sup‐0002‐VideoS1‐S6.zip.

**Supporting File 3**: adma74025‐sup‐0003‐SpectraData.pdf.

## Data Availability

The authors declare that the data supporting the findings of this study are available within the paper and its Supporting Information files. All other information is available from the corresponding authors upon reasonable request.
