# Supplementary material for: Electrically Tunable Heliconical Smectic Superstructure in Polar Fluids
Source: Adv Mater. 2026 Jul 9;38(53):e74025. doi: 10.1002/adma.74025 (PMC13591660; doi:10.1002/adma.74025)
Supplement: Supplementary file 1 [file ADMA-38-e74025-s001.pdf]

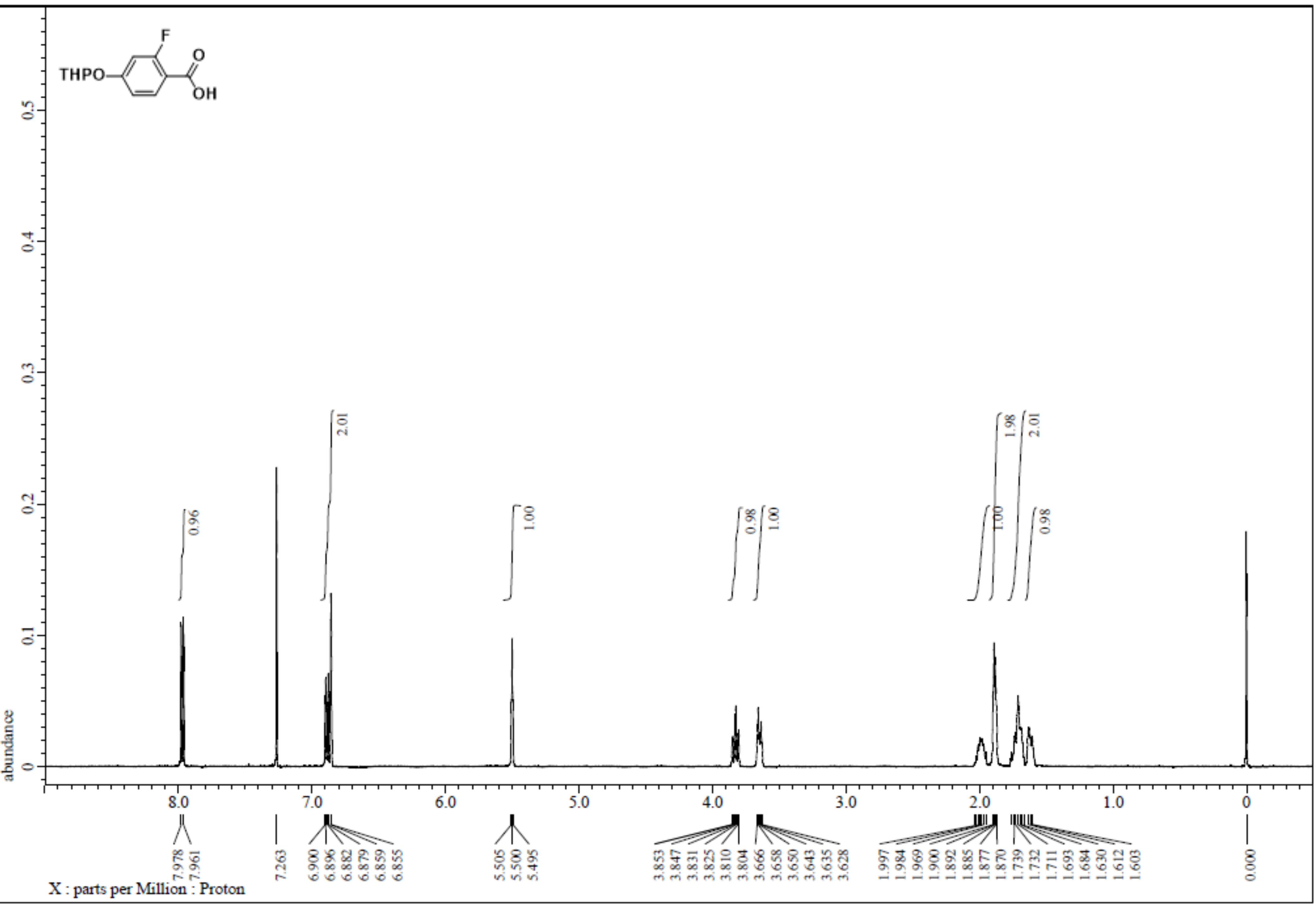

F
O
THPO
OH
0.96
2.01
1.00
0.98
1.00
1.00
1.98
2.01
0.98
abundance
0
0.1
0.2
0.3
0.4
0.5
8.0
7.0
6.0
5.0
4.0
3.0
2.0
1.0
0
7.978
7.961
7.263
6.900
6.896
6.882
6.879
6.859
6.855
5.505
5.500
5.495
3.853
3.847
3.831
3.825
3.810
3.804
3.666
3.658
3.650
3.643
3.635
3.628
1.997
1.984
1.969
1.900
1.892
1.885
1.877
1.870
1.739
1.732
1.711
1.693
1.684
1.630
1.612
1.603
0.000
X : parts per Million : Proton

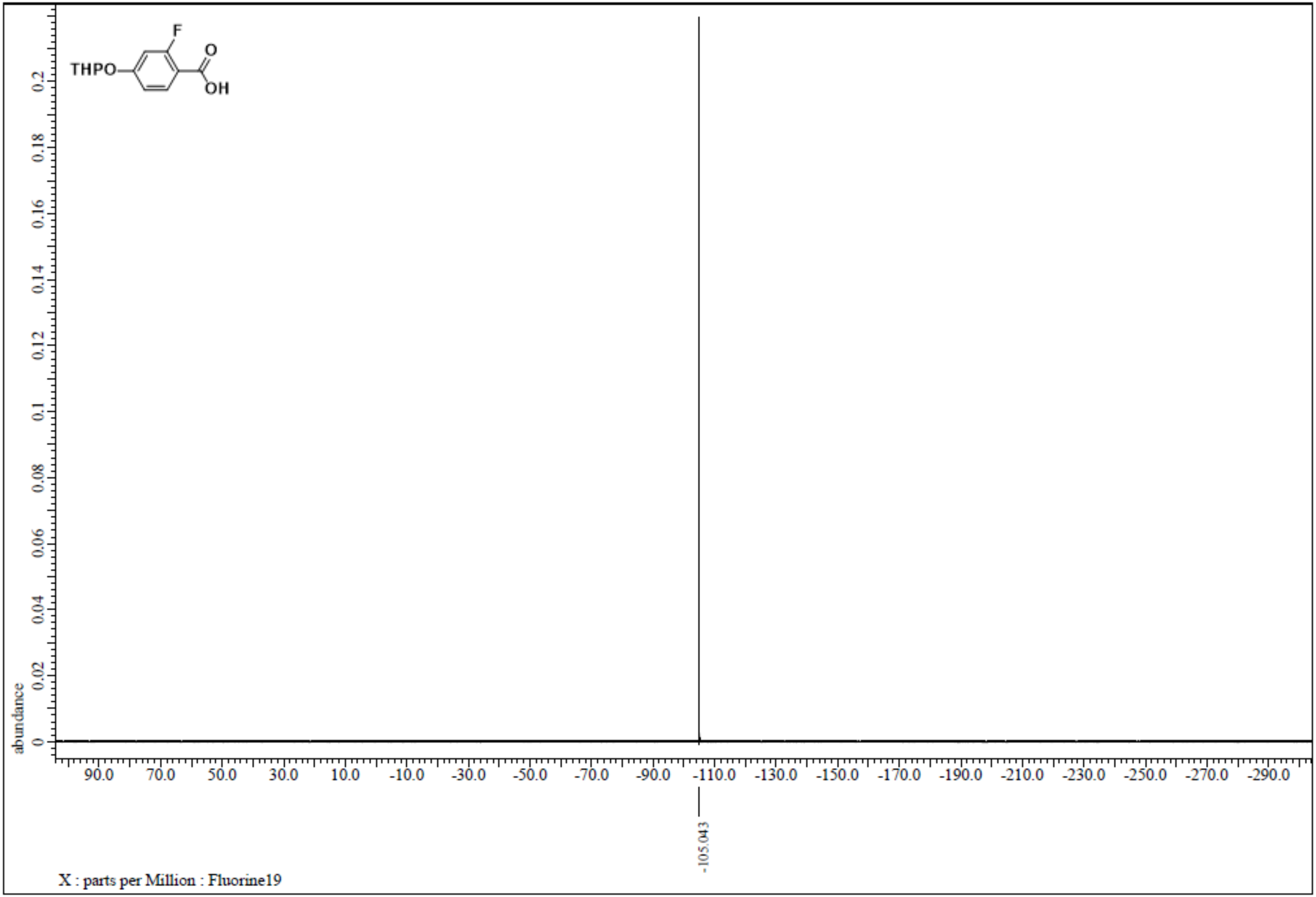
THPO
F
O
OH
abundance
0.2
0.18
0.16
0.14
0.12
0.1
0.08
0.06
0.04
0.02
0
90.0
70.0
50.0
30.0
10.0
-10.0
-30.0
-50.0
-70.0
-90.0
-110.0
-130.0
-150.0
-170.0
-190.0
-210.0
-230.0
-250.0
-270.0
-290.0
-105.043
X : parts per Million : Fluorine19

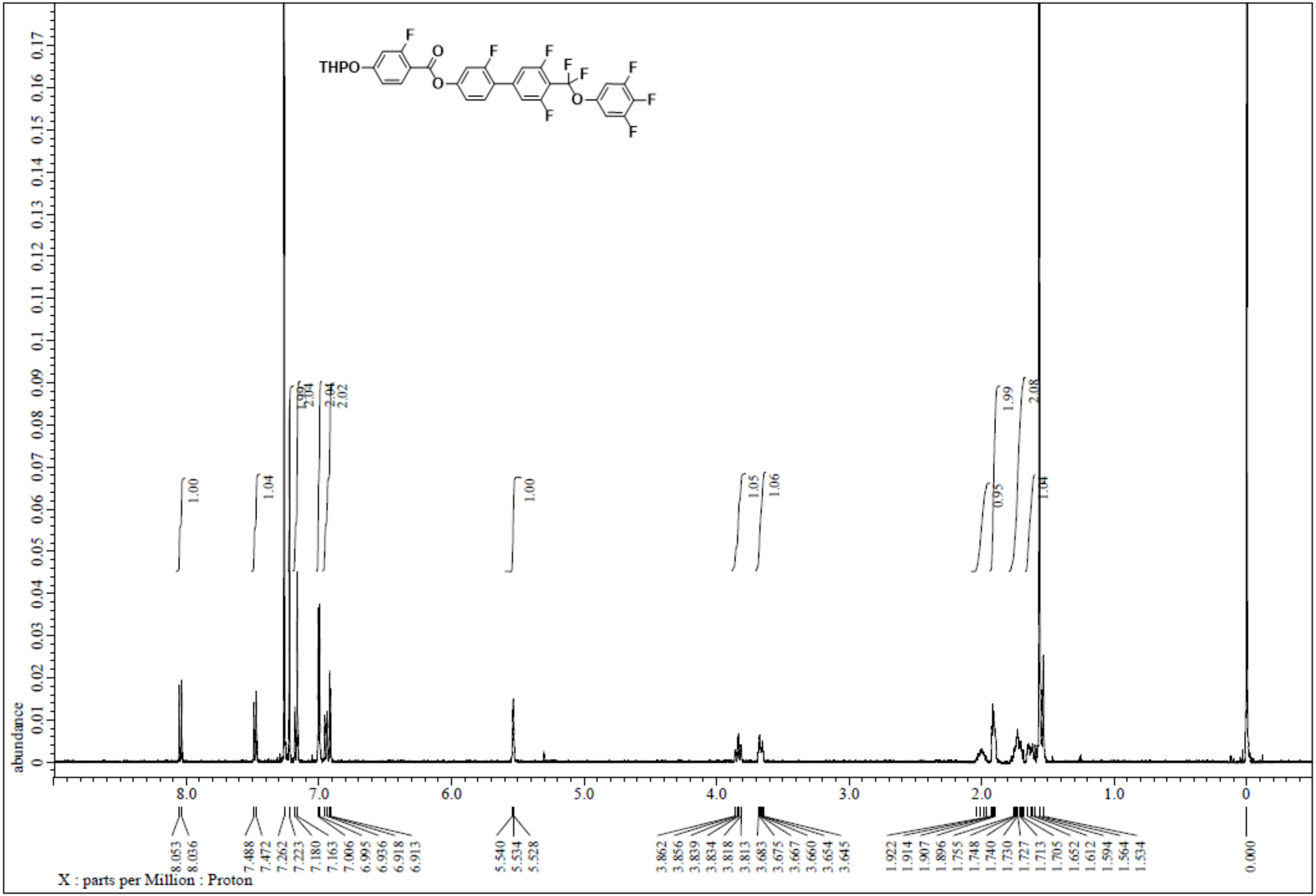
THPO
F
O
F
F
F
F
O
O
F
F
F
F
abundance
0
0.01
0.02
0.03
0.04
0.05
0.06
0.07
0.08
0.09
0.1
0.11
0.12
0.13
0.14
0.15
0.16
0.17
1.00
1.04
1.99
2.04
2.04
2.02
1.00
1.05
1.06
0.95
1.99
2.08
1.04
8.0
7.0
6.0
5.0
4.0
3.0
2.0
1.0
0
8.053
8.036
7.488
7.472
7.262
7.223
7.180
7.163
7.006
6.995
6.936
6.918
6.913
5.540
5.534
5.528
3.862
3.856
3.839
3.834
3.818
3.813
3.683
3.675
3.667
3.660
3.654
3.645
1.922
1.914
1.907
1.896
1.755
1.748
1.740
1.730
1.727
1.713
1.705
1.652
1.612
1.594
1.564
1.534
0.000
X : parts per Million : Proton

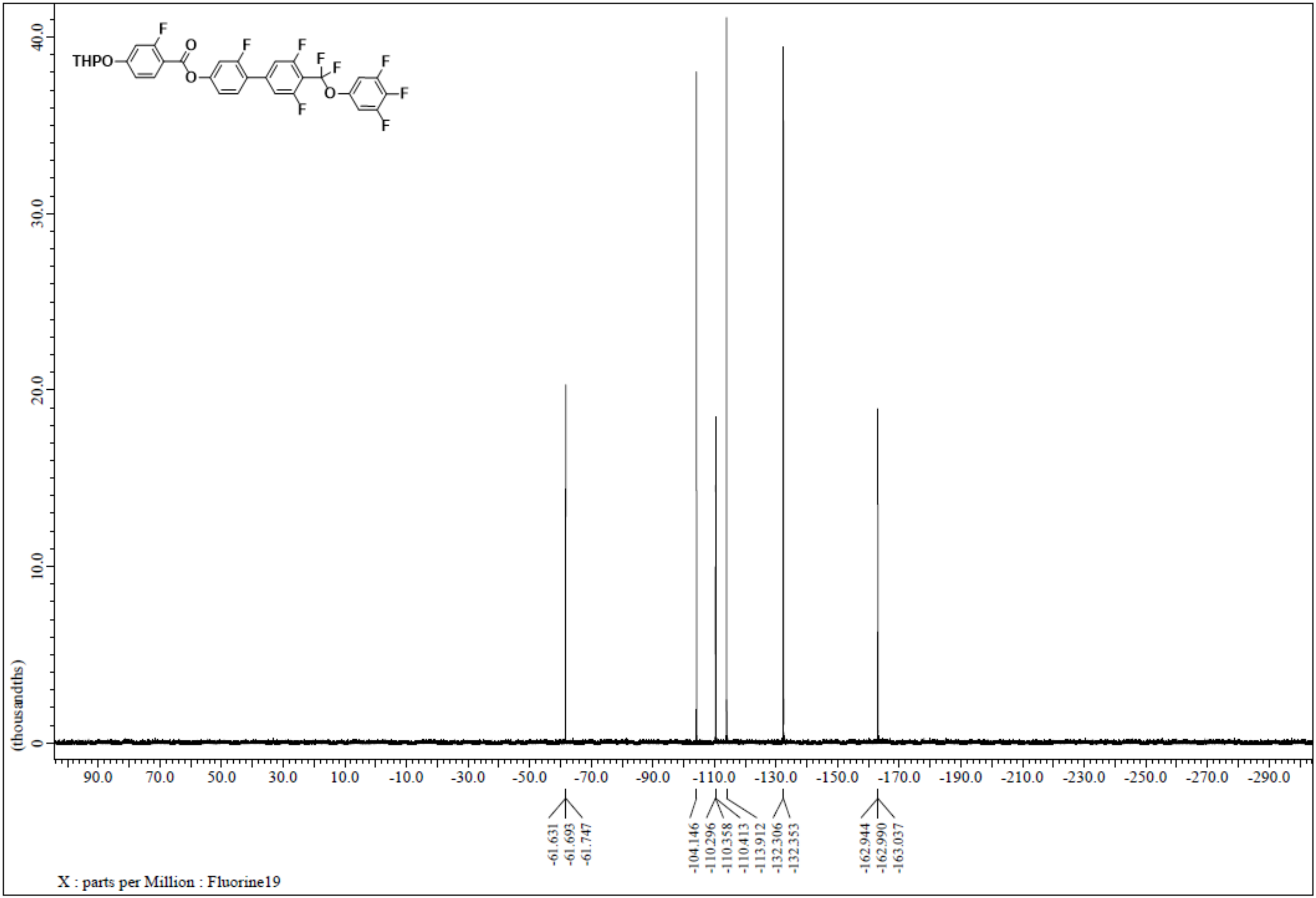
THPO
F
O
F
F
F
F
O
F
F
F
F
40.0
30.0
20.0
10.0
0
(thousandths)
90.0
70.0
50.0
30.0
10.0
-10.0
-30.0
-50.0
-70.0
-90.0
-110.0
-130.0
-150.0
-170.0
-190.0
-210.0
-230.0
-250.0
-270.0
-290.0
-61.631
-61.693
-61.747
-104.146
-110.296
-110.358
-110.413
-113.912
-132.306
-132.353
-162.944
-162.990
-163.037
X : parts per Million : Fluorine19

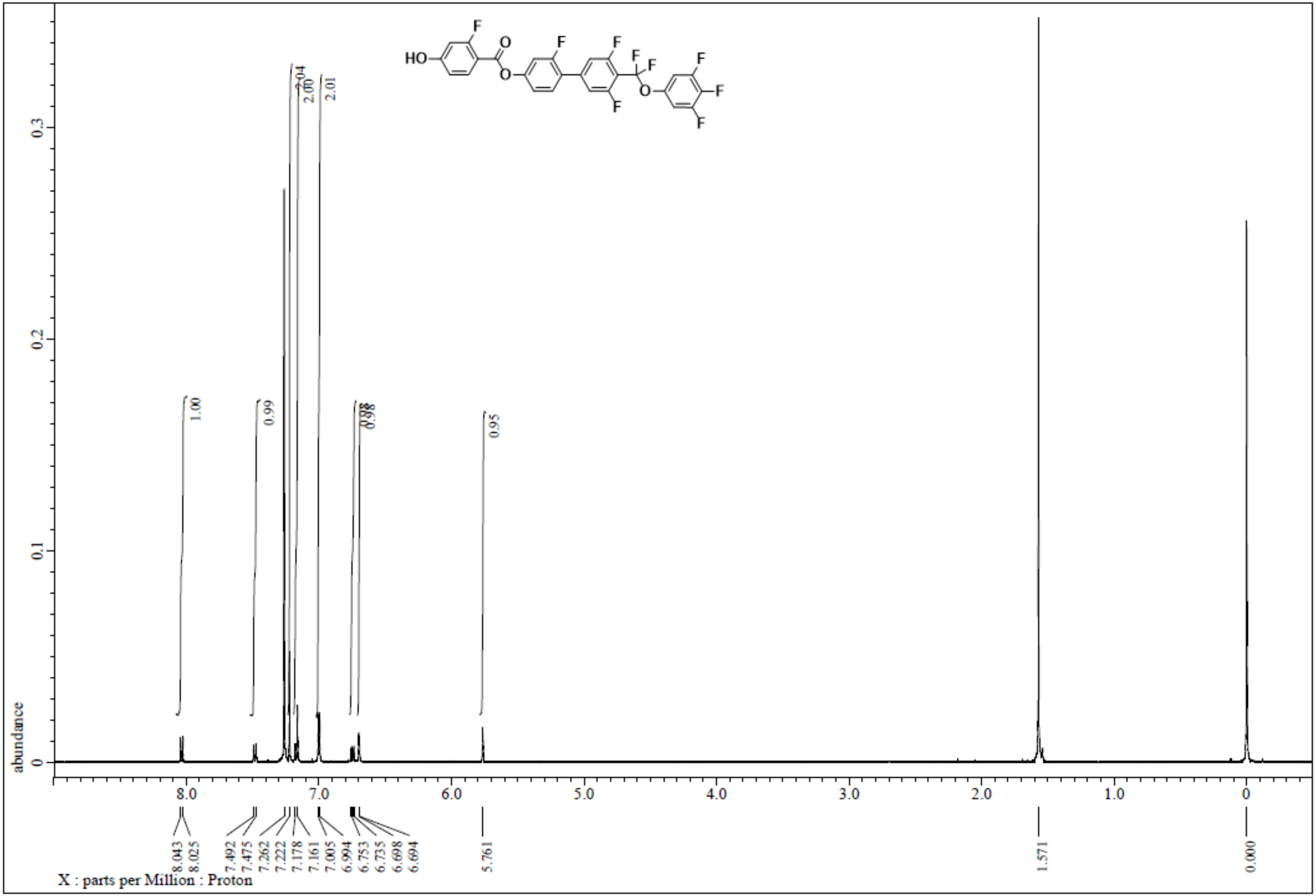

HO
F
O
O
F
F
F
F
O
F
F
F
F
abundance
0.3
0.2
0.1
0
1.00
0.99
2.04
2.00
2.01
0.98
0.98
0.95
8.0
7.0
6.0
5.0
4.0
3.0
2.0
1.0
0
8.043
8.025
7.492
7.475
7.262
7.222
7.178
7.161
7.005
6.994
6.753
6.735
6.698
6.694
5.761
1.571
0.000
X : parts per Million : Proton

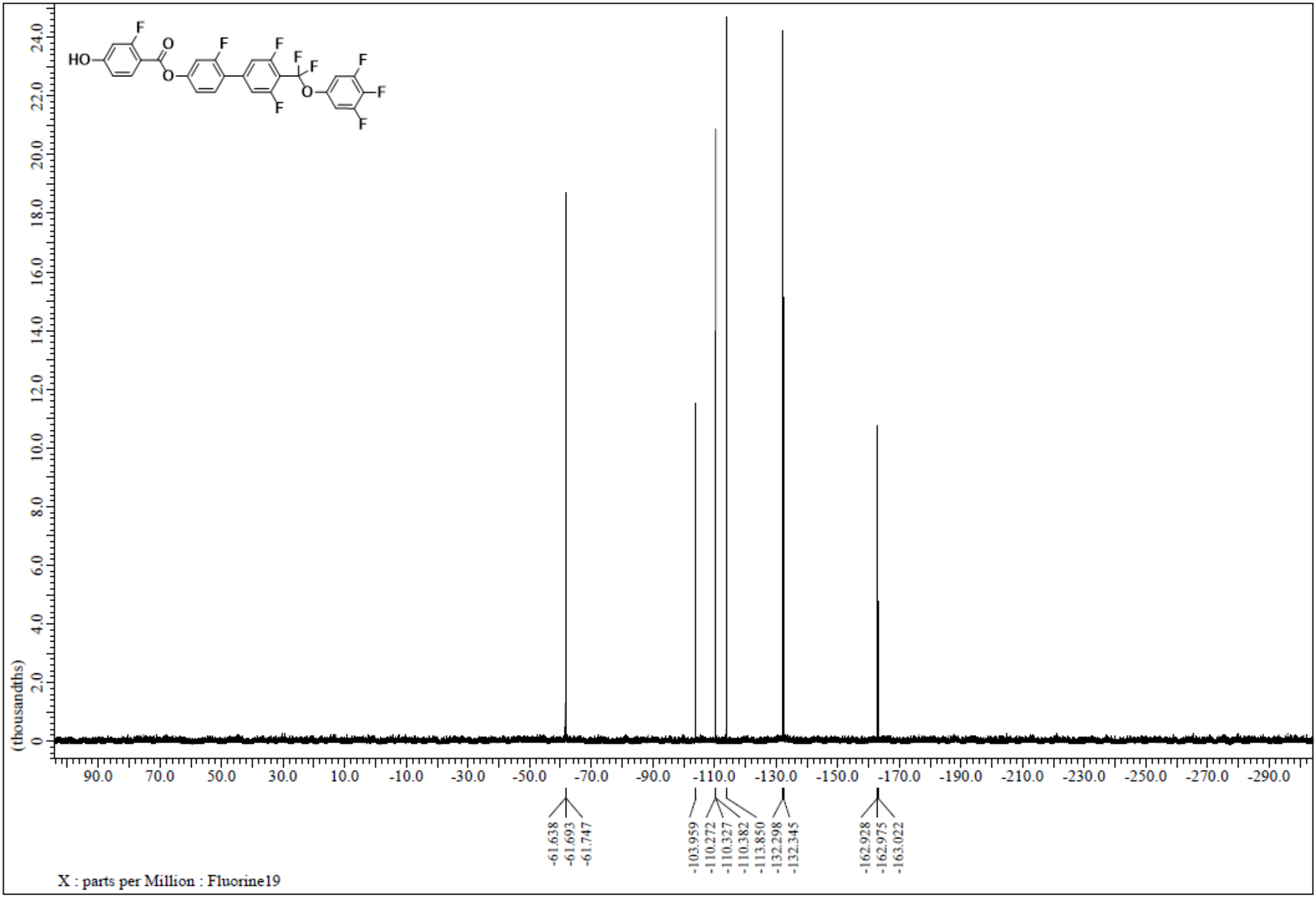

(thousandths)
X : parts per Million : Fluorine19
-61.638
-61.693
-61.747
-103.959
-110.272
-110.327
-110.382
-113.850
-132.298
-132.345
-162.928
-162.975
-163.022
