# Supplementary material for: Electrically Tunable Heliconical Smectic Superstructure in Polar Fluids
Source: Adv Mater. 2026 Jul 9;38(53):e74025. doi: 10.1002/adma.74025 (PMC13591660; doi:10.1002/adma.74025)
Supplement: Supplementary file 2 [file ADMA-38-e74025-s003.pdf]

Supporting Information

Electrically Tunable Heliconical Smectic Superstructure in Polar Fluids

Hiroya Nishikawa*, Dennis Kwaria, Atsuko Nihonyanagi, Koki Sano, Hiroyuki Yoshida and Fumito Araoka*

*To whom correspondence should be addressed.

E-mail: hiroya.nishikawa@riken.jp (H.N.), fumito.araoka@riken.jp (F.A.)

Table of contents

Methods

1. **General and materials** S2
2. **Synthesis and characterization** S4

Supporting Note (Note 1, 2) S7

Supporting Figures (Figures S1–S30) S9

Supporting Tables (Tables S1, S2) S39

Supporting Video (Video S1–S6) S41

Supporting References S42

Methods

1. General and materials

Nuclear magnetic resonance (NMR) spectroscopy: ^{1}H, ^{13}C, and ^{19}F NMR spectra were recorded on JNM-ECZ500 (JEOL) operating at 500 MHz, 126 MHz, and 471 MHz for ^{1}H [^{1}H{^{19}F}], ^{13}C{^{1}H} [^{13}C{^{1}H,^{19}F}] and ^{19}F [^{19}F{^{1}H}] NMR, respectively, using the TMS (trimethylsilane) as an internal standard for ^{1}H NMR and the deuterated solvent for ^{13}C NMR. The absolute values of the coupling constants are given in Hz, regardless of their signs. Signal multiplicities were abbreviated by s (singlet), d (doublet), t (triplet), q (quartet), quint (quintet), sext (sextet), and dd (double–doublet), respectively.

High-resolution mass (HRMS) spectroscopy: The high-resolution electrospray ionization mass spectrometry (HRESI-MS) was performed on SYNAPT G2-S/UPLC system (Waters ltd.).

Density Functional Theory (DFT) Calculation: Calculations were performed using the Chem3D (pro, 22.2.0.3300) and Gaussian 16 (G16, C.01) softwares (installed at the RIKEN Hokusai GreatWave Supercomputing facility) for MM2 and DFT calculations, respectively. GaussView 6 (6.0.16) software was used to visually analyze the calculation results. [S1] Dipole moments of molecules were calculated using b3lyp-gd3bj/6-311+g(d,p) level. The calculation method is as follows: opt=tight b3lyp-gd3bj/6-311+g(d,p) geom=connectivity empiricaldispersion=gd3bj int=ultrafine.

Polarized optical microscopy (POM): Polarized optical microscopy was performed on a polarizing microscope (Eclipse LV100 POL, Nikon) with a hot stage (HSC402, INSTEC) on the rotation stage. Unless otherwise noted, the sample temperature was controlled using the INSTEC temperature controller (mk2000, INSTEC).

Differential scanning calorimetry (DSC). Differential scanning calorimetry was performed on a calorimeter (DSC3+, Mettler-Toledo). Rate: 10 K min^{-1}. Cooling/heating profiles were recorded and analyzed using the Mettler-Toledo STARe software system.

Dielectric spectroscopy. Dielectric relaxation spectroscopy was performed ranging between 1 Hz and 1 MHz using an impedance/gain-phase analyzer (SI 1260, Solartron Metrology) and a dielectric interface (SI 1296, Solartron Metrology). Prior to starting the measurement of the LC sample, the capacitance of the empty cell was determined as a reference. The temperature was controlled using a homemade heater block and temperature controller. The automatic measurement was taken place by the LabVIEW software programming.

PRC measurement. PRC measurements were performed in the temperature range of the polar phase under a triangular-wave *E*-field using a ferroelectricity evaluation system (FCE 10, TOYO Corporation), which is composed of an arbitrary waveform generator (2411B), an IV/QV amplifier (model 6252) and a simultaneous A/D USB device (DT9832).

SHG/SHM measurements. The SHG/SHM investigation was carried out using a Q-switched DPSS Nd: YAG laser (FQS-400-1-Y-1064, Elforlight) at λ = 1064 nm with a 5 ns pulse width (pulse energy: 400 μJ). The primary beam was incident on the LC cell followed by the detection of the SHG signal. The electric field was applied normally to the LC cell.

Wide-angle X-ray diffraction (WAXD) analysis. 2D WAXD measurement was performed using NANOPIX system (Rigaku). The samples held in a homemade holder with magnets [S12] was measured at a constant temperature using a temperature controller and a hot stage (mk2000, INSTEC) with high temperature-resistance samarium cobalt magnets (~1 T, Shimonishi Seisakusho Co., Ltd.). The scattering vector q ($q = 4\pi\sin\theta\ \lambda^{-1}$; 2θ and λ = scattering angle and wavelength of an incident X-ray beam (1.54 Å) and position of an incident X-ray beam on the detector were calibrated using several orders of layer diffractions from silver behenate (d = 58.380 Å). The sample-to-detector distances were 82.20 mm, where acquired scattering 2D images were integrated along the Debye–Scherrer ring by using software (Igor Pro with Nika-plugin), affording the corresponding one-dimensional X-ray diffraction profiles.

Information of used liquid crystalline (LC) cells:

ITO glass cell (EHC):

- ITO-coated type, electrode area: 5 × 10 mm
- Experiments: POM (thickness: 10.0 and 25.0 μm) and DR (thickness: 10.0 μm)

ITO glass cell (homemade):

(case i) ITO-coated type (non-alignment layer), electrode area: 4 × 5 mm

(case i) Experiments: PRC (thickness: 4.6 μm) studies

(case ii) ITO-coated type (silanized layer), electrode area: 4 × 5 mm

(case ii) Experiments: Pitch modulation (thickness: 14.1 μm) studies

Synparallel-rubbed (SP) cell (EHC):

- PI-coated type, thickness: 5.0 μm
- Alignment layer: LX-1400
- Experiments: POM studies

2. Synthesis of Compound 1.

2.1. Synthetic route

All compounds (**1–6**) were synthesized according to previous literature [S2] (Scheme S1). NMR and MS spectra for final target **1** are shown in Figure S28–S31.

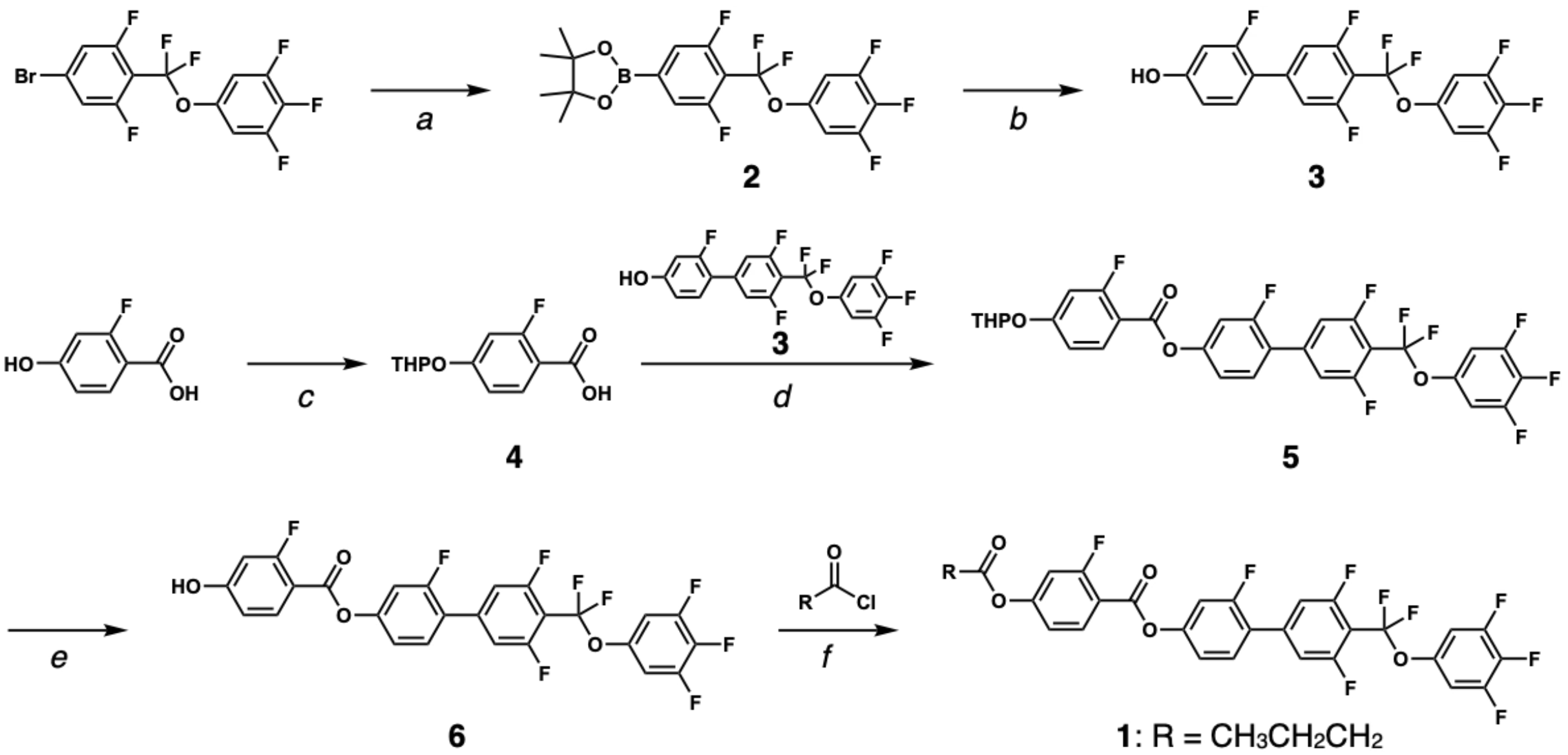

Scheme S1 Synthetic pathway of **Compound 1**. a) B_2pin_2, $Pd(dppf)Cl_2$-CH_2Cl_2, KOAc, 1,4-dioxane, MM400 (30 Hz), 110 °C, 10 min, b) $Pd(OAc)_2$, SPhos, K_2CO_3, THF/H_2O, 55 °C, 3 h, c) 3,4-dihydro-2*H*-pyran, Et_2O, 35 °C, 2.5 h; r.t., 20 h, d) **3**, EDAC-HCl, DMAP, CH_2Cl_2, 0 °C, 1 h; r.t., 1 h, e) *p*-TsOH, CH_2Cl_2/MeOH, 50 °C, 1.5 h, f) TEA, CH_2Cl_2, 0 °C; r.t.

2.2.1. ***Synthesis of 2-fluoro-4-((tetrahydro-2H-pyran-2-yl)oxy)benzoic acid*** **(4)**

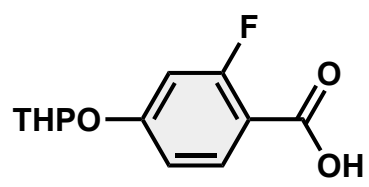

^{1}H{^{19}F}-NMR (500 MHz, $CDCl_3$): δ 7.97 (d, *J* = 8.7 Hz, 1H), 6.90–6.86 (m, 2H), 5.50 (t, *J* = 2.7 Hz, 1H), 3.85–3.80 (m, 1H), 3.65 (dt, *J* = 11.3, 3.9 Hz, 1H), 2.04–1.95 (m, 1H), 1.90–1.87 (m, 2H), 1.77–1.67 (m, 2H), 1.63–1.60 (m, 1H)

^{19}F{^{1}H}-NMR (471 MHz, $CDCl_3$): δ −105.0

2.2.2. ***Synthesis of 4'-(difluoro(3,4,5-trifluorophenoxy)methyl)-2,3',5'-trifluoro-[1,1'-biphenyl]-4-yl 2-fluoro-4-((tetrahydro-2H-pyran-2-yl)oxy)benzoate*** **(5)**

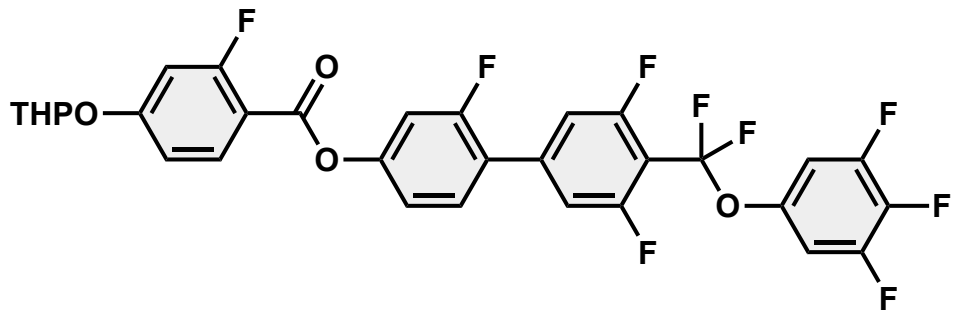

^{1}H{^{19}F}-NMR (500 MHz, $CDCl_3$): δ 8.04 (d, J = 8.7 Hz, 1H), 7.48 (d, *J* = 8.0 Hz, 1H), 7.22 (s, 2H), 7.18–7.16 (m, 2H), 7.00 (d, *J* = 5.6 Hz, 2H), 6.96-6.91 (m, 2H), 5.53 (t, *J* = 3.0 Hz, 1H), 3.86–3.81 (m, 1H), 3.68–3.64 (m, 1H), 2.04–1.96 (m, 1H), 1.92–1.90 (m, 2H), 1.76–1.68 (m, 2H), 1.65–1.61 (m, 1H)

^{19}F{^{1}H}-NMR (471 MHz, $CDCl_3$): δ −61.7 (t, *J* = 27.3 Hz, 2F), −104.1 (s, 1F), −110.4 (t, *J* = 27.6 Hz, 2F), −113.9 (s, 1F), −132.3 (d, *J* = 18.4 Hz, 2F), −163.0 (t, *J* = 21.9 Hz, 1F)

2.2.3. ***Synthesis of 4'-(difluoro(3,4,5-trifluorophenoxy)methyl)-2,3',5'-trifluoro-[1,1'-biphenyl]-4-yl 2-fluoro-4-hydroxybenzoate*** **(6)**

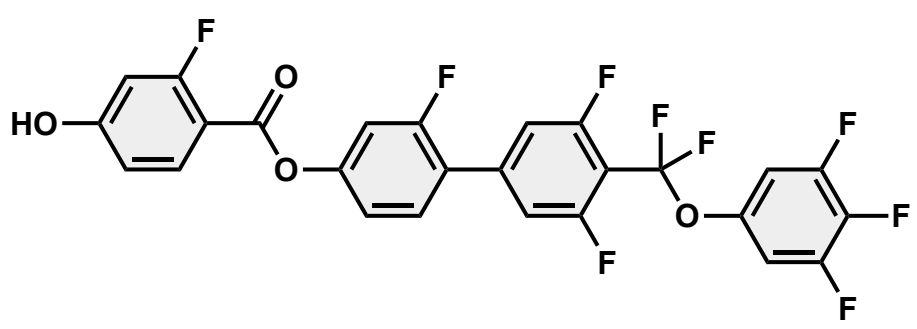

^{1}H{^{19}F}-NMR (500 MHz, $CDCl_3$): δ 8.03 (d, *J* = 8.7 Hz, 1H), 7.48 (d, *J* = 8.6 Hz, 1H), 7.22 (s, 2H), 7.18z–7.16 (m, 2H), 7.00 (d, *J* = 5.7 Hz, 2H), 6.75 (dd, *J* = 8.6, 2.4 Hz, 1H), 6.70 (d, *J* = 2.4 Hz, 1H), 5.76 (s, 1H)

^{19}F{^{1}H}-NMR (471 MHz, $CDCl_3$): δ -61.7 (t, J = 25.7 Hz, 2F), -104.0 (s, 1F), -110.3 (t, J = 25.9 Hz, 2F), −113.9 (s, 1F), −132.3 (d, *J* = 22.1 Hz, 2F), −163.0 (t, *J* = 22.1 Hz, 1F)

2.2.4. ***Synthesis of 4'-(difluoro(3,4,5-trifluorophenoxy)methyl)-2,3',5'-trifluoro-[1,1'-biphenyl]-4-yl 4-(butyryloxy)-2-fluorobenzoate (Compound 1)***

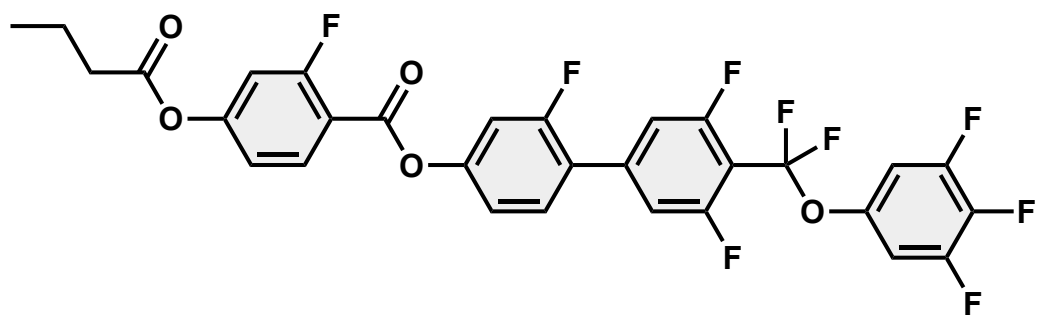

White powder, 84.9% yield

$^{1}H\{^{19}F\}$-NMR (500 MHz, $CDCl_3$): δ 8.14 (dd, *J* = 7.8, 1.5 Hz, 1H), 7.50 (dd, *J* = 7.9, 0.9 Hz, 1H), 7.23 (s, 2H), 7.20–7.18 (m, 2H), 7.10–7.08 (m, 2H), 7.00 (d, *J* = 5.6 Hz, 2H), 2.59 (t, *J* = 7.4 Hz, 2H), 1.81 (td, *J* = 14.8, 7.4 Hz, 2H), 1.07 (t, *J* = 7.4 Hz, 3H)

$^{19}F\{^{1}H\}$-NMR (471 MHz, $CDCl_3$): δ -61.7 (t, *J* = 25.7 Hz, 2F), −103.9 (s, 1F), −110.3 (t, *J* = 25.7 Hz, 2F), −113.6 (s, 1F), −132.3 (d, *J* = 18.4 Hz, 2F) , −163.0 (t, *J* = 21.9 Hz, 1F)

$^{13}C\{^{1}H,^{19}F\}$-NMR (126 MHz, $CDCl_3$): δ 171.0, 162.8, 161.5, 159.9, 159.5, 156.1, 151.9, 151.0, 144.6, 140.7, 138.5, 133.4, 130.6, 123.4, 120.1 (t, *J* = 262.5 Hz), 118.4, 117.8, 114.6, 113.1, 111.2, 110.9, 108.9 (t, *J* = 32.1 Hz), 107.5, 36.1, 18.2, 13.6

ESI-HRMS (m/z): [M+Na^+] calc for 651.0830; found, 651.0834

Note 1 Characteristic of HEC phase

The distinct features of the present "HEC" phase are summarized below:

(1) it exhibits a helielectric conical ordering and appears below the N_{TBF} phase via a continuous transition;

(2) XRD measurements upon magnetic field application indicate a tilted smectic-like layering, but without long-range positional order, in contrast to the N_{TBF} and SmC_P^H phases; and

(3) the SmC_P^H phase emerges from this phase through a distinct first-order phase transition.

Based on these observations, we initially presumed that the HEC phase corresponds to a helielectric conical structure with cybotactic SmC-like clusters, rather than a true smectic phase possessing long-range positional order. However, these findings alone are insufficient to conclusively classify it as a cluster phase. Therefore, we designate it as a helielectric conical phase (HEC phase). This terminology was also adopted in our previous work [S2] with the same rationale, and hence we use the same notation "HEC" here.

Note 2 DC-bias-dependent low-frequency dielectric response evaluated by BDS.

To examine whether mobile-charge-related effects contribute to the different responses under AC/DC driving, we performed DC-bias-dependent broadband dielectric spectroscopy (BDS) measurements using a 14-μm ITO cell. A small AC probing voltage of 100 mV was applied together with DC biases of 0–6 V, allowing the dielectric response of the DC-biased states to be evaluated. The complex dielectric spectra were analyzed using two Havriliak–Negami relaxation contributions together with the conductivity term employed in a previous dielectric study of polar LCs [S13]:

$$\varepsilon^*(f) = \varepsilon_\infty + \sum_{k=1}^{2} \frac{\Delta\varepsilon_k}{\left[1 + \left(j\frac{f}{f_k}\right)^{a_k}\right]^{b_k}} + \left(\frac{\sigma_{DC}}{j2\pi f \varepsilon_0}\right)^N$$

, where ε_∞, $\Delta\varepsilon_k$ and f_k are the high-frequency permittivity, the dielectric strength and relaxation frequency, respectively; a_k and b_k are broadness exponents of the corresponding relaxation mode; σ_{DC} denotes the DC conductivity parameter in the low-frequency term, while N is an exponent with values between 0 and 1. For a consistent comparison among different DC biases, b_k was fixed at 1 and N was treated as a common global parameter for all spectra, yielding $N = 0.897$. Because this fitted value is slightly below unity, the field dependence of this contribution is discussed in terms of a low-frequency charge-related response, rather than as a direct measure of intrinsic bulk DC conductivity. The fitted BDS spectra and the DC-field dependence of the effective conductivity evaluated at 1 Hz from the fitted conductivity term are shown in Figures S23 and S24, respectively. The extracted low-frequency charge-related response under DC bias increased markedly, reached an almost saturated level by 0.286 V μm^{-1}, and subsequently decreased at higher static fields. Importantly, the DC electric fields of 0.286 and 0.357 Vμm^{-1} directly overlap with the AC field range in which pronounced optical pitch modulation was observed, 0.23–0.39 V_{rms} μm^{-1}. Therefore, the low-frequency charge-related response under DC bias is already nearly saturated in the field range where AC driving produces pronounced pitch modulation. Although these results do not directly visualize charge accumulation at the interfaces, they provide frequency-dependent dielectric evidence consistent with the development of interfacial charge redistribution and internal-field screening in the DC-biased state. Such a persistent static screening state can reduce the effective internal DC field acting on the SmC_P^H heliconical structure and thereby limit further pitch modulation under DC driving.

Supporting Figures (Figures S1–S30)

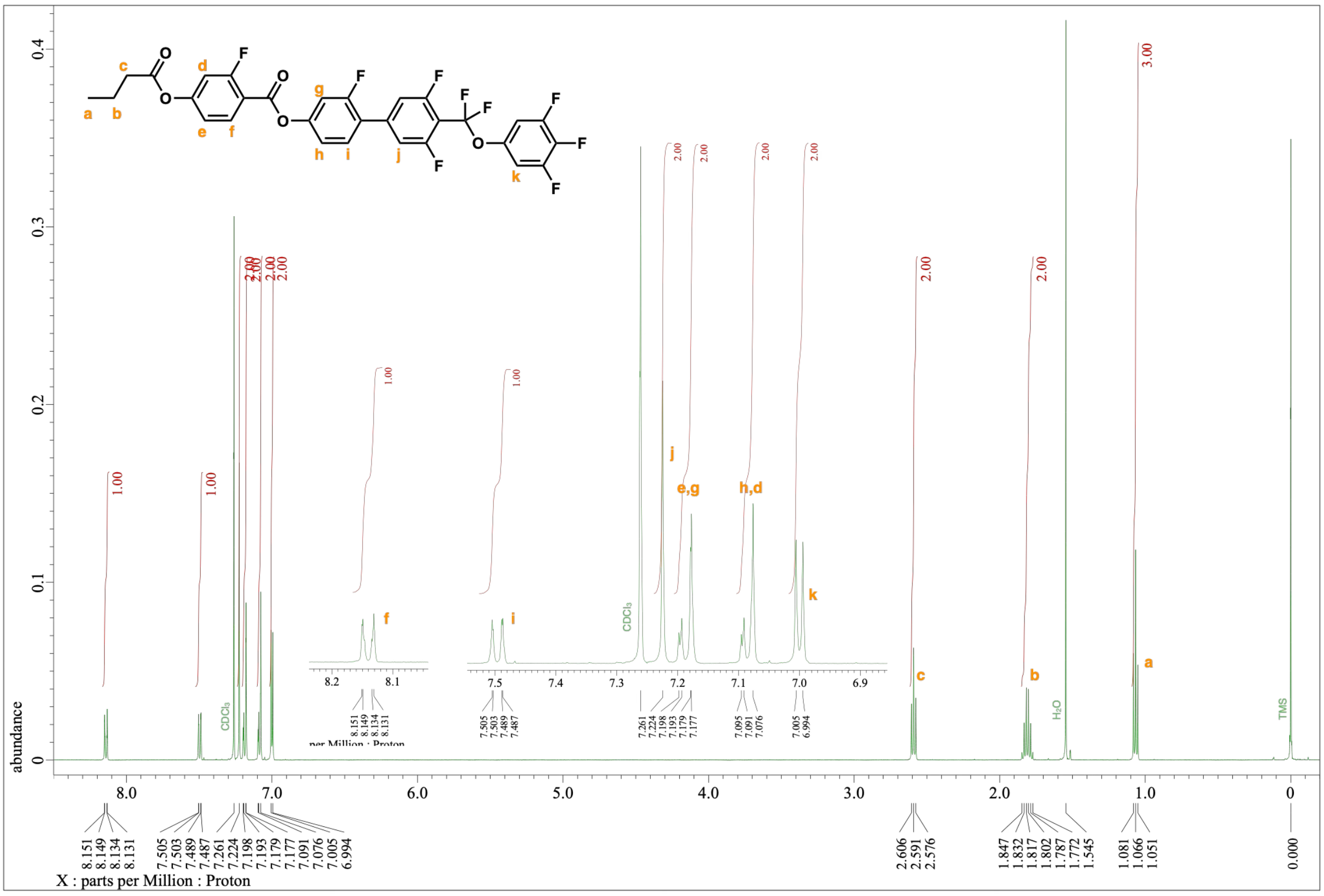

Figure S1 $^{1}H\{^{19}F\}$NMR spectra for **Compound 1**.

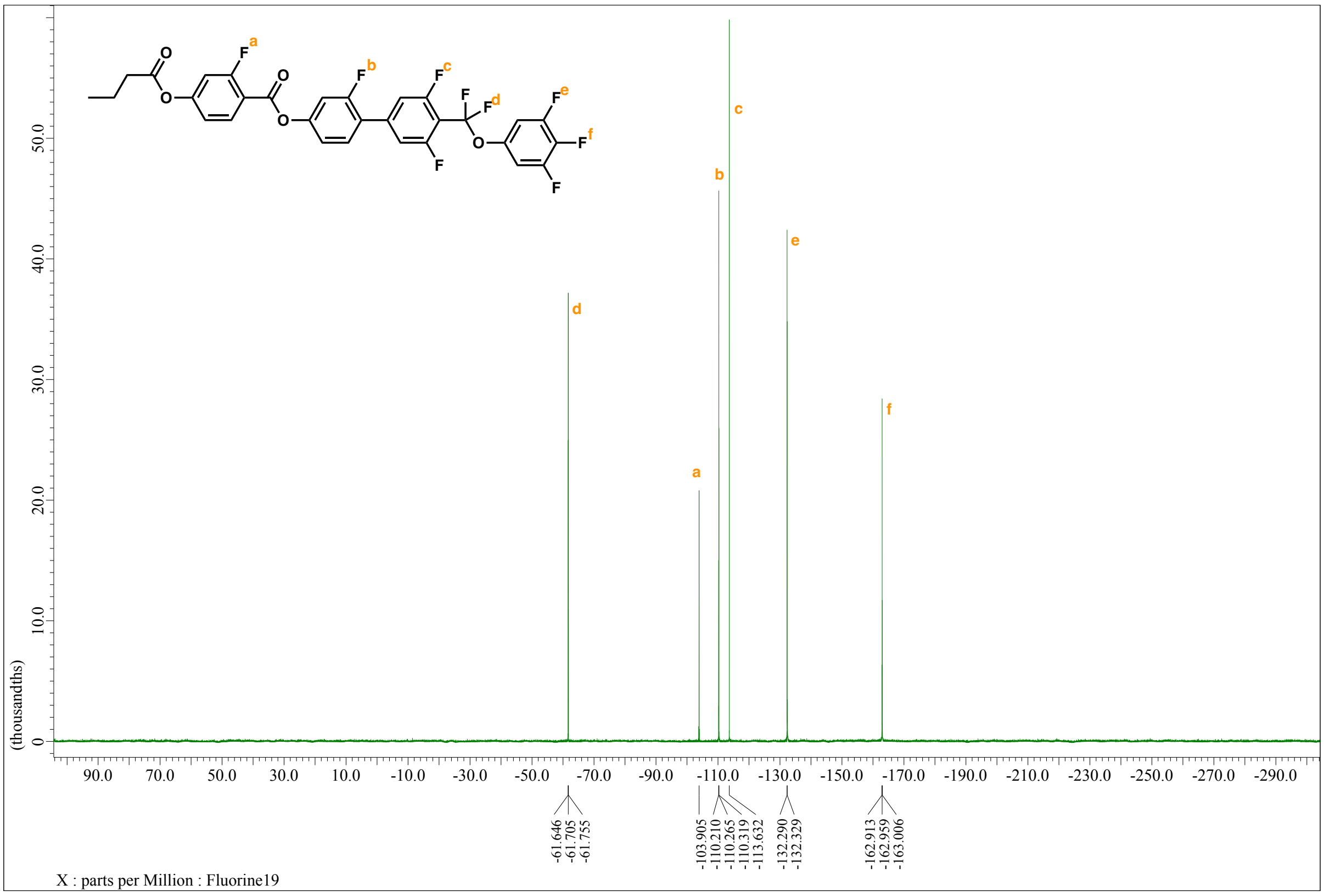

Figure S2 $^{19}F\{^{1}H\}$NMR spectra for **Compound 1**.

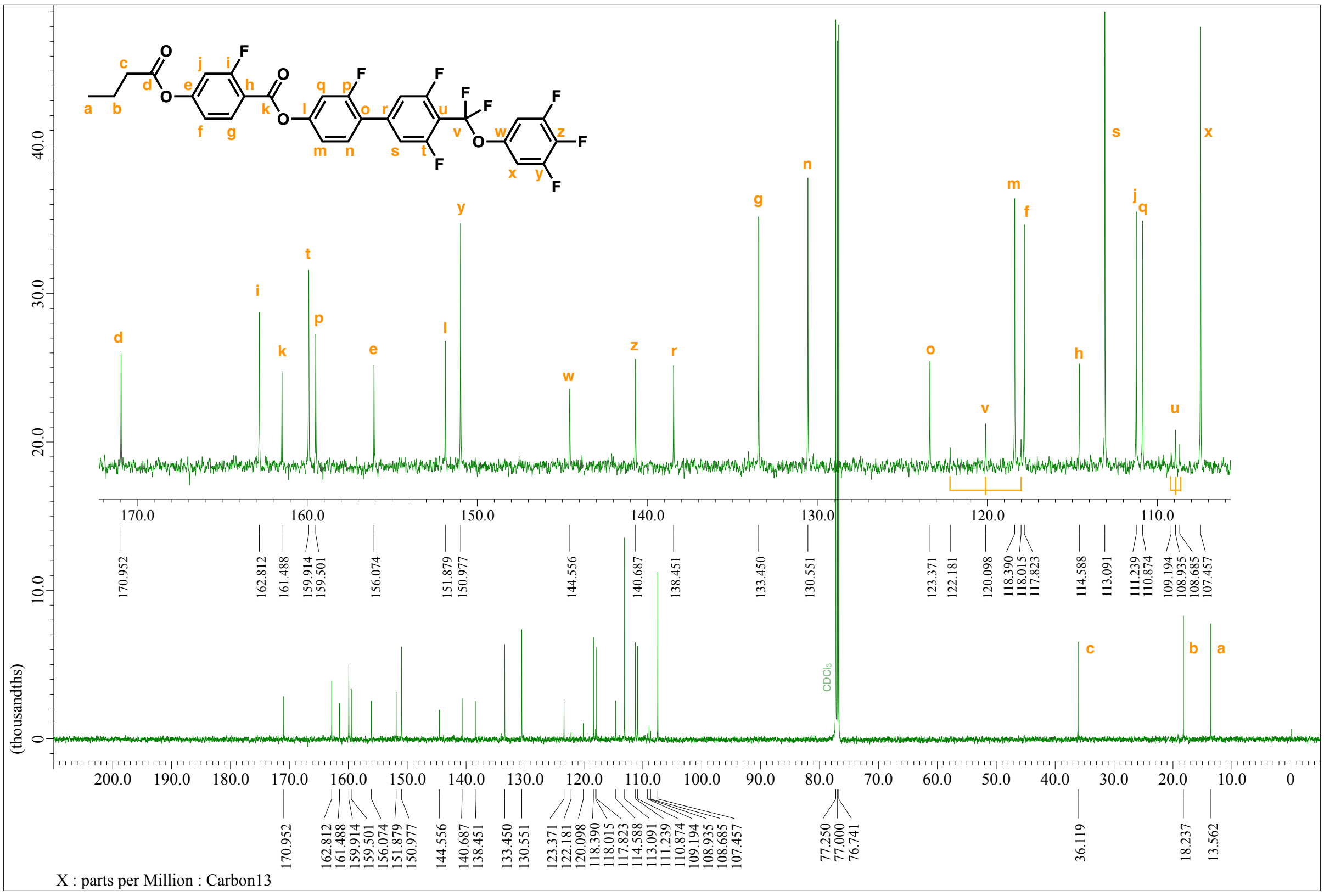

Figure S3 $^{13}C\{^1H,^{19}F\}$NMR spectra for **Compound 1**.

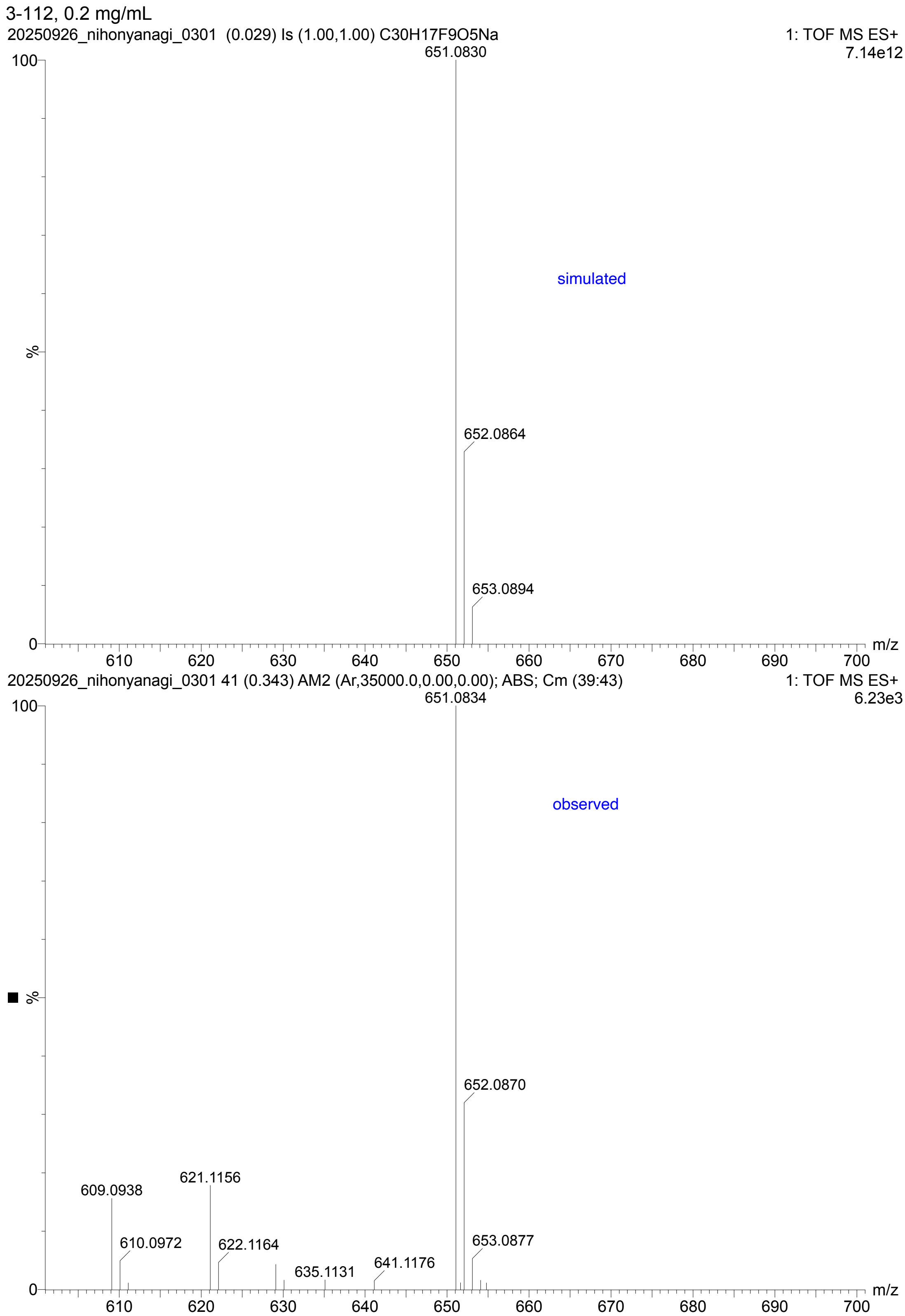

Figure S4 HRMS spectra for **Compound 1**.

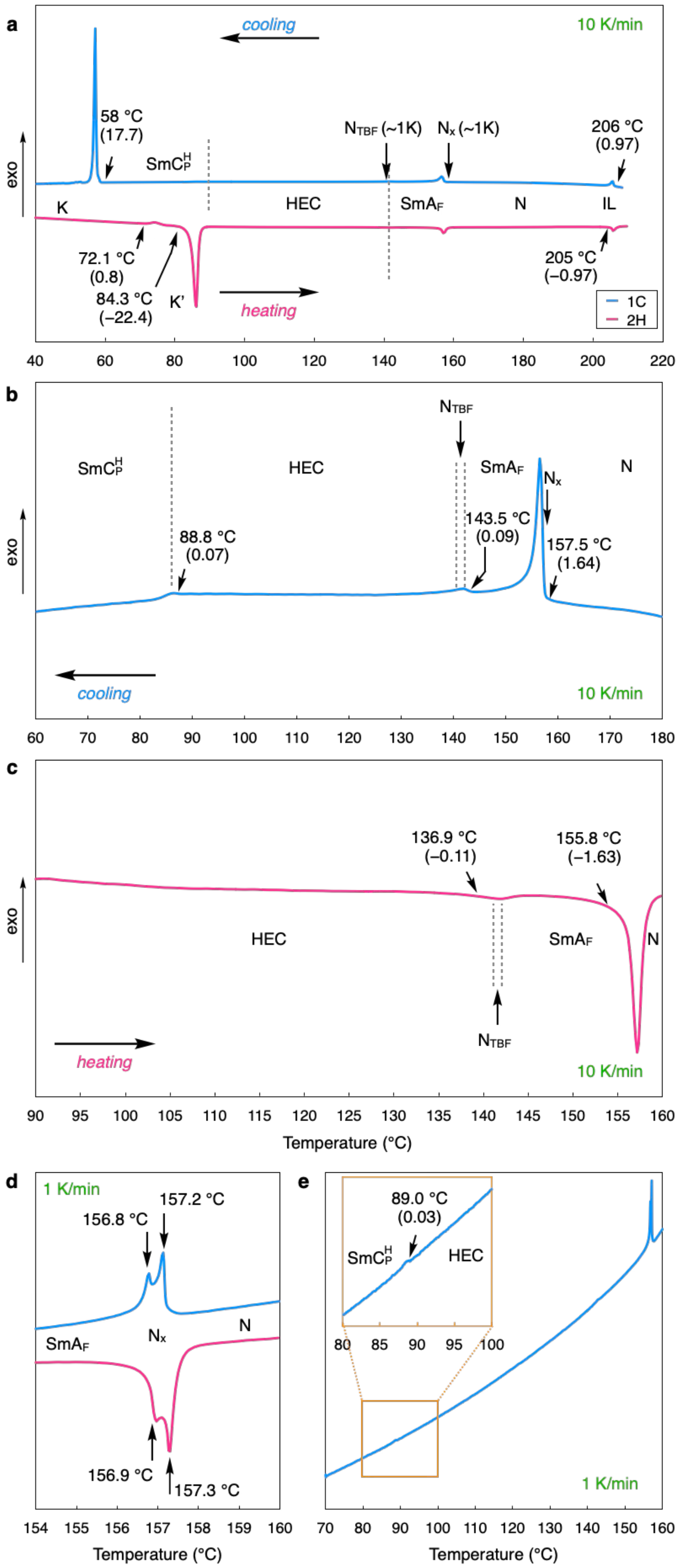

Figure S5 DSC curves for **Compound 1**. Overall curves (a) and enlarged curves during cooling (b) and heating (c). Scan rate: 10 K min^{-1}. d,e) DSC curves recorded at a scan rate of 1 K min^{-1}.

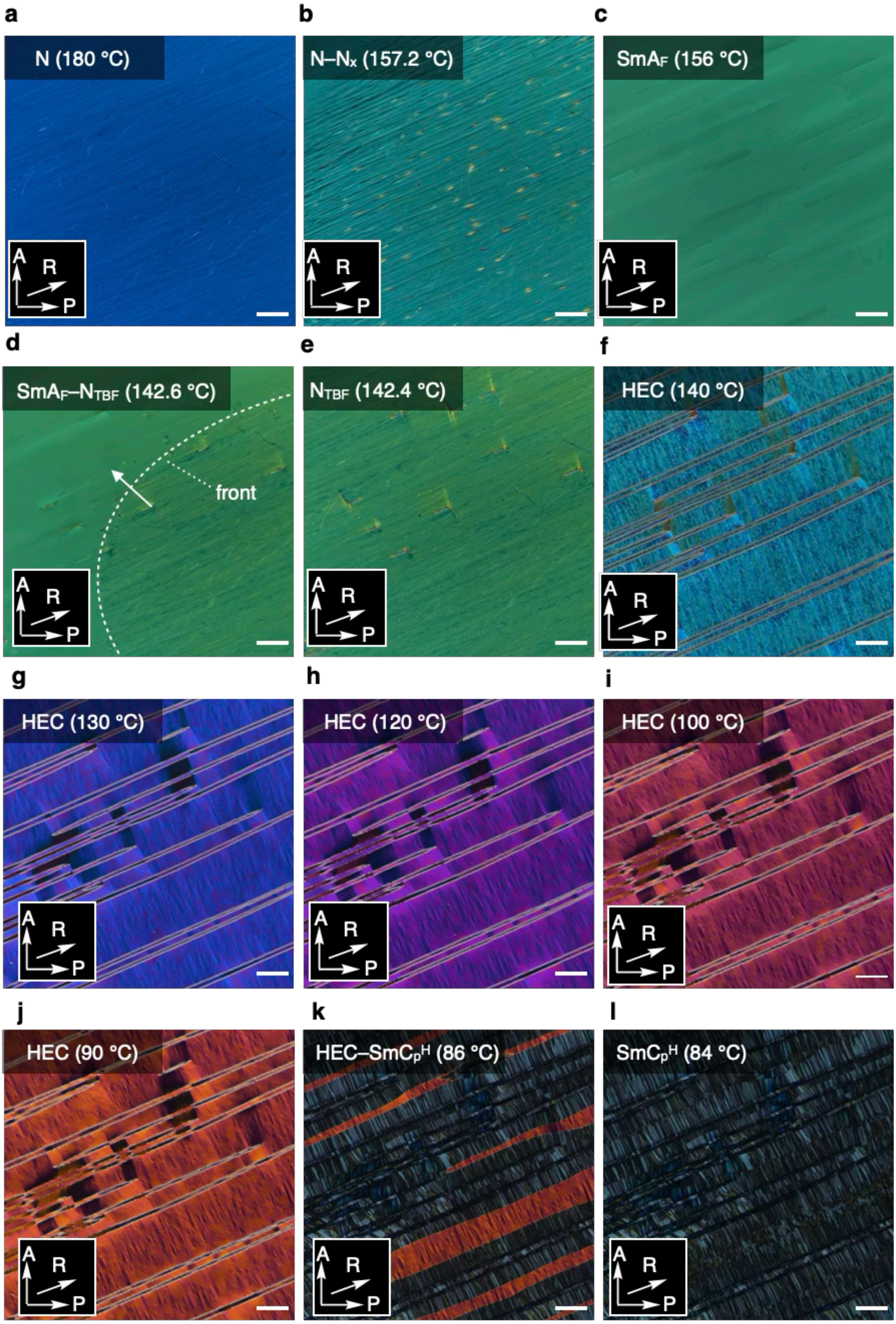

Figure S6 Complete POM images taken in various temperature in SP cell (thickness: 5 μm) for **Compound 1**. Scale bar: 100 μm.

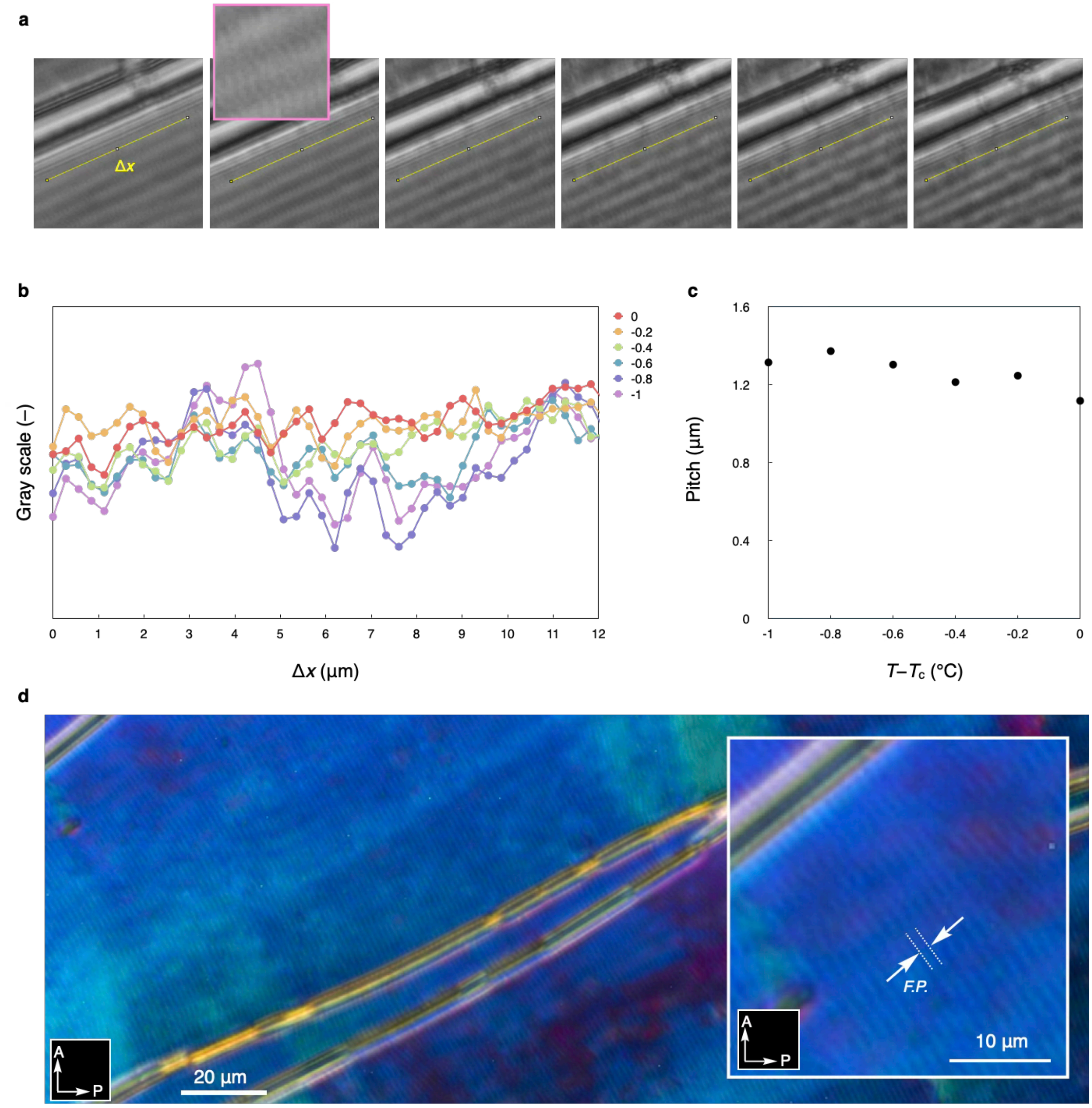

Figure S7 Image analysis to estimate helical pitch in the N_{TBF} range. a) 8-bit POM images, b) cross-sectional intensity profile perpendicular to fine stripes, c) estimated pitch vs temperature. d) POM image taken in the cover glass substrate cell without alignment layer and spacers. Similarly, the full pitch 1.3 μm was estimated.

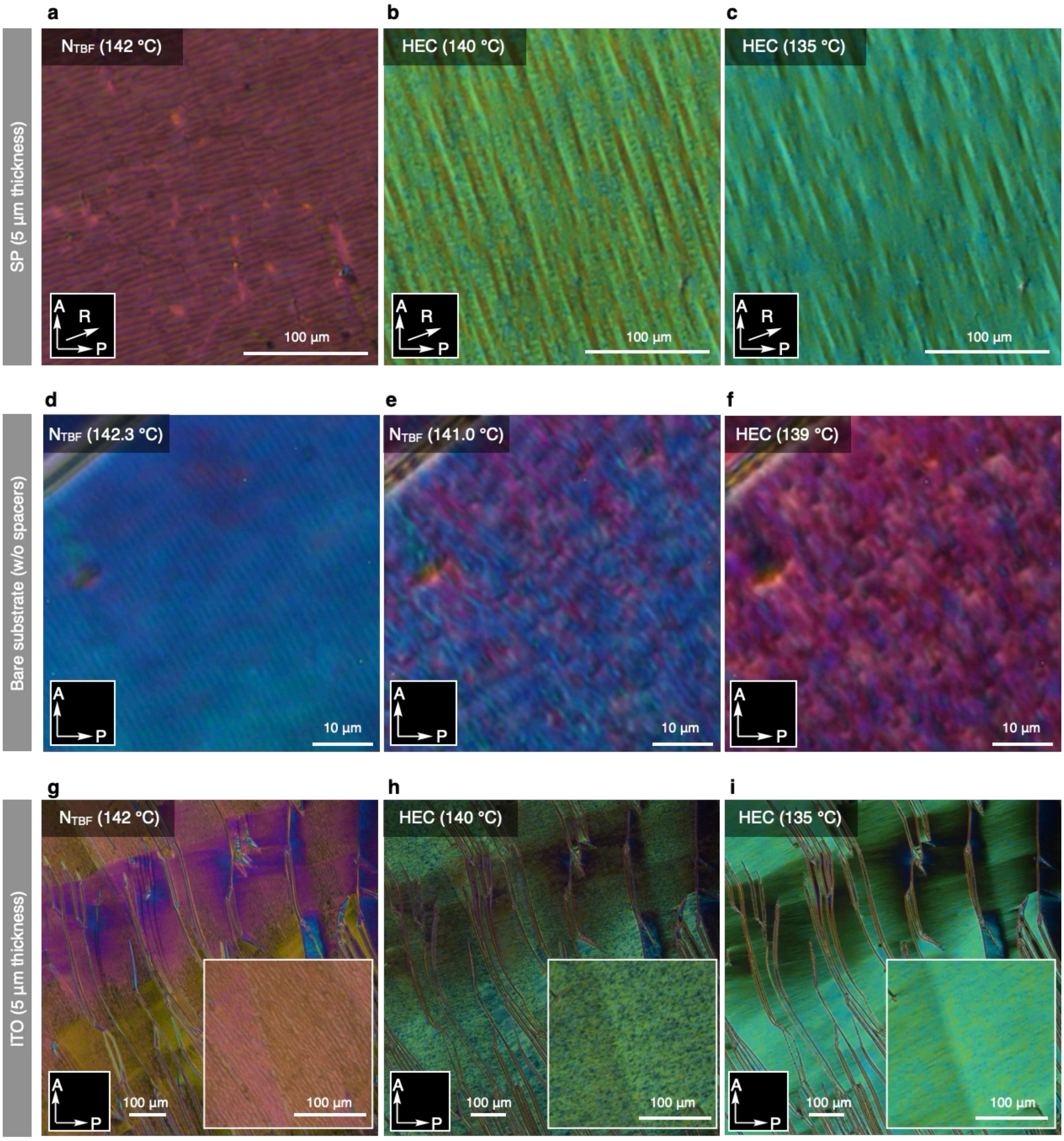

Figure S8 Changes in POM texture taken in various temperature in SP (another lot) (a–c), bare substrate without spacers (d–f) and ITO cells (g–h) for **Compound 1**. Thickness: SP cell, 5.0 µm; ITO cell, 5.0 µm.

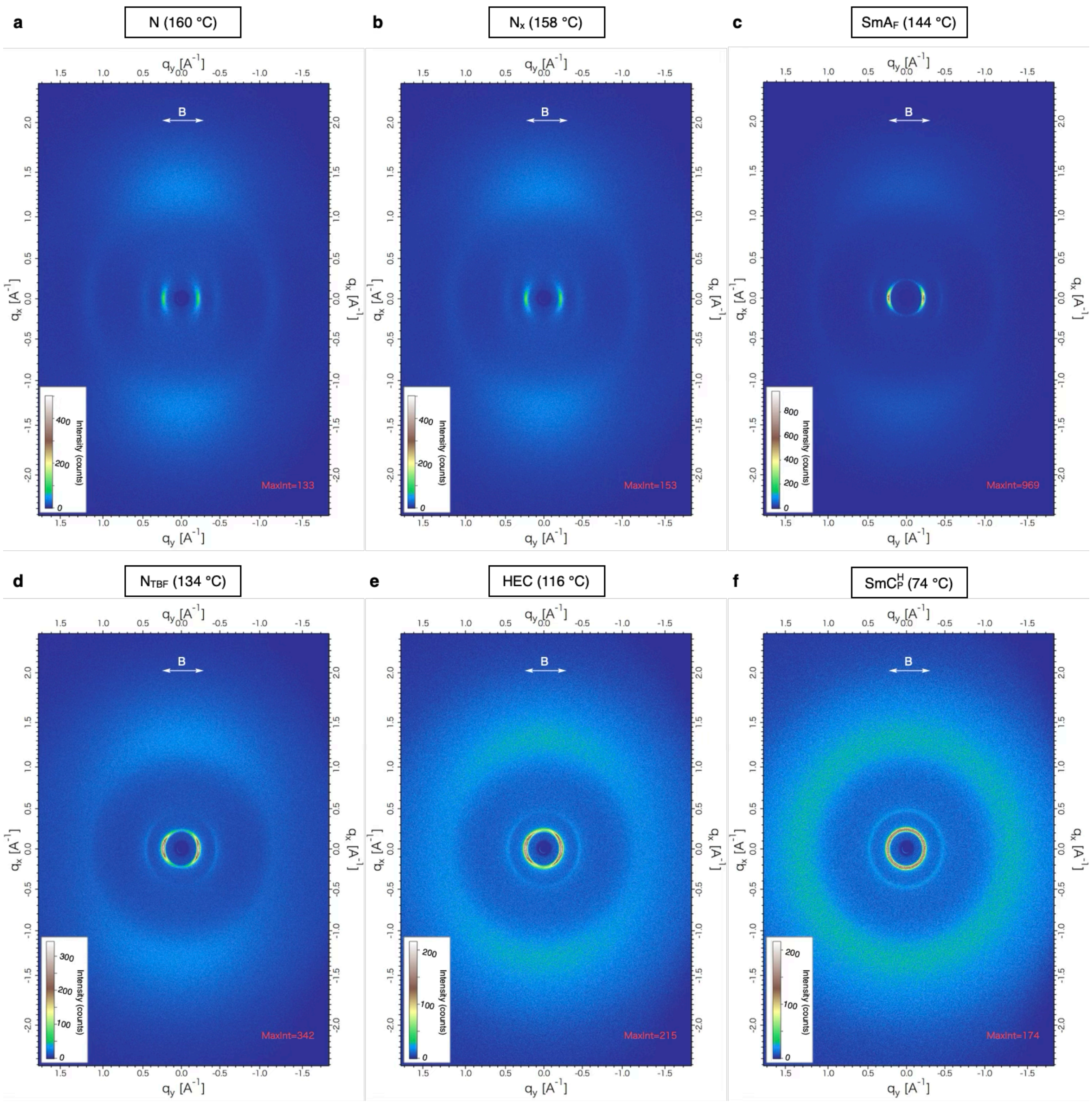

Figure S9 Full-range 2D WAXD patterns in various phases under the magnetic field (~1 T, **B** ∥ **n**) for **Compound 1**.

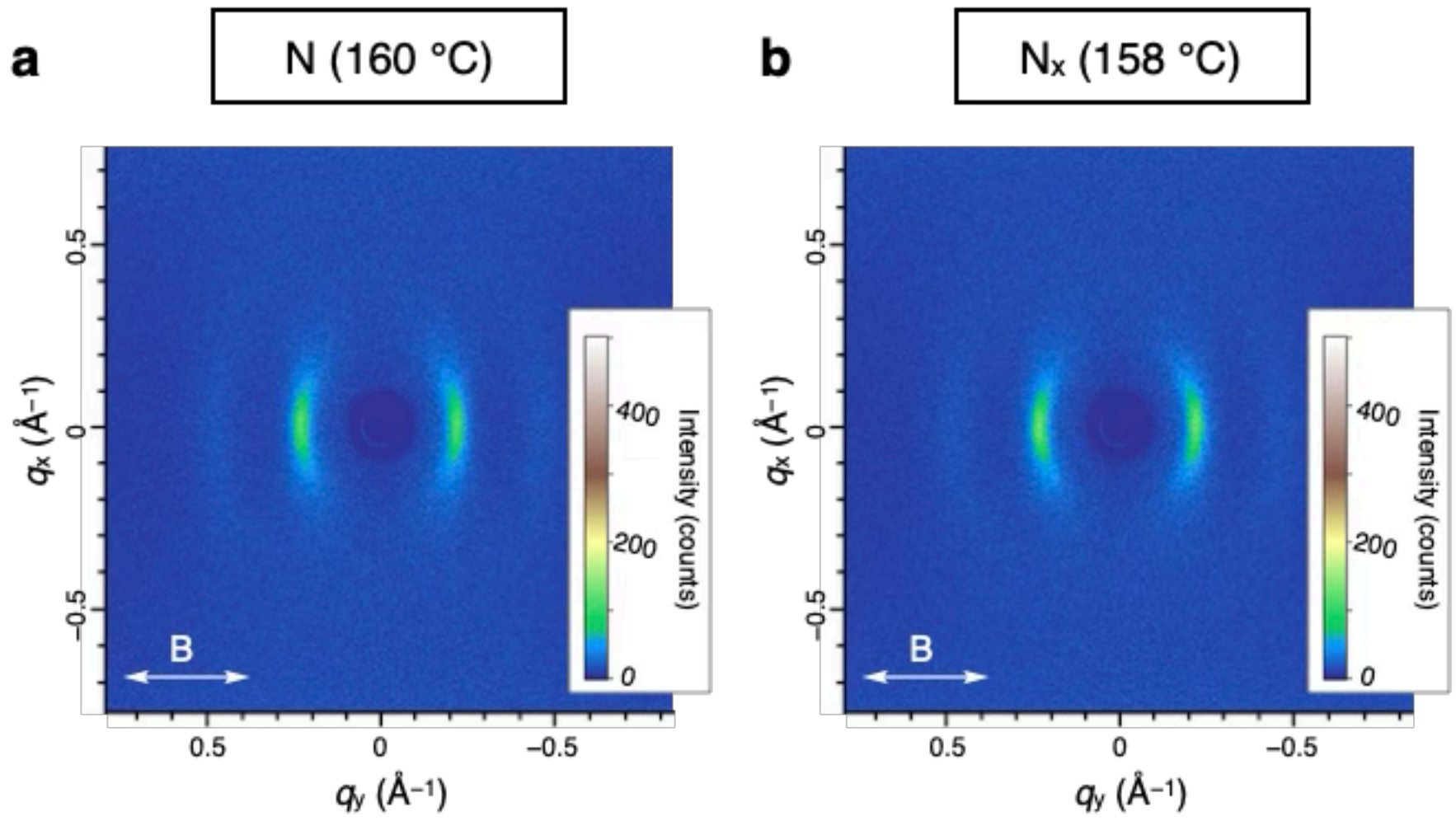

Figure S10 2D WAXD patterns in the N (a) and N_x (b) phase under the magnetic field (~1 T, **B ∥ n**) for **Compound 1**.

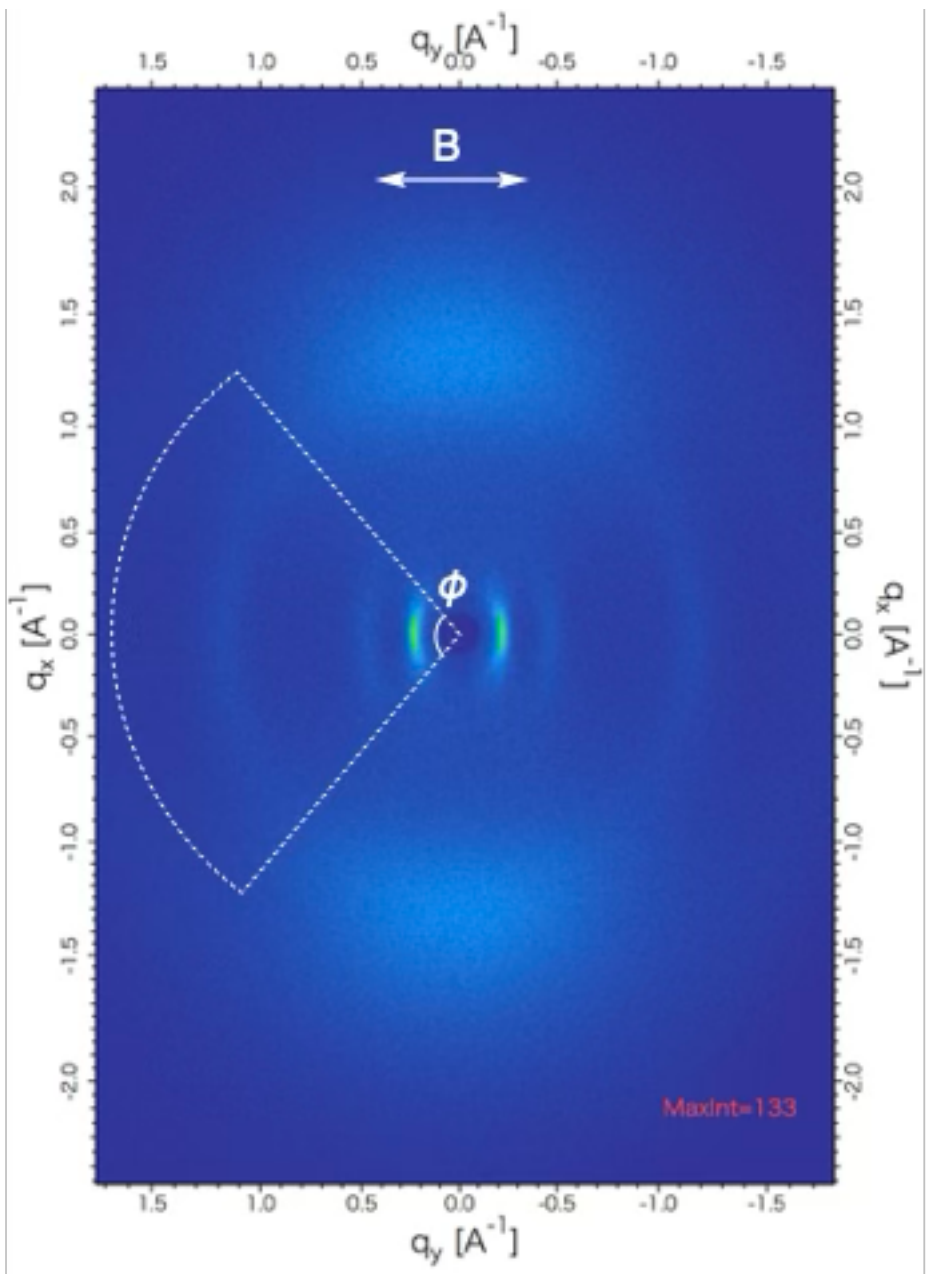

Figure S11 Box scan area (φ = 100°) on 2D X-ray pattern for generating 1D X-ray diffractogram.

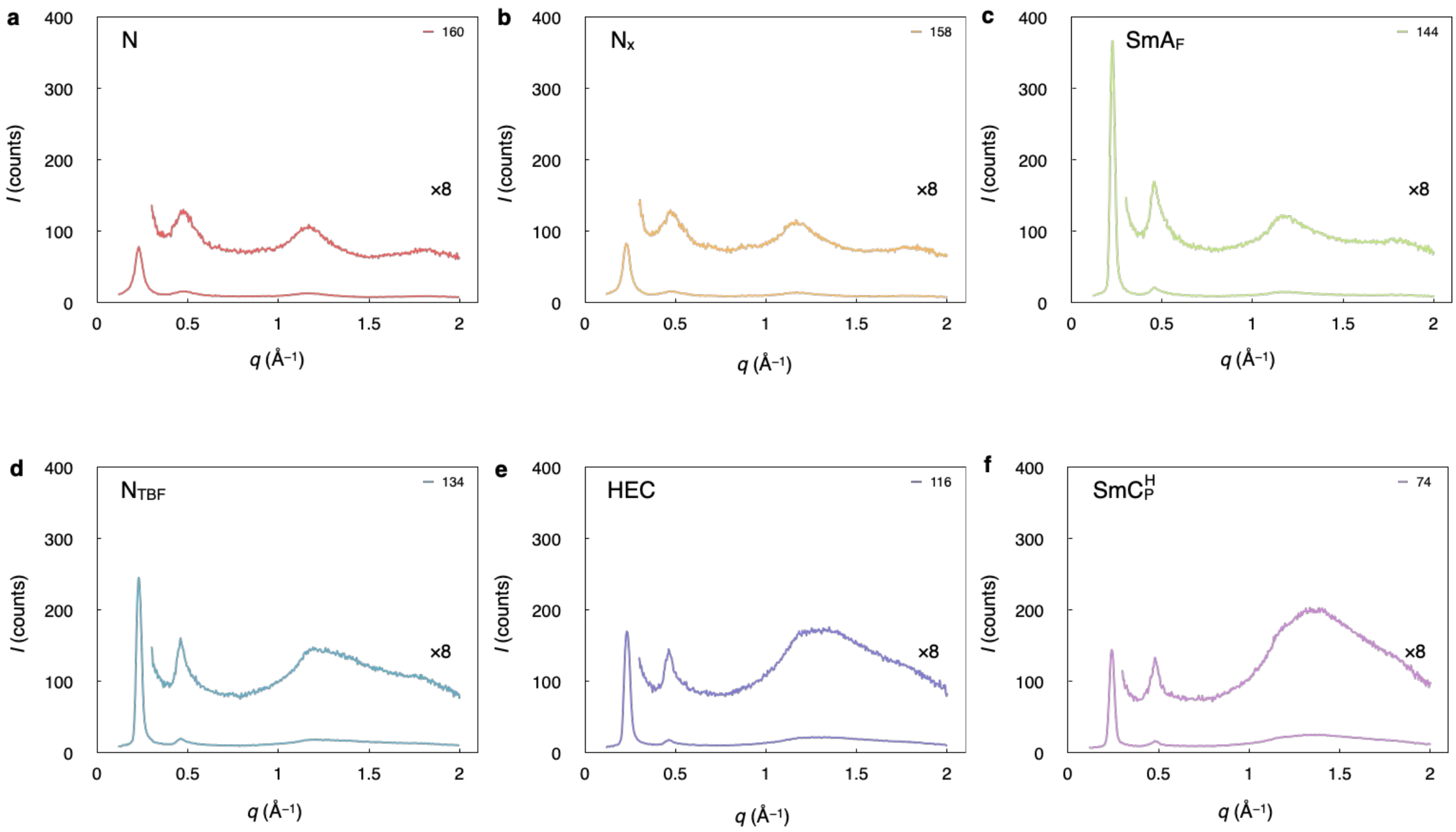

Figure S12 1D X-ray diffractogram along the equator direction (**n** ‖ **B**) from a box scan area (φ = 100°).

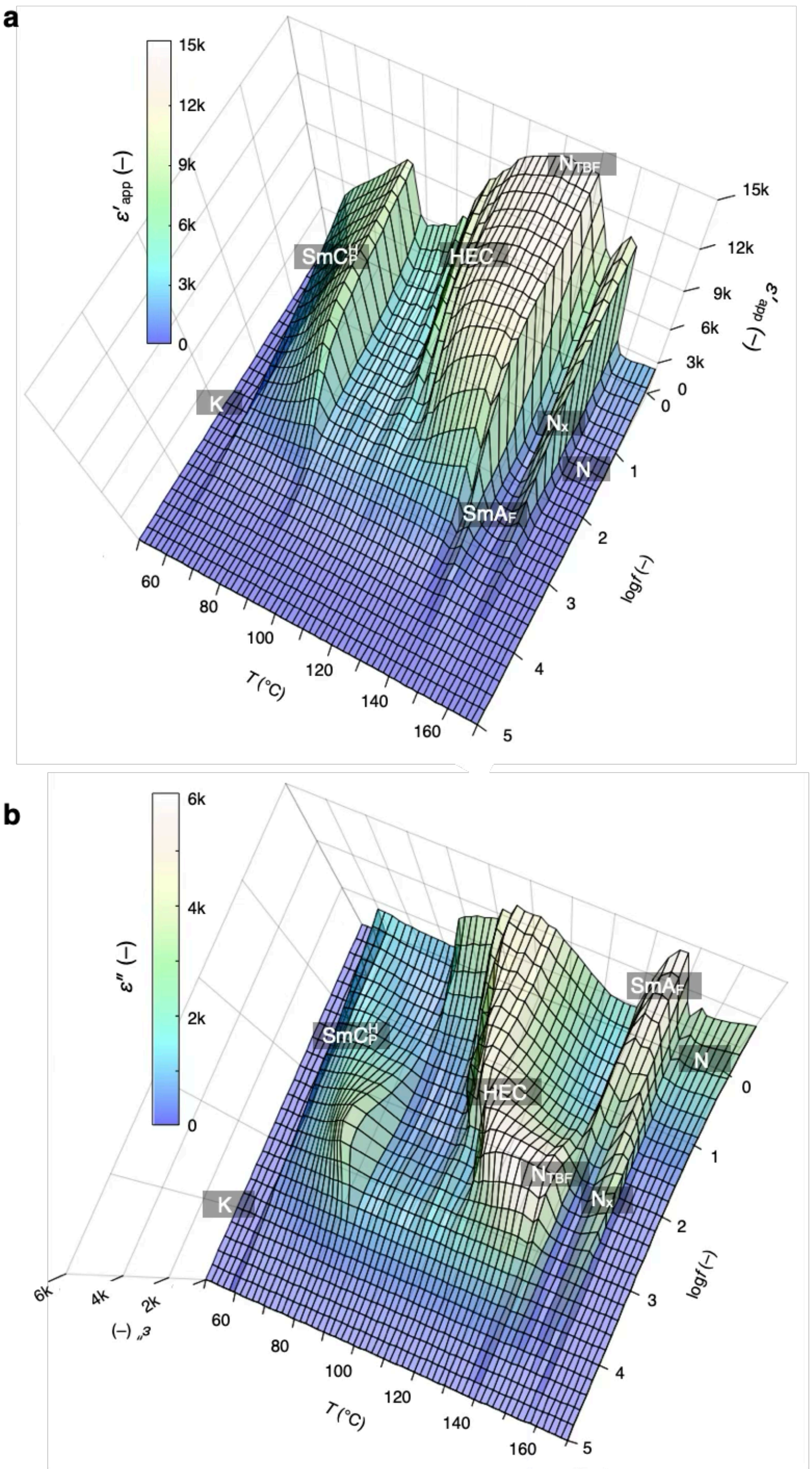

Figure S13 3D BDS data for **Compound 1** in various temperatures. (a) Apparent dielectric permittivity vs log*f*, Temperature; (b) dielectric loss vs log*f*, Temperature.

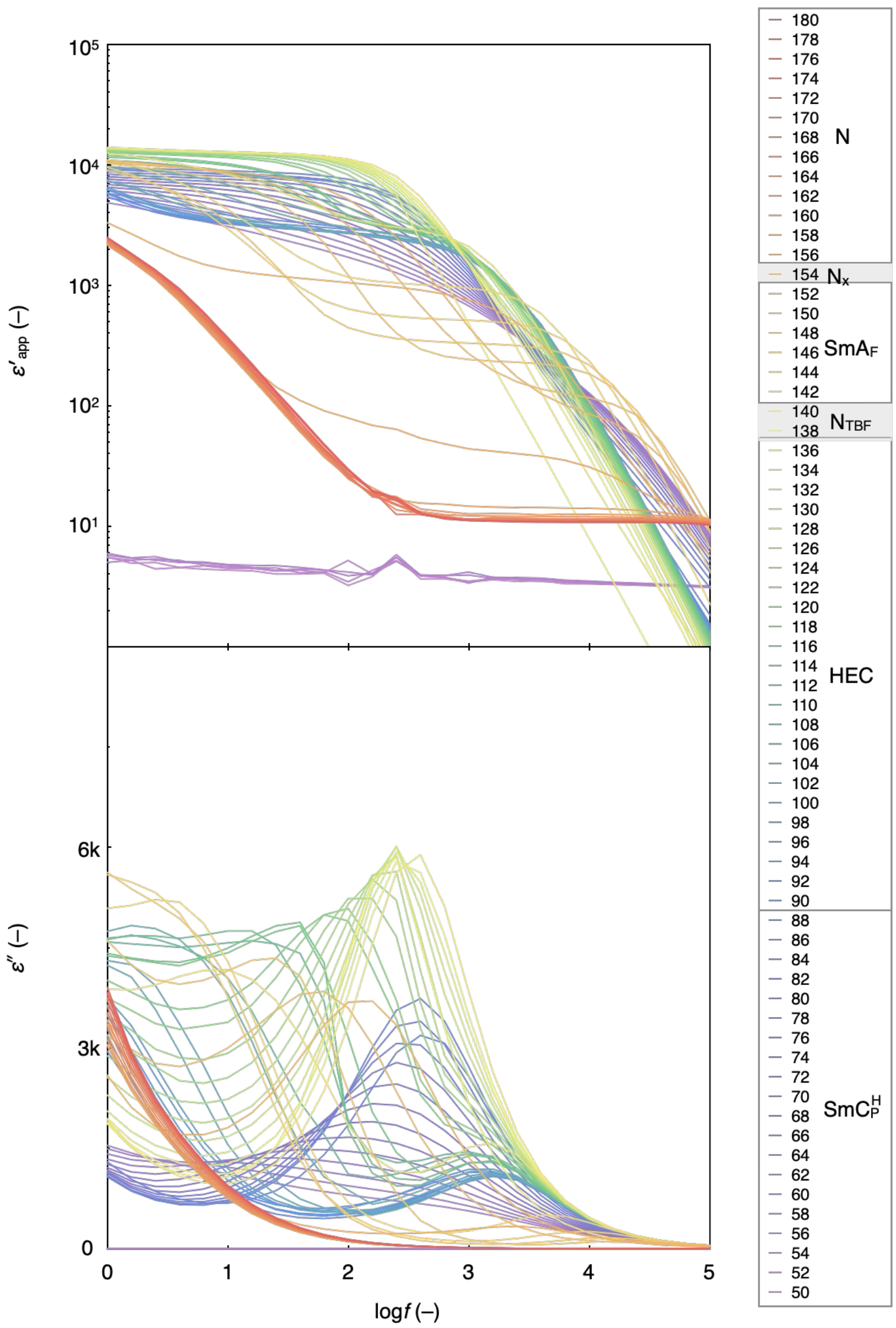

Figure S14 Complete BDS data for **Compound 1** in various temperatures.

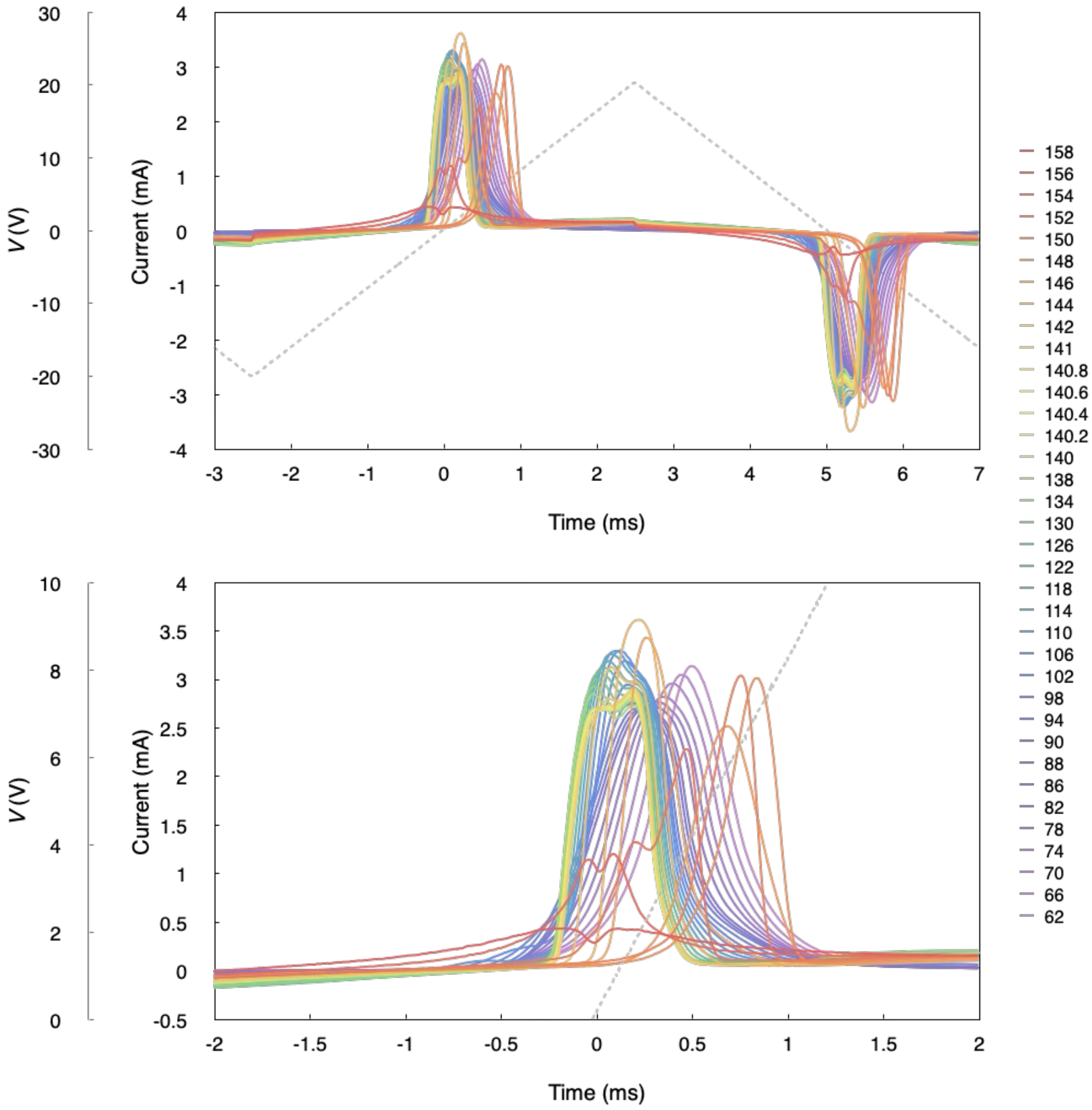

Figure S15 Complete PRC data in various temperatures for **Compound 1**. Overall profile (a) and enlarged one (b).

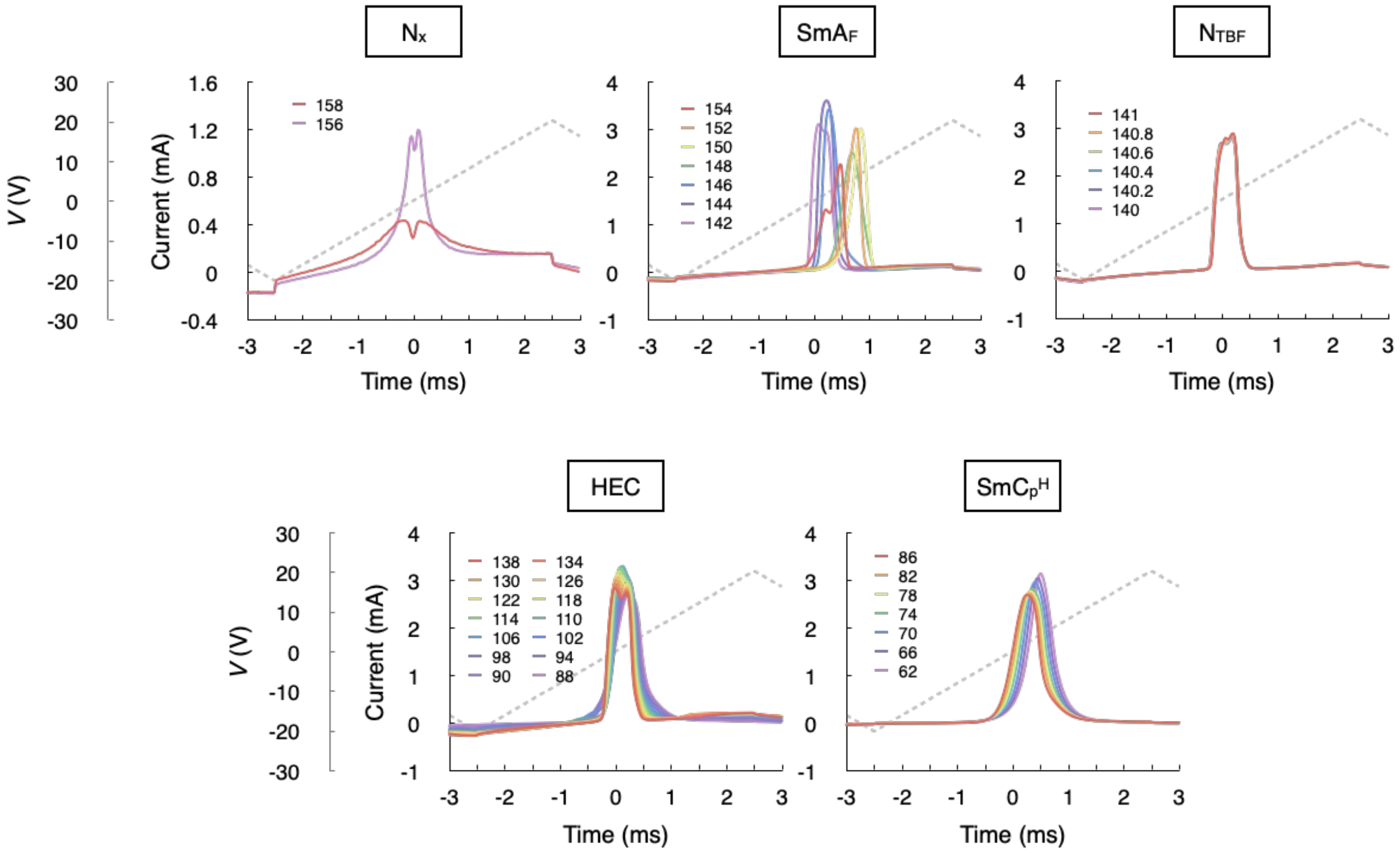

Figure S16 Complete PRC data with applied voltage profile in various LC regions for **Compound 1**.

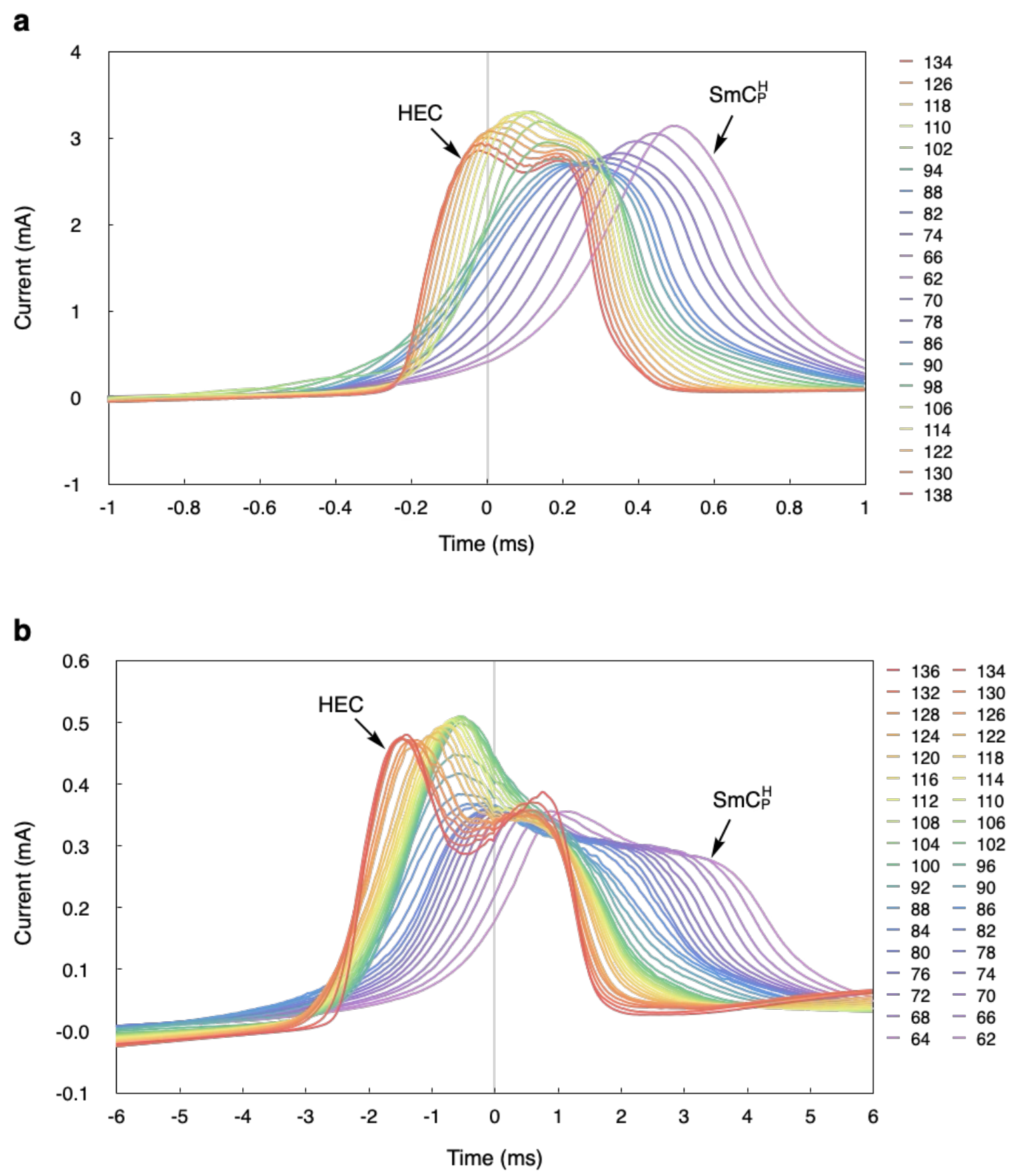

Figure S17 Comparison of PRC data within the HEC and SmC$_P^H$ ranges for **Compound 1**. PRC data obtained under conditions: (a) V_{pp} = 40 V, f = 100 Hz (b) V_{pp} = 20 V, f = 20 Hz.

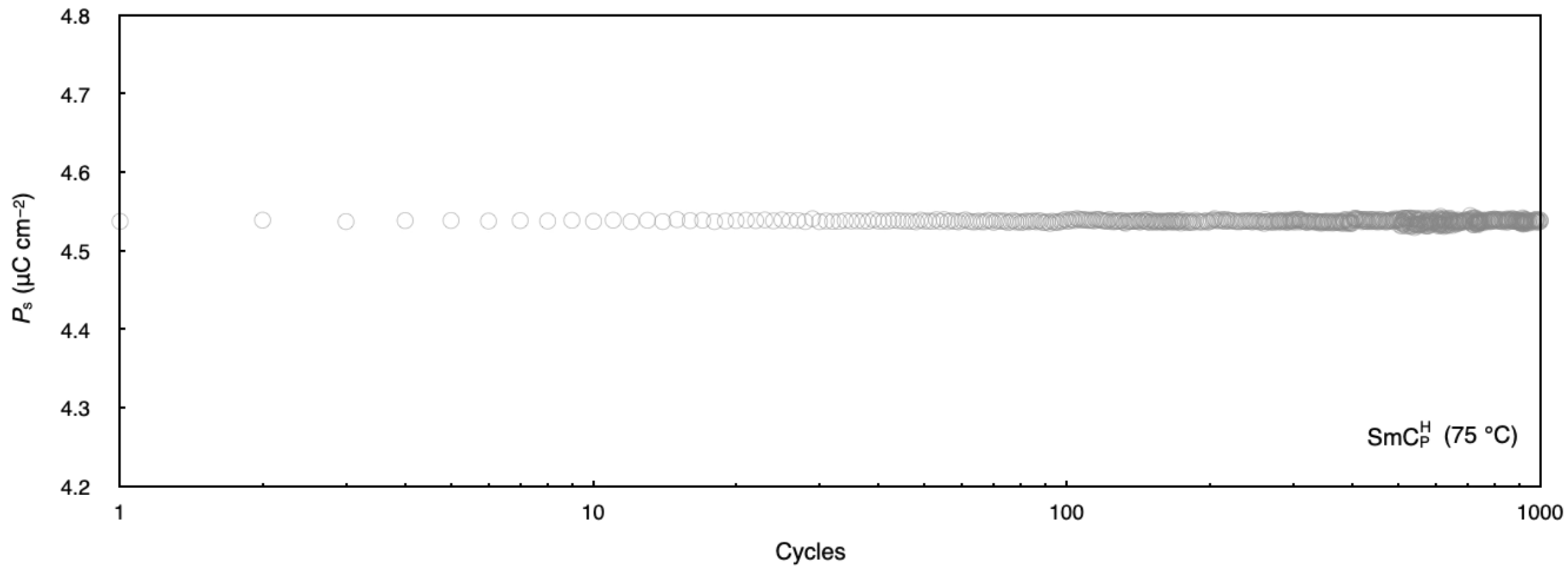

Figure S18 *E*-field fatigue test in the SmC_P^H (75 °C) for **3-212**.

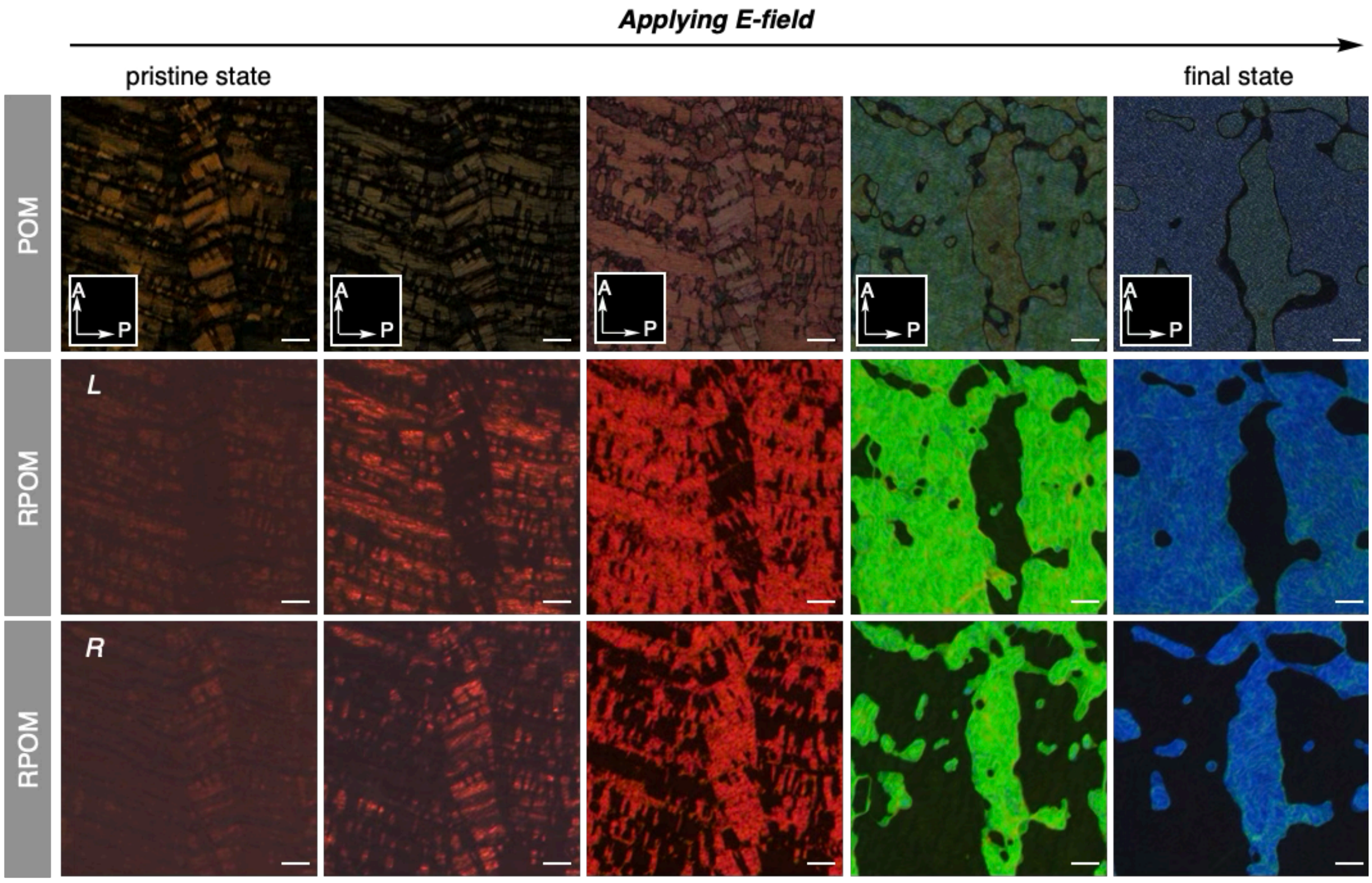

Figure S19 Changes in POM and RPOM images in the SmC_p^H phase (75 °C) under AC *E*-field.

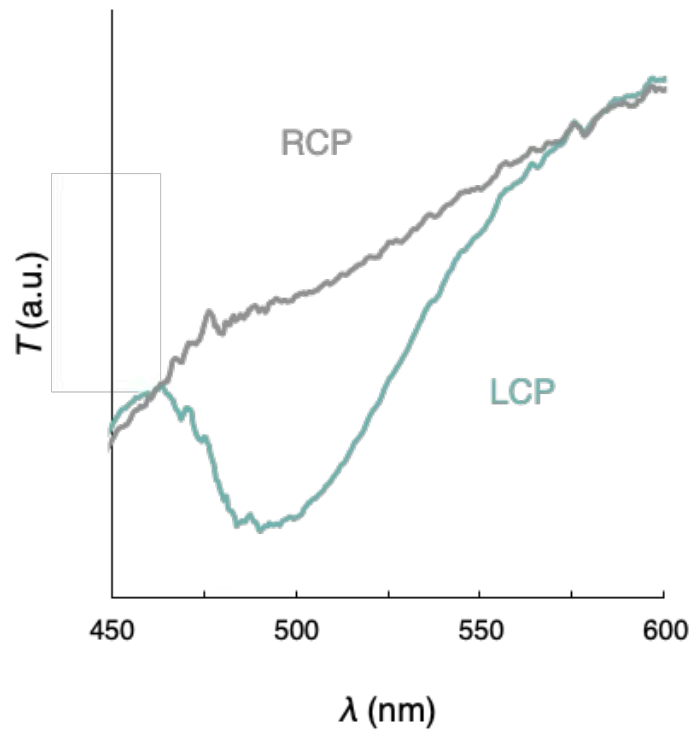

Figure S20 Transmittance spectra at the local homochiral domain through right- and left-handed circular polarizers (RCP and LCP) in the SmC_p^H phase (75 °C) under *E*-field.

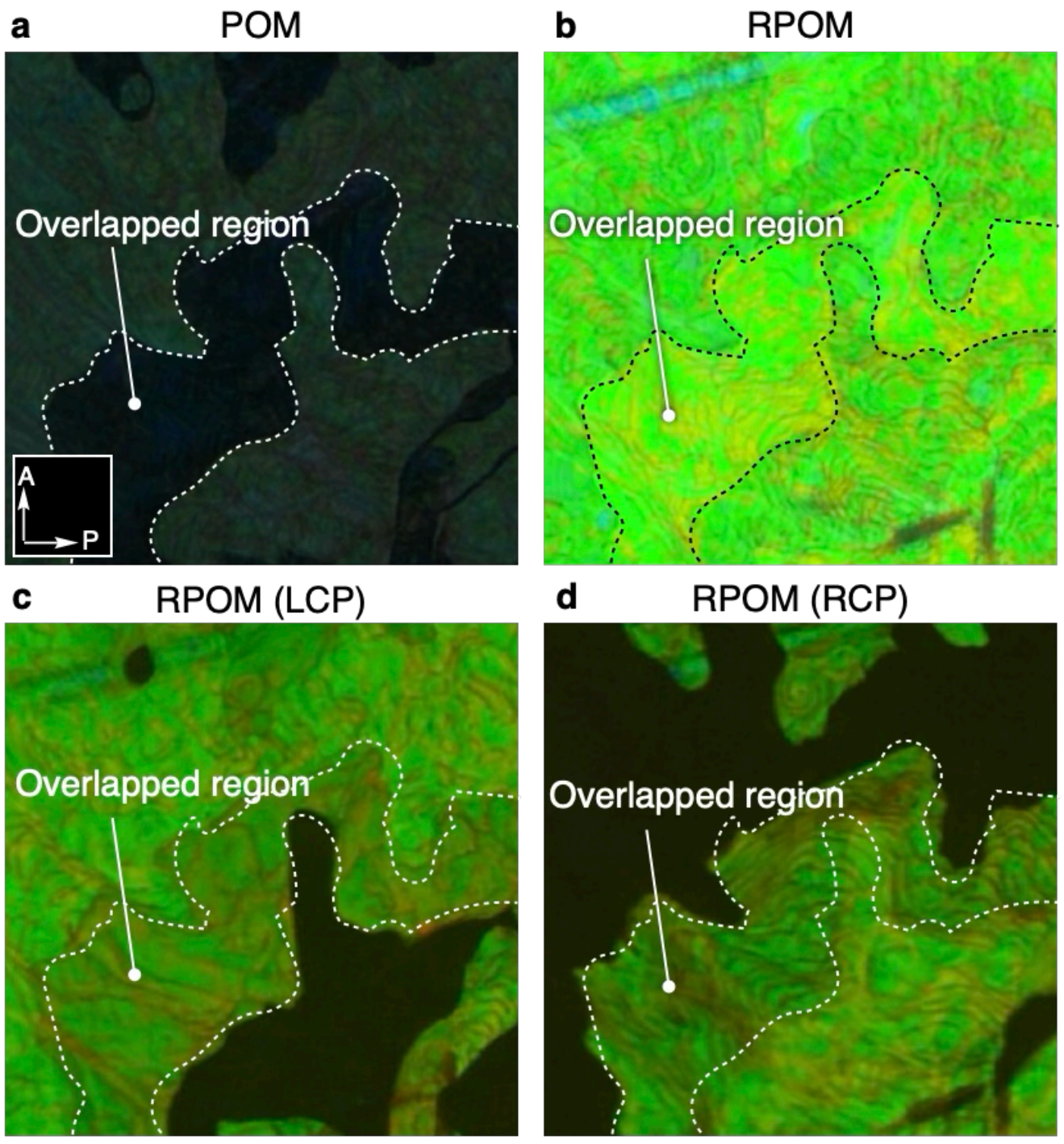

Figure S21 Extend POM images in the SmC_P^H phase (75 °C) under *E*-field. a) POM, b) reflective mode POM (RPOM) and RPOM images through left- (c) and right- (d) handed circular polarizers.

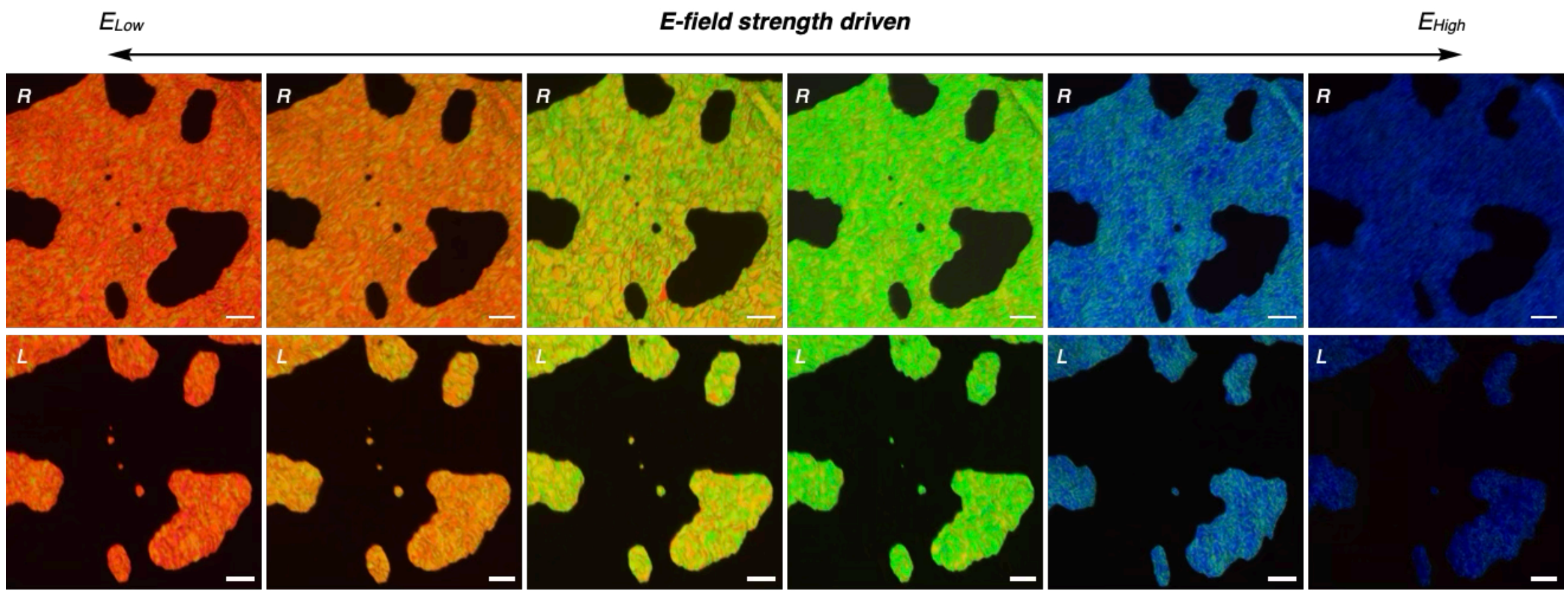

Figure S22 Extend RPOM images of the SmC_P^H phase (75 °C) under *E*-field. These images were taken in thin LC cell (4.5 μm) through RCP and LCP.

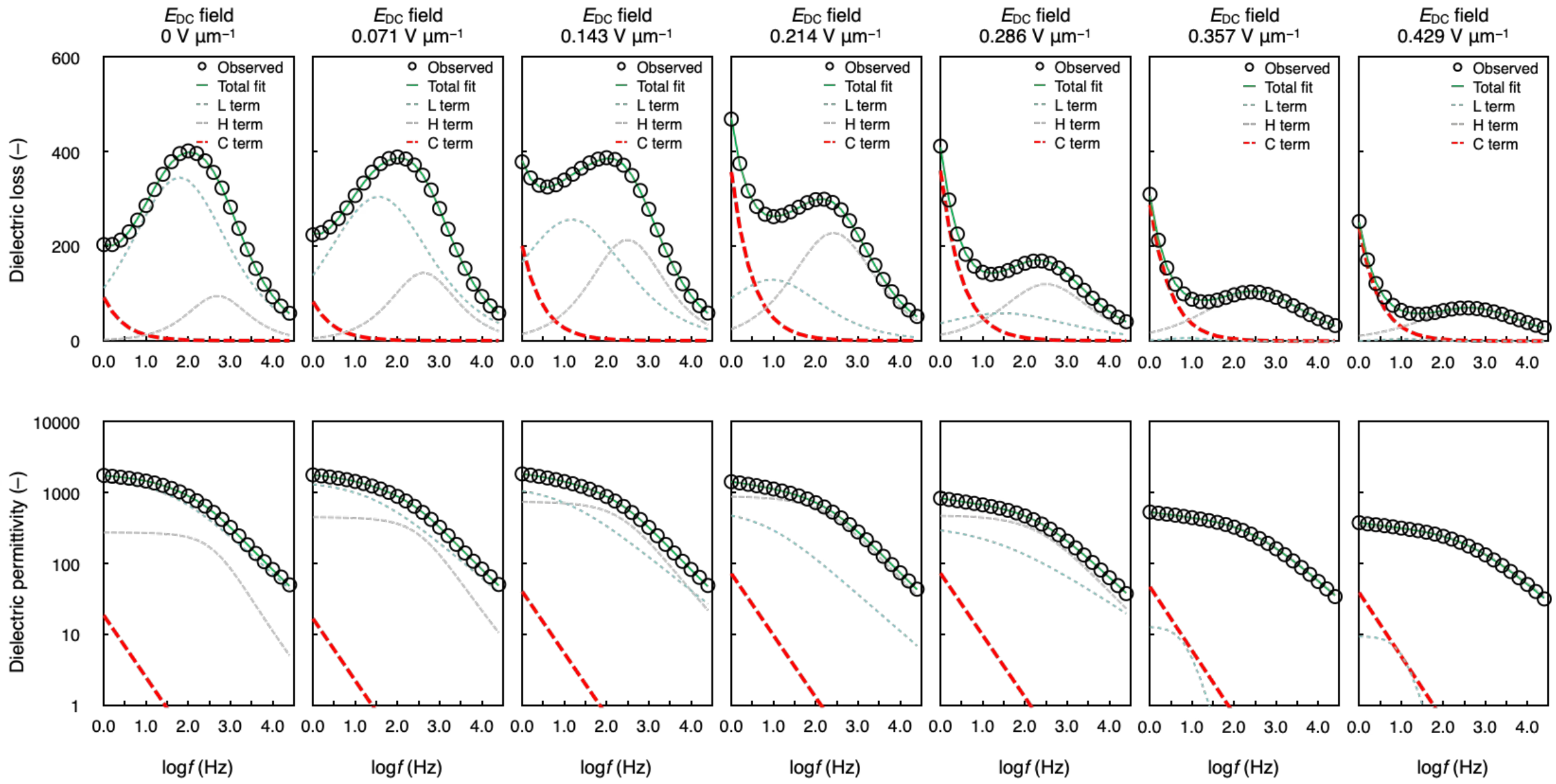

Figure S23 DC-bias-dependent BDS analysis in the SmC_P^H phase (75 °C) for **Compound 1**. Frequency dependence of the dielectric loss (ε'') (upper panels), and dielectric permittivity (ε') (lower panels), measured using a small AC probing voltage (100 mV) superimposed on the indicated E_{DC}- fields. Open circles represent the experimental data, and solid lines represent the total fitted spectra obtained using two Havriliak–Negami relaxation contributions together with a low-frequency conductivity-related term. The dashed lines show the individual contributions of the low-frequency relaxation mode (L term), high-frequency relaxation mode (H term), and conductivity term (C term).

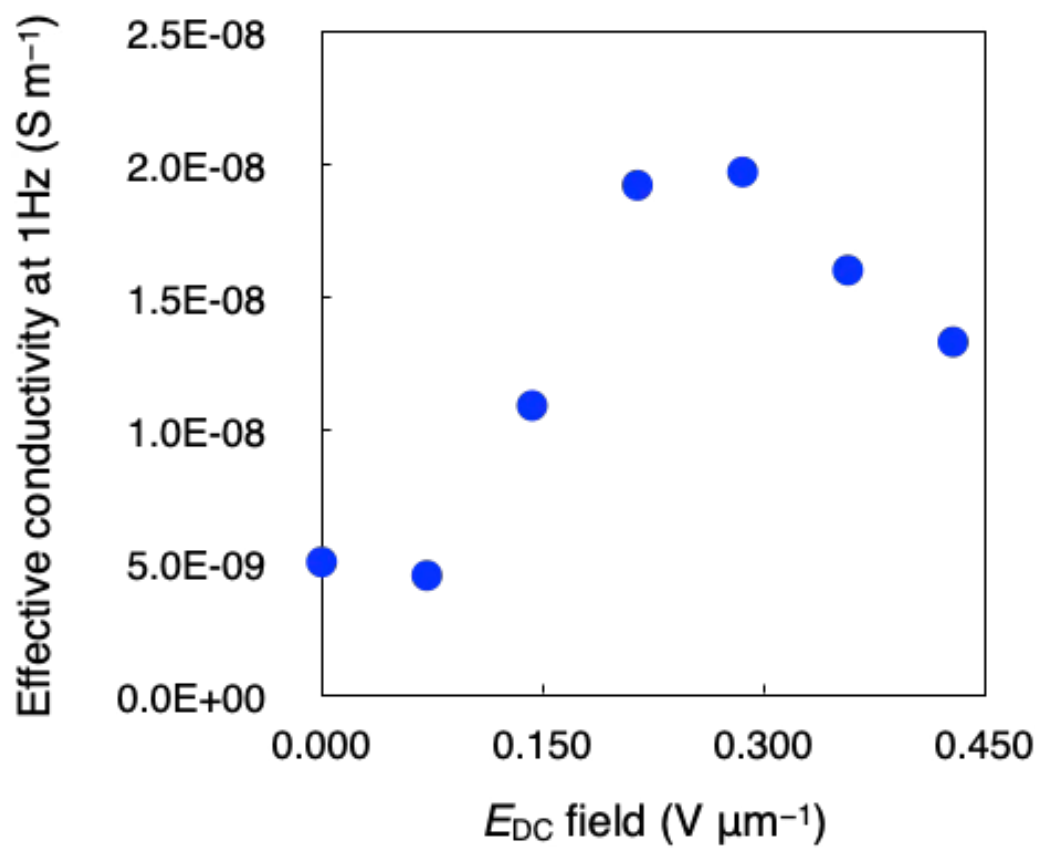

Figure S24 DC-field dependence of the effective conductivity at 1 Hz, evaluated from the dielectric-loss contribution of the fitted conductivity term (C term).

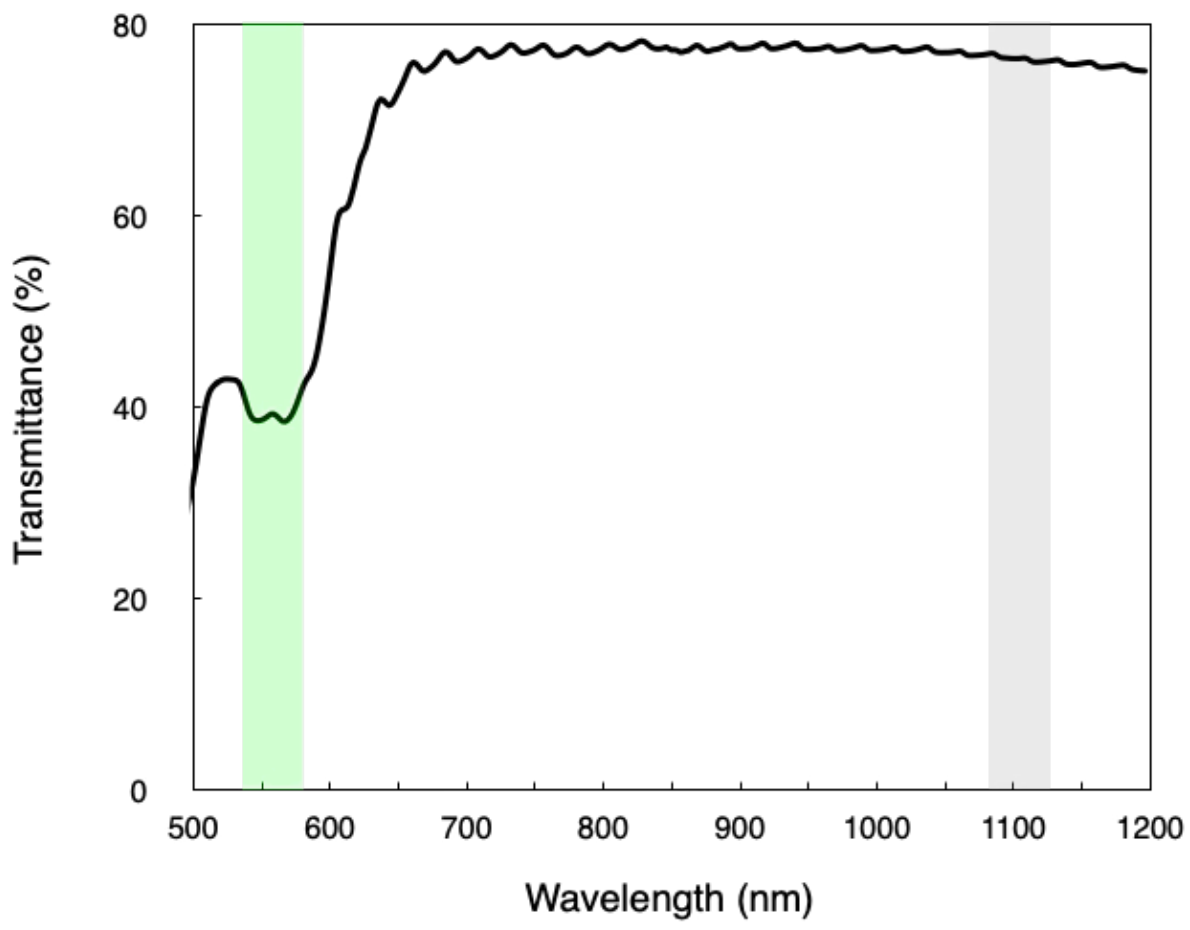

Figure S25 UV-Vis-NIR spectra recorded in the SmC_P^H phase (75 °C) for **Compound 1** under *E*-field of 0.75 V_{pp} μm^{-1}.

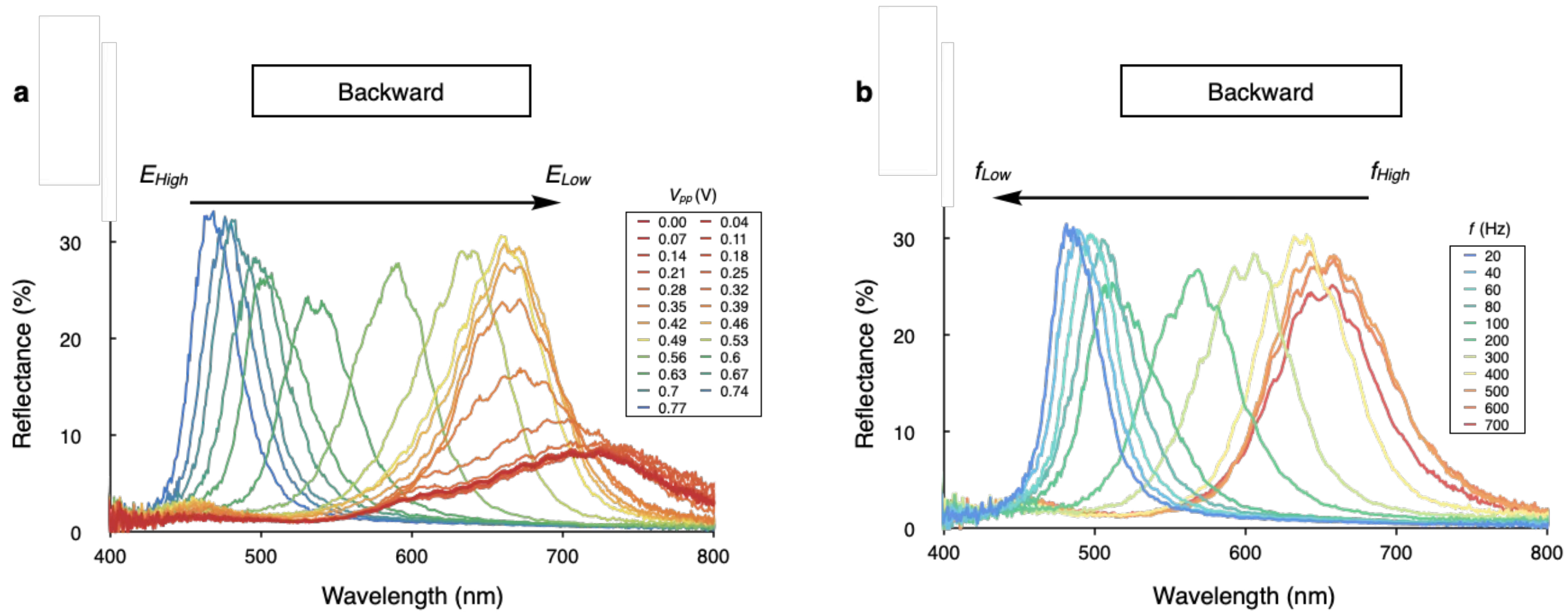

Figure S26 Backward process of *E*-field- (a) and Frequency- (b) driven modulation for reflection band in the SmC_P^H phase (75 °C): (a) E_{High} to E_{low}, (b) f_{High} to f_{Low}.

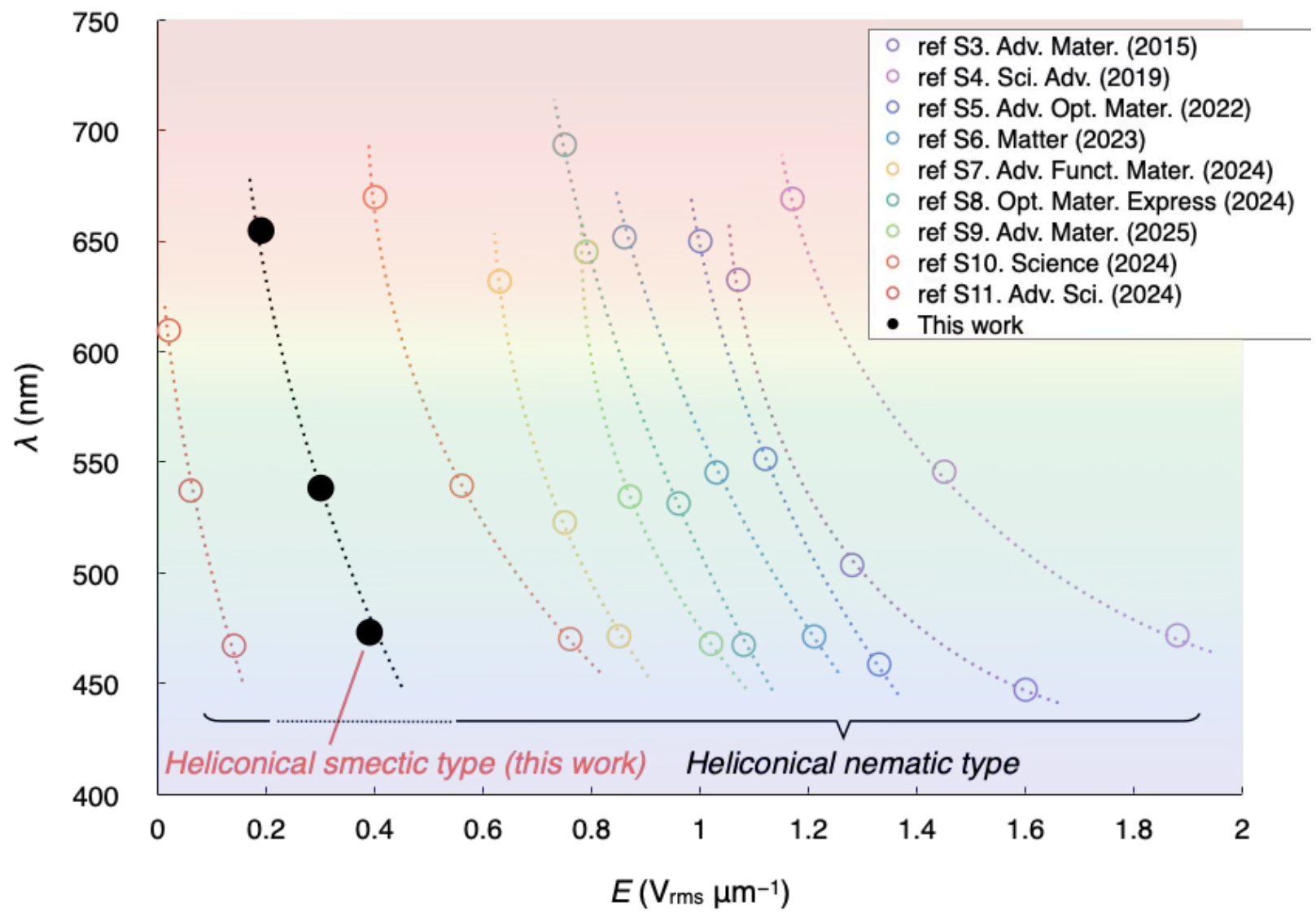

Figure S27. *E*-field-dependent peak reflection wavelength (λ) of representative heliconical nematic-type systems reported in the literature [S3–S11] and the present heliconical smectic-type system. Data were extracted from the corresponding publications. Dotted curves are guides to the eye. In the heliconical smectic system, the plots are not smoothly connected because of the distinct scaling behavior in different regimes.

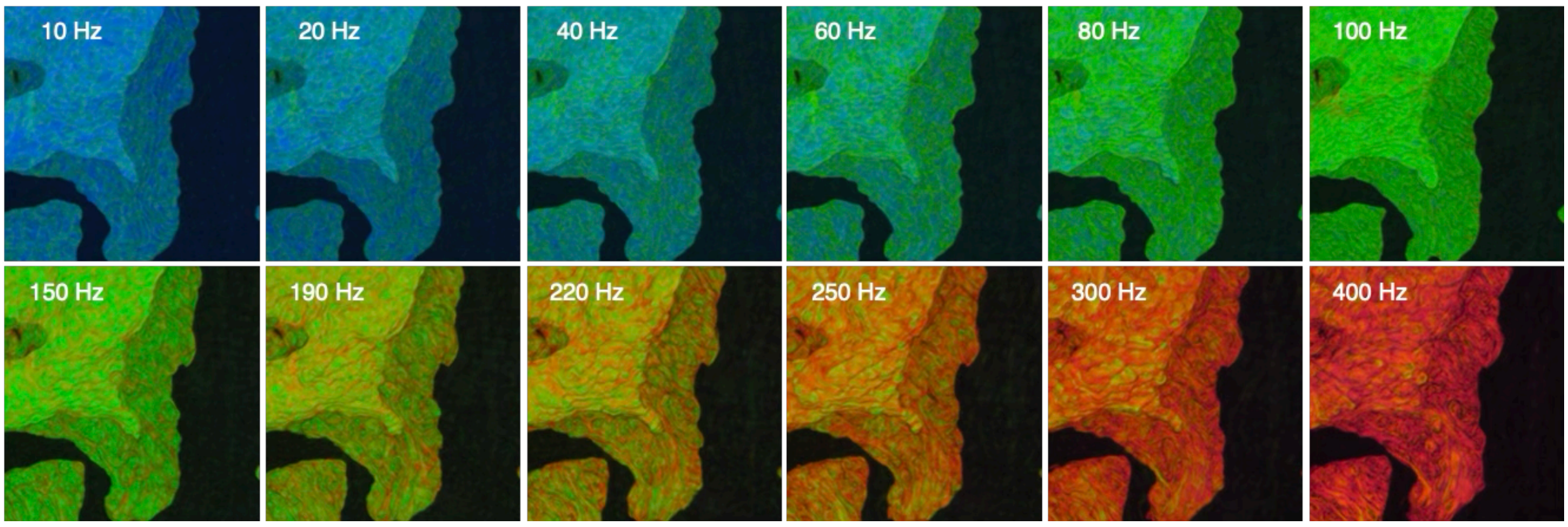

Figure S28 Frequency-dependent RPOM images (f = 10–400 Hz) taken through the RCP in the SmC_P^H phase (75 °C) under fixed E-field (square waveform, 9.0 V_{pp}).

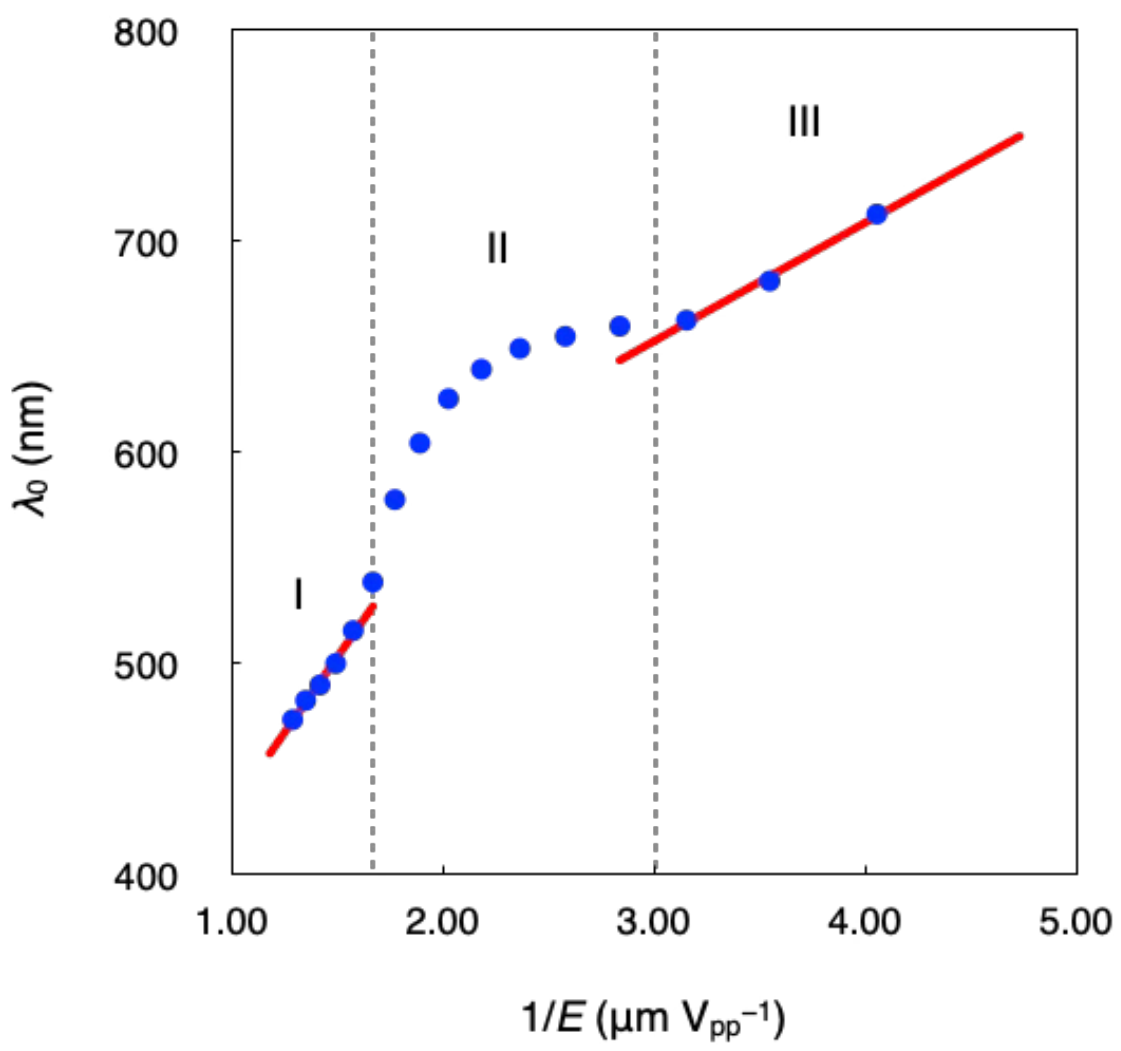

Figure S29 λ_0 vs $1/E$ in the $\mathrm{SmC_P^H}$ phase (75 °C). The helical pitch exhibits distinct E-field-dependent modulations across three regimes (I, II, and III). In regions I and III, λ_0 exhibits inverse-field scaling ($\lambda_0 \propto 1/E$) and is described by $\lambda_0 = 143/E + 288$ and $\lambda_0 = 56.1/E + 484$, respectively.

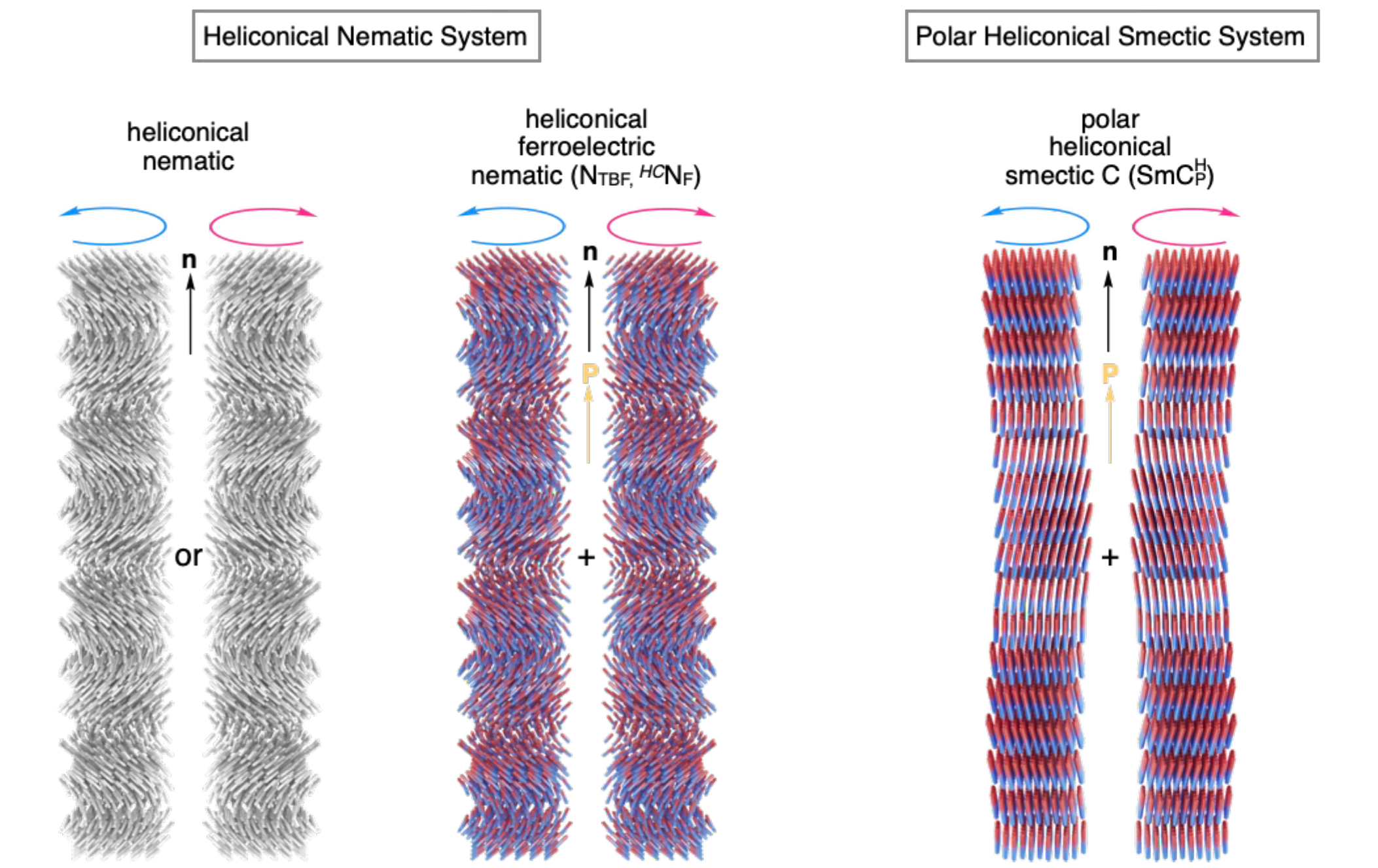

	Heliconical nematic	Heliconical ferroelectric nematic (N_{TBF}, $^{HC}N_F$)	Polar heliconical smectic C: *Previous work (2025)*	Polar heliconical smectic C: *This work (2026)*
**Components**	Complex (rod-like molecule + dimeric molecule + chiral dopant)	**Simple** (**single** polar rod-like molecule)	**Simple** (**single** polar rod-like molecule)	**Simple** (**single** polar rod-like molecule)
**Properties**				
Alignment layer	Need	Need	**Needless**	**Needless**
E_{AC} (red ⇄ blue)	0.79–1.02 (V μm^{-1})	Not reported	Not achieved	**0.23–0.39 (V μm^{-1})**
E_{DC} (red ⇄ blue)	Not reported	0.35–0.75(V μm^{-1})	Not achieved	Not achieved
f (red ⇄ blue)	73.5–1(kHz)	Not reported	Not achieved	**0.4–0.01 (kHz)**
**Functionalities**				
Multicolor modulation by |E| and f	✓	Not reported	Not achieved	**✓ (This work)**
Tunable SHG	Complicated	Not reported	Not achieved	**✓ (This work)**

Figure S30 Comparison of key material characteristics between heliconical nematic and polar heliconical smectic systems.

Supporting Tables (Table S1, S2)

Table S1 Phase transition temperature (°C) and enthalpy changes (kJ mol^{-1}, in parentheses) for **Compound 1**. Rate: 10 K min^{-1} and 1 K min^{-1}.

Process	K		SmC_P^H		HEC		N_{TBF}		SmA_F		N_x		N		IL
1C	•	58.0<br>(17.7)	•	88.8<br>(0.07)<br>(0.03)[b]	•	140[a]<br>(i)	•	143.0[a]<br>(ii)	•	156.8[a,b,c]<br>(iii)	•	157.2[a,b,c]<br>(iv)	•	206<br>(0.97)	•
2H	•	84.3<br>(–22.4)	–		•	136.9[a]<br>(v)	•	–[d]<br>(vi)	•	156.9[a,b,c]<br>(vii)	•	157.3[a,b,c]<br>(viii)	•	205<br>(–0.97)	•

[a] Overlapping peaks on DSC trace, temperature taken from POM and/or DSC. i + ii = 0.05 kJ mol^{-1}, iii + iv = 1.17 kJ mol^{-1}, v + vi = –0.05 kJ mol^{-1}, vii + viii = –0.145 kJ mol^{-1}. [b] Rate: 1 K min^{-1}. [c] $T_{peak\text{-}top}$. [d] Undeterminable.

Table S2 The molecular parameters of energy-minimized conformations calculated by DFT for **Compound 1** and **Model I**.

Entry	Vector X	Vector Y	Vector Z	μ (D)	β (deg)[a]
**Compound 1**	10.0869	−4.1888	−1.8005	11.07	24.32
**Model I** [b]	9.6458	−2.8264	−1.3728	10.24	18.04

[a] An angle between the permanent dipole moment (μ) and long molecular axis; [b] [S12]

Supporting Videos (Video S1)

Video S1 Multicolor modulation in the SmC_P^H phase of **Compound 1** (75 °C). Conditions: ITO glass cell (gap: 14.1 μm^{-1}), frequency: 100 Hz, *E*-field (square waveform): 0 to 11 V_{pp}.

Video S2 Multicolor modulation in the SmC_P^H phase of **Compound 1** (75 °C). Conditions: ITO glass cell (gap: 14.1 μm^{-1}), frequency: 100 Hz, *E*-field (square waveform): 11 to 0 V_{pp}.

Video S3 Multicolor modulation in the SmC_P^H phase of **Compound 1** (75 °C). Conditions: ITO glass cell (gap: 14.1 μm^{-1}), *E*-field (square waveform): 9 V_{pp}, frequency: 10 to 400 Hz.

Video S3 Multicolor modulation in the SmC_P^H phase of **Compound 1** (75 °C). Conditions: ITO glass cell (gap: 14.1 μm^{-1}), *E*-field (square waveform): 9 V_{pp}, frequency: 400 to 10 Hz.

Video S4 POM texture (reflective mode) recorded thorough a left-handed circular polarizer in the SmC_P^H phase of **Compound 1** (75 °C). Conditions: ITO glass cell (gap: 14.1 μm^{-1}), *E*-field (square waveform): 11 V_{pp}, frequency: 100 Hz.

Video S5 POM texture (reflective mode) recorded thorough a right-handed circular polarizer in the SmC_P^H phase of **Compound 1** (75 °C). Conditions: ITO glass cell (gap: 14.1 μm^{-1}), *E*-field (square waveform): 11 V_{pp}, frequency: 100 Hz.

Supporting References

[S1] M. J. Frisch, G. W. Trucks, H. B. Schlegel, G. E. Scuseria, M. A. Robb, J. R. Cheeseman, G. Scalmani, V. Barone, B. Mennucci, G. A. Petersson, H. Nakatsuji, M. Caricato, X. Li, H. P. Hratchian, A. F. Izmaylov, J. Bloino, G. Zheng, J. L. Sonnenberg, M. Hada et al. Gaussian 09, revision E.01; Gaussian, Inc.: Wallingford, CT, (2009).

[S2] H. Nishikawa et al. *Adv. Mater.* **2026**, *38*, e13451.

[S3] J. Xiang et al., *Adv. Mater*. **2015**, *27*, 3014.

[S4] C.-L. Yuan et al., *Sci. Adv*. **2019**, *5*, eaax9501.

[S5] C.-T. Xu et al., *Adv. Opt. Mater*. **2022**, *10*, 2201088

[S6] C.-L. Yuan et al., *Matter* **2023**, *6*, 3555.

[S7] T. Liu et al., *Adv. Funct. Mater*. **2024** ,*34*, 2408855.

[S8] O. D. Lavrentovich, *Opt. Mater. Express* **2020**, *10*, 2415.

[S9] C. Yuan et al., *Adv. Mater*. **2025**, *37*, 2507000.

[S10] J. Karcz et al., *Science* **2024**, *384*, 1096.

[S11] H. Nishikawa et al., *Adv. Sci*. **2024**, *11*, 2405718.

[S12] H. Nishikawa et al., *Adv. Mater.* **2026**, *38,* e13451.

[S13] F. Ye et al., *Small*, **2026**, *22*, e12407.
